# Association between risk of mortality among children and twin birth in India: an econometric analysis of live births between 1993–2021

**DOI:** 10.7189/jogh.15.04136

**Published:** 2025-05-05

**Authors:** William Joe, Atma Prakash, Komal Ahluwalia, Rockli Kim, S V Subramanian

**Affiliations:** 1 Institute of Economic Growth, New Delhi, India; 2 Division of Health Policy and Management, College of Health Science, Korea University, Seoul, South Korea; 3 Interdisciplinary Program in Precision Public Health, Department of Public Health Sciences, Graduate School of Korea University, Seoul, South Korea; 4 Harvard Center for Population and Development Studies, Cambridge, Massachusetts, USA; 5 Department of Social and Behavioral Sciences, Harvard T. H. Chan School of Public Health, Boston, Massachusetts, USA

## Abstract

**Background:**

Twin births present unique challenges for child survival, particularly in low- and middle-income countries. Despite the high burden of global child mortality, there has been no comprehensive assessment of twin births and deaths in India. We analysed the trends and patterns in twin births and deaths in India between 1993 and 2021, examining mortality risks across different phases of early childhood, including the late neonatal phase.

**Methods:**

We analysed data on 659 175 births from five rounds of India's National Family Health Survey (NFHS) (1992–93 to 2019–21). We calculated age-specific mortality rates using a synthetic cohort life table approach for early neonatal (0–7 days), late neonatal (8–28 days), post-neonatal (29 days to 11 months), and child (12–59 months) periods. The analysis employed logistic regression models (both adjusted and unadjusted) to estimate phase-specific mortality risks and coarsened exact matching to establish causal relationships while adjusting for demographic and socioeconomic covariates.

**Results:**

Twinning rates in India increased from 0.9% in 1992–93 to 1.5% in 2019–21. Despite this small share in births, twins accounted for 7.7% of under-five deaths in 2019-21. Twin under-five mortality rate was 179.8 (95% confidence interval (CI) = 162.2, 197.4) per 1000 live births in 2019–21, declining from 447.5 (95% CI = 405.6, 489.3) in 1992–93. Twins faced 7.5 times higher risk of early neonatal death and 10 times higher risk of late neonatal death compared to singletons. Twins from the poorest wealth quintile experienced 9.8 (95% CI = 8.43, 11.44) times higher early neonatal mortality risk compared to those from the highest quintile. The coarsened exact matching analysis confirmed twin birth as an independent risk factor for neonatal and infant mortality.

**Conclusions:**

Despite general improvements in child survival, twin mortality rates continue to be high, particularly during the neonatal period. The persistent socioeconomic gradient in twin survival necessitates strengthening health care delivery for vulnerable populations. Establishing twin registries and including twin mortality in global monitoring frameworks could accelerate progress toward achieving Sustainable Development Goal targets for child survival.

While twinning is uncommon, the global magnitude of twin births is substantial, with the average twinning rate across 75 developing countries estimated to be 1.3% [1]. There has been a notable rise in twinning frequencies across several countries in recent years. In the USA, twin births have increased from 1.9% in 1980 to 3.1% in 2021 [2]. In China, the twinning rate has climbed from 1.6% in 2007 to 2.2% in 2014 [3]. Several developed countries such as Denmark, UK (specifically, England and Wales), France, Germany, and South Korea have also experienced a 2-fold increase in twinning rates [4,5].

The twin registries in developed countries provide vital insights into the dynamics of twin births and deaths [6]. For example, studies have shown that twins encounter higher likelihoods of congenital abnormalities and neuro-cognitive disorders [7,8]. Twin pregnancies also lead to various obstetric complications (including maternal deaths), warranting comprehensive maternal and child health services [9,10]. However, despite progress in maternal and child health care, low- and middle-income countries (LMICs) display huge inequalities in access to emergency obstetric care [11]. Weak infrastructure and a shortage of general physicians and medical specialists (surgeons, obstetricians, and gynecologists) cumulatively add to the risk of maternal and child mortality in these countries [10,12].

The ‛twin effect’ on child mortality is a critical global health concern, as twins face a disproportionately higher risk of death compared to singletons. In sub-Saharan Africa, the under-five mortality among twins is estimated to be 213 per 1000 live births, accounting for 315 000 under-five deaths each year [13]. The sheer magnitude and burden of twin mortality in LMICs cannot be overlooked across large and populous settings. With increasing frequency of twinning, saving twin lives is a crucial, yet neglected concern to achieve the sustainable development goal (SDG) targets of reducing neonatal and under-five mortality rates to below 12 and 25 per 1000 births, respectively. The central promise of leaving no one behind under the 2030 Sustainable Development Agenda also necessitates a granular focus on such inequities and exclusions [14].

The attention on twin births and deaths holds immense relevance for India. Although the country recently rolled out universal programmes to improve maternal and child health, several challenges persist [15]. With a billion-plus population base, India adds large cohorts of births to the global population and accounts for an 18.7% share (0.9 million) in global under-five deaths [16]. While it is on course to achieve the SDG targets in under-five mortality, this progress conceals the inequities in the survival experiences of singletons and twins [17]. The evidence on health inequalities in India is also unapprised of socioeconomic patterning of twin births and deaths [18]. Our study addresses this gap by:

- examining the trends in twin mortality rates within India over the past three decades, contextualised against the documented increase in twin birth share;
- determining whether the disparity in mortality rates between singleton and twin births has narrowed over time;
- investigating socioeconomic disparities in twin mortality;
- analysing how quality of healthcare services correlates with twin birth prevalence, socioeconomic factors, healthcare access, and survival outcomes for twins compared to singletons.

To the best of our knowledge, this is the first analysis of twin mortality in India that utilises data from all five rounds of the National Family Health Survey (NFHS), as well as the first to directly compare twin and singleton mortality rates and analyse the birth share of twins across these survey rounds. The trends and patterns over the three-decade period of the survey would present comprehensive evidence on the phenomenon of twinning and the risk of twin mortality in India and would consequently inform and call for global actions for saving twin lives and accomplishing the targets and the promise of the 2030 Sustainable Development Agenda.

## METHODS

### Study design

For this analysis, we retrieved repeated cross-sectional nationally representative household survey data from the five rounds of the NFHS for the years 1992–93 (NFHS-1), 1998–99 (NFHS-2), 2005–06 (NFHS-3), 2015–16 (NFHS-4), and 2019–21 (NFHS-5) [19]. Subsequently, survey years will be denoted solely by the final year of the survey period. The NFHS provides in-depth information on key health indicators across India, including fertility, infant and child mortality, family planning, maternal and child health, and nutrition. Each round employs a two-stage stratified cluster sampling design, uses the latest available census of India as the sampling frame at the time of the survey, and is conducted using a nationally representative sample of households throughout the country. The NFHS adheres to the protocols of the global Demographic and Health Surveys (DHS) Program, which operates in 90 countries [20] and has received approval from the International Institute of Population Sciences Institutional Review Board for the survey protocol and questionnaires. All individuals provide informed consent before participating in the NFHS.

### Outcomes

Our primary objective was to assess the pattern of twin births and deaths over time with a particular focus on mortality and mortality rates during specific age periods *viz.* early-neonatal deaths (first 7 days), late-neonatal deaths (8–28 days), post-neonatal deaths (29 days to 11 months), and child deaths (12–59 months). We ascertained the risk of twin mortality *vis-à-vis* singleton mortality for the full sample, as well as for the sample stratified by household wealth index quintiles. Finally, we explored the association of age-specific deaths among under-five children across demographic and socioeconomic covariates.

### Covariates

We adjusted our analysis of risk of mortality for twins *vis-à-vis* singleton births for various socioeconomic and demographic factors. Based on a literature review, we considered the following individual and household characteristics: child sex (female or male), birth order (first-born, second or third-born, or higher birth order), maternal education (no schooling, grade 1–5, grade 6–8, grade 9–12, or grade 12 or above), maternal age at birth (<20, 20–24, 25–29, or ≥30 years), place of residence (urban or rural) and caste (scheduled caste, scheduled tribe, other backward caste, others, unknown, or no caste). However, in 1992–93, the responses for caste classification – ‛do not know’ and ‛no caste’ – were categorised under ‘other’, and in 1998–99, ‛do not know’ was unavailable for caste classification.

We also included the household wealth index (poorest, poor, middle, richer, or richest) as a marker of socioeconomic status. The wealth quintile indicator is provided in all except the two first rounds of the NFHS survey. It constructed using the principal component analysis in 1993 and 1999 (Appendix P1 in the **Online Supplementary Document**) [21], antenatal care visit (no visit, 1–3 visit, ≥4, or do not know/missing), place of delivery (respondents’ or other home, private facilities, public facilities, or do not know/missing), assistance during delivery (skilled provider, unskilled provider, or do not know/missing), and child birth size (larger, average, small, or do not know/missing).

### Data analysis

The NFHS elicits information on the month and year of birth of the child, survival status, and age of death for deceased children. The age at death is reported in number of days for deaths occurring within a one-month period, and in months and years if the death occurs between 2–23 months or 24–59 months, respectively. Based on this information, we applied a synthetic cohort lifetable approach to estimate separate age-specific mortality rates for singletons and twins [22] as follows: early-neonatal mortality rate (ENMR), late-neonatal mortality rate (LNMR), post-neonatal mortality rate (PNMR), and child mortality rate (CMR). We presented all mortality rate estimates as the number of deaths per 1000 live births.

We determined univariate and bivariate distributions of twin births and deaths to ascertain the temporal trends and patterns across socioeconomic categories. We conducted a series of adjusted and unadjusted logistic regressions to assess the association of twin status, with early neonatal, late neonatal, post-neonatal, and child mortality as the dependent variables, and deaths preceding five-year intervals excluded from the denominators. We evaluated the consistency of this association through models adjusted for child sex, birth order, maternal education, maternal age at birth, household wealth index, caste, and place of residence, further extending this analysis by incorporating variables such as antenatal care visits, place of delivery, type of delivery assistance, and child birth size. Additionally, we estimated the risk of early neonatal, late neonatal, post-neonatal, and child mortality across the household wealth index using both adjusted and unadjusted models. We presented the findings as odds ratios (ORs) and their 95% confidence interval (CIs).

Finally, we applied coarsened exact matching (CEM) with twins as the treated units and singletons as the control units, restricting the analysis to children with socioeconomic and demographic covariates [23,24]. The reduction in unmatched units suggests an effective balance between the groups, enhancing the reliability of the subsequent causal analysis by minimising potential confounding (Appendix P2 in the **Online Supplementary Document**). We used Stata, version 17.0 (StataCorp LLC, College Station, TX, USA) for our analysis.

## RESULTS

### Trends in twin mortality and twin birth rates in India

Our study population included all singleton and twin births occurring within the five years preceding each survey (659 574 births). After we excluded multiple births of higher order (172 cases) and births with missing maternal education data (227 cases) [13,25], the final analytical sample comprised 659 175 births across the five rounds of NFHS (Table S1 in the **Online Supplementary Document**). There were 60 411 births and 4933 deaths in 1993, 56 701 births and 4252 deaths in 1999, 51 529 births and 2867 deaths in 2006, 259 561 births and 11 859 deaths in 2016, and 230 973 births and 8653 deaths in 2021. The samples of twin deaths for the five rounds were 335, 244, 182, 822, and 629, respectively (Table S2 in the **Online Supplementary Document**). We found that across the five rounds, both singleton and twin births were skewed with a male bias in the distribution. The share of singletons in first birth order increased from 27.4% in 1993 to 39.4% in 2021, while during this period, higher birth order (≥4) singleton births decreased from 31.1% to 11.8%. Unlike singleton births, the bulk of the twin births were in higher birth order. In 1993, 53.7% of twin births were in the birth order four or above, though in recent years, the concentration of twin births has shifted to second or third birth order (58.6% in 2021). The distribution of singleton and twin births varies by maternal age. In 2021, 46.9% of the singleton births were among mothers aged ≥25 years, compared to 59.5% of twin births (**Table 1**).

We noted no major differences in socioeconomic pattern (maternal education, wealth index, or caste group) in singleton and twin births across the five rounds. Nevertheless, in 2021, the proportion of twin births has marginally increased among births in the highest wealth quintile (15.7% for singletons, 20.2% for twins 20.2%) and those in urban areas (26.6% for singletons, 30.9% for twins). Furthermore, respondents’ or other home deliveries for singletons and twins declined from 60% and 51.5% in 1993, respectively to 11.2% and 8% respectively in 2021, respectively. By 2021, most singleton births occurred in public facilities (62.2%), while twin births were almost equally distributed between private (45.5%) and public facilities (46.1%). For singletons and twins, respectively, the proportion of births attended by skilled providers increased markedly from 26% and 31.8% in 1993 to 88.9% and 92.3% in 2021. Regarding birth size, twins were consistently more likely to be reported as small compared to singletons across all survey rounds. In 2021, 29.9% of twins were reported as small compared to 10.3% of singletons, while 69.7% of singleton births were reported as average size compared to 55.7% of twins (**Table 1**).

**Table 1:** Percentage distribution of singleton and twin birth across covariates, 1993–2021*

	2021 (n = 230 937)		2016 (n = 259 561)		2006 (n = 51 529)		1999 (n = 56 701)		1993 (n = 60 411)	
	**Singleton**	**Twin**	**Singleton**	**Twin**	**Singleton**	**Twin**	**Singleton**	**Twin**	**Singleton**	**Twin**
**Child sex**										
Boy	117 740 (52)	1900 (50.6)	132 878 (52.2)	2189 (51.8)	26 398 (52.1)	391 (51.3)	29 032 (51.7)	429 (49.4)	30 633 (51.2)	449 (53.3)
Girl	109 475 (48)	1858 (49.4)	122 449 (47.8)	2045 (48.2)	24 351 (47.9)	389 (48.7)	26 837 (48.3)	403 (50.6)	28 906 (48.8)	423 (46.7)
**Child birth order**										
First	87 628 (39.4)	727 (21.5)	95 465 (39)	736 (17.3)	16 448 (30.6)	113 (12.2)	15 981 (28.2)	98 (12.4)	16 700 (27.4)	100 (9.3)
Second or third	109 936 (48.7)	2155 (58.6)	118 924 (47)	2318 (56.7)	22 322 (43.2)	389 (46.7)	24 131 (43.4)	362 (41.8)	25 033 (41.5)	356 (37)
Fourth or higher	29 651 (11.8)	876 (19.9)	40 938 (14)	1180 (26)	11 979 (26.2)	278 (41)	15 757 (28.4)	372 (45.8)	17 806 (31.1)	416 (53.7)
**Maternal age at birth in years**										
<20	19 753 (9.7)	214 (6.8)	24 021 (10.3)	246 (7)	7508 (17.8)	84 (12.9)	10 239 (21.2)	98 (14.5)	10 850 (20.5)	122 (16.1)
20–24	94 998 (43.4)	1194 (33.7)	108 934 (44.8)	1434 (36.5)	20 137 (41.3)	264 (30.8)	21 910 (39.5)	298 (36)	22 867 (38.3)	262 (27.7)
25–29	71 891 (31.2)	1380 (37.5)	77 398 (29.7)	1526 (34.6)	14 182 (25.6)	264 (36)	14 684 (24.7)	234 (26.7)	15 408 (24.2)	266 (27.2)
≥30	40 573 (15.7)	970 (22)	44 974 (15.2)	1028 (21.8)	8922 (15.3)	168 (20.3)	9036 (14.6)	202 (22.8)	10 414 (17)	222 (29)
**Maternal education**										
No schooling	50 269 (21.5)	808 (19.3)	80 128 (30.2)	1370 (31.6)	20 798 (50.2)	324 (50.7)	29 834 (57.1)	458 (59.3)	35 893 (65.5)	488 (64.8)
Grade 1–5	29 005 (12.2)	484 (11.5)	36 977 (13.9)	514 (11.9)	7298 (13.9)	106 (11.8)	8666 (14.9)	124 (14.9)	7917 (12.2)	148 (14.4)
Grade 6–8	40 963 (17.5)	652 (15.7)	45 604 (17.7)	674 (15.7)	7831 (14)	98 (11.8)	6766 (11.3)	86 (9.9)	6424 (9.8)	90 (9.5)
Grade 10–12	76 091 (33.3)	1166 (34.2)	69 170 (27.8)	1180 (27.4)	10 986 (17)	178 (18.4)	8328 (13.3)	122 (13)	7235 (9.8)	102 (8.5)
Grade 12 or above	30 887 (15.6)	648 (19.4)	23 448 (10.4)	496 (13.5)	3836 (4.9)	74 (7.2)	2275 (3.5)	42 (2.9)	2070 (2.7)	44 (2.8)
**Household wealth index**										
Poorest	61 939 (24.6)	970 (22.1)	67 544 (25.4)	1140 (26)	9078 (25.5)	122 (21.9)	10 833 (23.9)	178 (26.2)	11 324 (22.5)	158 (22)
Poorer	53203 (21.8)	828 (19.8)	60 546 (22)	878 (21.2)	9416 (22.4)	148 (21.9)	11 013 (22.6)	146 (21.4)	11 347 (21.9)	168 (26.7)
Middle	43 994 (19.6)	684 (17.8)	50 913 (19.9)	846 (18.7)	10 505 (19.8)	150 (20.5)	11 887 (20.3)	192 (20.7)	12 137 (20.9)	172 (20.4)
Richer	38 089 (18.)	662 (20.1)	42 235 (18.1)	696 (17.3)	11 123 (18)	168 (18.8)	12 154 (18.6)	168 (17.9)	13 375 (19.6)	174 (16.1)
Richest	29 990 (15.7)	614 (20.2)	34 089 (14.7)	674 (16.8)	10 627 (14.3)	192 (16.9)	9982 (14.7)	148 (13.8)	11 356 (15.1)	200 (14.8)
**Caste**										
Scheduled caste	46 749 (23.4)	720 (19.4)	48 277 (21.6)	768 (19.9)	9039 (20.8)	124 (17.9)	10 174 (19.7)	176 (25.4)	7776 (13.4)	106 (14.3)
Scheduled tribe	46 063 (10)	700 (10.1)	51 419 (10.5)	774 (10.7)	8277 (9.7)	106 (8.2)	8373 (9.9)	94 (6.2)	7785 (9.5)	74 (6.7)
OBC	86 823 (43.5)	1472 (45)	100 027 (44.1)	1714 (45.1)	16 483 (40.2)	260 (42.8)	15 720 (32.1)	228 (30.4)	.	.
Other	35 592 (17.8)	664 (19.9)	44 198 (19.4)	812 (20.8)	14 814 (26.3)	244 (27.9)	21 089 (37.1)	324 (36.2)	43 978 (77.1)	692 (79)
Don't know	1561 (0.9)	30 (0.8)	1659 (0.9)	16 (0.3)	220 (0.4)	0 (0)	513 (1.3)	10 (1.7)		
No Caste	10 427 (4.5)	172 (4.9)	9747 (3.5)	150 (3.2)	1916 (2.6)	46 (3.2)				
**Place of residence**										
Urban	45 887 (26.6)	868 (30.9)	60 266 (28.1)	1086 (27.9)	19 179 (25.4)	292 (24.8)	14 310 (22)	206 (21.2)	16 035 (22.6)	286 (22.9)
Rural	181 328 (73.4)	2890 (69.1)	195 061 (71.9)	3148 (72.1)	31 570 (74.6)	488 (75.2)	41 559 (78)	626 (78.8)	43 504 (77.4)	586 (77.1)
**Place of delivery**										
Respondents’ or other home	30 913 (11.2)	385 (8)	62 685 (20.9)	779 (16.6)	27 997 (61.3)	326 (46.7)	21 166 (38.6)	206 (25.9)	34 669 (60)	393 (51.5)
Private facilities	48 643 (26.3)	1366 (45.5)	52 903 (26.6)	1400 (38.9)	10 766 (20.4)	244 (32.9)	5118 (10.1)	82 (10.3)	5320 (8.8)	98 (9.1)
Public facilities	147 103 (62.2)	1994 (46.1)	138 967 (52.2)	2041 (44.1)	11 850 (18)	208 (20.3)	6138 (9.4)	122 (12.6)	7850 (11.8)	180 (17)
Don't know/missing	556 (0.2)	13 (0.3)	772 (0.3)	14 (0.5)	136 (0.3)	2 (0.2)	23 447 (41.9)	422 (51.3)	11 700 (19.5)	201 (22.4)
**Antenatal care visits**										
None	11 309 (4.7)	96 (2.1)	33 344 (12.3)	295 (7.3)	6763 (16.1)	53 (9.2)	10 584 (19.9)	119 (16.3)	17 061 (30.4)	227 (29.9)
1–3	60 738 (26.2)	516 (12.4)	65 395 (23.4)	564 (12)	12 916 (28)	96 (13.1)	11 627 (20.9)	131 (15.5)	16 891 (28.8)	205 (26.2)
4 or more visits	99 753 (44.7)	980 (27.4)	88 569 (38.1)	858 (21.7)	16 452 (26.1)	160 (17.7)	10 174 (17.3)	160 (16.9)	14 145 (21.8)	256 (23.6)
Don't know/missing	55 415 (24.4)	2166 (58.2)	68 019 (26.2)	2517 (59)	14 618 (29.8)	471 (60)	23 484 (42)	422 (51.3)	11 442 (19)	184 (20.3)
**Assistance during delivery**										
Skilled provider	197 383 (88.9)	3380 (92.3)	197 093 (80.6)	3464 (83.3)	26 140 (45.3)	497 (57.6)	14 015 (24.3)	233 (25.8)	16 661 (26)	326 (31.8)
Unskilled provider	29 777 (11.1)	378 (7.7)	58 080 (19.3)	766 (16.5)	24 527 (54.6)	281 (42.2)	18 484 (34)	177 (22.9)	31 228 (54.6)	349 (46.1)
Don't know/missing	55 (0)	0 (0)	154 (0.1)	4 (0.2)	82 (0.1)	2 (0.2)	23 370 (41.7)	422 (51.3)	11 650 (19.4)	197 (22)
**Birth size**										
Large	41 095 (18.8)	493 (13)	43 473 (19.2)	616 (16)	11 397 (23.3)	131 (17.6)	4618 (8.2)	20 (2.6)	6687 (11.2)	41 (4.3)
Average	160 400 (69.7)	2161 (55.7)	175 592 (67.4)	2134 (49.3)	28 424 (54.8)	296 (39.1)	19 688 (35.9)	197 (23.5)	30 569 (51.8)	325 (38.7)
Small	22 216 (10.3)	1046 (29.9)	29 959 (11.8)	1373 (32.3)	10 038 (20.5)	335 (41.2)	8176 (14.1)	193 (22.6)	10 191 (16.9)	300 (33.3)
Don't know/missing	3504 (1.2)	58 (1.4)	6303 (1.7)	111 (2.4)	890 (1.4)	18 (2.1)	23 387 (41.7)	422 (51.3)	12 092 (20.2)	206 (23.8)

OBC – other backward class

*Presented as n (%) unless specified otherwise.

### Disparity in mortality rates between singleton and twin births

For twins in 2021, we observed an ENMR of 113.9 (95% CI = 110.3, 117.6) and a LNMR of 29.2 (95% CI = 27.5, 31.0) per 1000 live births, respectively. After combining, we estimated the NMR to be 143.1 per 1000 live births among twins. In comparison, we found an ENMR of 19.0 (95% CI = 18.7, 19.2) and a LNMR of 3.7 (95% CI = 3.6, 3.8) per 1000 live births for singletons. The NMR has declined since the early 1990s, when twins had an ENMR of 230.6 (95% CI = 220.0, 241.6) and a LNMR of 105.5 (95% CI = 97.6, 113.8) per 1000 live births. In 2021, the overall under-five mortality rate among twins was 179.8 (95% CI = 162.2, 197.4) per 1000 live births. The risks were substantially higher, but the mortality rates have declined from the 1992–93 NFHS levels, where almost every second twin born died (under five mortality rates of 447.5; 95% CI = 405.64, 489.28). The infant mortality rate for twins was estimated to be 169.7 per 1000 live births in 2021 compared to 429.2 per 1000 live births in 1993 (**Table 2**). We noted relatively faster reductions in twin mortality rates in the 1990s compared to recent decades.

**Table 2:** Early-neonatal, late-neonatal, post-neonatal, child and under-five mortality rates among singletons and twins, India

	Early-neonatal mortality rate (95% CI)	Late-neonatal mortality rate (95% CI)	Post-neonatal mortality rate (95% CI)	Child mortality rate (95% CI)	Under-five mortality rate (95% CI)
**2021**					
Overall	20.60 (20.34, 20.86)	4.16 (4.03, 4.28)	10.66 (10.66, 10.66)	8.41 (7.35, 9.48)	43.53 (42.09, 44.97)
Singleton	18.96 (18.71, 19.22)	3.72 (3.60, 3.84)	10.38 (10.38, 10.38)	8.36 (7.30, 9.42)	41.14 (39.72, 42.57)
Twin	113.90 (110.28, 117.63)	29.19 (27.49, 30.99)	26.57 (26.57, 26.58)	12.23 (−0.16, 24.61)	179.81 (162.21, 197.42)
**2016**					
Overall	24.31 (24.04, 24.59)	4.78 (4.65, 4.91)	11.52 (11.52, 11.52)	11.24 (10.00, 12.49)	51.40 (49.84, 52.97)
Singleton	22.62 (22.36, 22.89)	4.33 (4.21, 4.46)	11.04 (11.04, 11.05)	11.25 (9.99, 12.51)	48.82 (47.25, 50.38)
Twin	125.06 (122.1, 128.08)	31.51 (29.78, 33.33)	40.87 (40.86, 40.88)	10.67 (4.17, 17.16)	206.00 (191.92, 220.08)
**2006**					
Overall	29.65 (28.98, 30.34)	8.51 (8.15, 8.88)	18.54 (18.53, 18.54)	18.99 (16.05, 21.94)	74.61 (70.80, 78.43)
Singleton	27.90 (27.24, 28.57)	7.98 (7.63, 8.35)	17.68 (17.68, 17.68)	18.92 (15.94, 21.89)	71.47 (67.66, 75.28)
Twin	139.62 (129.09, 150.85)	41.67 (36.27, 47.83)	73.49 (73.47, 73.51)	25.22 (6.9, 43.53)	273.56 (232.12, 315)
**1999**					
Overall	31.59 (30.99, 32.2)	10.74 (10.40, 11.10)	25.02 (25.02, 25.03)	34.05 (30.29, 37.8)	99.11 (94.86, 103.36)
Singleton	29.49 (28.9, 30.09)	10.22 (9.88, 10.57)	24.17 (24.17, 24.17)	34.23 (30.44, 38.01)	95.92 (91.65, 100.19)
Twin	175.25 (165.54, 185.4)	46.26 (41.4, 51.65)	83.22 (83.2, 83.24)	17.55 (2.43, 32.67)	316.93 (278.72, 355.13)
**1993**					
Overall	33.13 (32.51, 33.76)	14.04 (13.65, 14.45)	30.05 (30.05, 30.05)	38.82 (34.39, 43.26)	113.05 (108.21, 117.88)
Singleton	30.18 (29.58, 30.78)	12.68 (12.30, 13.07)	29.09 (29.09, 29.10)	38.88 (34.41, 43.34)	108.03 (103.19, 112.87)
Twin	230.63 (220.00, 241.62)	105.47 (97.65, 113.83)	93.15 (93.12, 93.17)	31.91 (5.72, 58.11)	447.46 (405.64, 489.28)

We applied a logistic regression model to the overall sample, while the CEM matched sample presented the ORs for the risk of mortality for twin births with reference to singletons. The unadjusted models for the overall sample suggested that twins were 6.6 times more likely to die in the early neonatal phase (first seven-day period) compared to singletons (OR = 6.62; 95% CI = 5.74, 7.62). The high risk of early neonatal deaths among twins since 1993 (OR = 9.53; 95% CI = 7.75, 11.72) persisted throughout the last three decades. The models adjusted for various demographic and socioeconomic correlates suggested a similar likelihood of mortality among twins in the early neonatal phase. The adjusted models showed that, in 1993, twins were 10.7 times more likely than singleton to die in the early neonatal phase, with a 7.6 times higher risk observed in 2019–21. The CEM sample-based adjusted models also showed a similar likelihood of early neonatal death for twins (1993: OR = 8.52; 95% CI = 6.89, 10.53 and 2021: OR = 7.53; 95% CI = 6.71, 8.46) (**Table 3**).

**Table 3:** ORs/aORs and 95% CIs associated with twin birth on early-neonatal, late-neonatal, post-neonatal, and child mortality (1993, 2021) based on conventional logistic regression and logistic regression with CEM

	Early-neonatal mortality rate	Late-neonatal mortality rate	Post-neonatal mortality rate	Child mortality rate
**Twin *vs*. singleton (ref)**				
2021				
*OR (95% CI)*	6.62 (5.74, 7.62)	8.92 (6.94, 11.47)	2.91 (2.26, 3.74)	1.19 (0.48, 2.99)
*aOR (95% CI)*	7.62 (6.60, 8.79)	10.02 (7.71, 13.02)	3.15 (2.44, 4.06)	1.28 (0.50, 3.26)
2016				
*OR (95% CI)*	6.14 (5.45, 6.92)	8.3 (6.65, 10.35)	4.27 (3.50, 5.21)	1.04 (0.56, 1.92)
*aOR (95% CI)*	6.95 (6.16, 7.85)	8.6 (6.87, 10.76)	4.25 (3.48, 5.20)	1.03 (0.55, 1.90)
2006				
*OR (95% CI)*	5.63 (4.28, 7.41)	6.18 (3.97, 9.62)	5.17 (3.52, 7.59)	1.95 (0.91, 4.18)
*aOR (95% CI)*	6.62 (5.01, 8.74)	7.01 (4.47, 10.99)	5.68 (3.85, 8.37)	2.07 (0.95, 4.51)
1999				
*OR (95% CI)*	7.13 (5.72, 8.89)	5.57 (3.73, 8.31)	4.69 (3.45, 6.39)	0.82 (0.33, 2.00)
*aOR (95% CI)*	7.5 (6.00, 9.38)	5.83 (3.91, 8.70)	4.8 (3.49, 6.59)	0.76 (0.31, 1.87)
1993				
*OR (95% CI)*	9.53 (7.75, 11.72)	12.11 (9.13, 16.06)	5.13 (3.77, 6.99)	1.04 (0.44, 24.4)
*aOR (95% CI)*	10.73 (8.68, 13.25)	13.83 (10.33, 18.52)	5.27 (3.83, 7.27)	1.01 (0.43, 2.35)
**Coarsened exact matching**				
2021				
*OR (95% CI)*	7.39 (6.58, 8.29)	8.81 (6.88, 11.28)	3.16 (2.53, 3.94)	1.15 (0.66, 2.03)
*aOR (95% CI)*	7.53 (6.71, 8.46)	9.14 (7.15, 11.67)	3.3 (2.64, 4.11)	1.21 (0.69, 2.14)
2016				
*OR (95% CI)*	7.16 (6.48, 7.92)	9.05 (7.43, 11.02)	4.27 (3.61, 5.05)	0.96 (0.58, 1.58)
*aOR (95% CI)*	7.29 (6.59, 8.07)	9.39 (7.71, 11.45)	4.4 (3.72, 5.21)	1.01 (0.62, 1.67)
2006				
*OR (95% CI)*	7.18 (5.53, 9.31)	6.21 (3.83, 10.06)	3.94 (2.72, 5.71)	2.9 (1.41, 5.95)
*aOR (95% CI)*	7.44 (5.71, 9.70)	6.5 (4.00, 10.58)	4.28 (2.94, 6.25)	3.21 (1.61, 6.42)
1999				
*OR (95% CI)*	8.03 (6.44, 10.02)	4.46 (2.92, 6.80)	5.04 (3.75, 6.76)	0.69 (0.32, 1.48)
*aOR (95% CI)*	8.21 (6.55, 10.31)	4.64 (3.04, 7.09)	5.28 (3.89, 7.15)	0.7 (0.32, 1.51)
1993				
*OR (95% CI)*	8.12 (6.51, 10.13)	12.47 (9.57, 16.25)	5.11 (3.93, 6.64)	1.01 (0.49, 2.09)
*aOR (95% CI)*	8.52 (6.89, 10.53)	13.72 (10.45, 18.01)	5.55 (4.23, 7.27)	1.13 (0.55, 2.33)

aOR – adjusted odds ratio, CEM – coarsened exact matching, CI – confidence interval, OR – odds ratio, ref – reference

The mortality risk among twins compared to singletons in the late neonatal phase (8–28 days period) is even higher than the likelihood in the early neonatal phase. In adjusted logistic regression models for the overall and CEM samples, twins showed mortality ORs of 10.02 (95% CI = 7.71, 13.02) and 9.14 (95% CI = 7.15, 11.67), respectively, when compared to singletons in the late neonatal phase. The unadjusted and adjusted models indicated mortality odds ratios of 12.11 (95% CI = 9.13, 16.06) and 13.83 (95% CI = 10.33, 18.52) during 1993 (**Table 3**). The CEM sample models showed similar mortality patterns. While the mortality differences between twins and singletons decreased during the post-neonatal phase, the OR remained three times higher for twins. However, these differences were not statistically significant during the 12–59-month period.

### Disparity in mortality rates between singleton and twin births

The survival of twins in the early and late neonatal phase was significantly influenced by their household’s socioeconomic status. The pooled analysis of years 2016 and 2021 confirmed the socioeconomic gradient. The logistic regression model based on the overall sample showed that twins from poorer households were 9.82 (95% CI = 8.43, 11.44) and 10.36 (95% CI = 7.86, 13.64) times more likely to die in early neonatal and late neonatal phase, respectively, while the OR for the highest wealth quintile was 3.44 (95% CI = 2.41, 4.92) and 3.60 (95% CI = 1.81, 7.15), respectively. We observed no significant survival advantage by socioeconomic status during the post-neonatal phase or the child mortality phase. The estimates from the CEM sample models confirm these inferences (Table S4 in the **Online Supplementary Document**).

The overall share of twins in total births in India increased from 0.9% in 1993 to 1.5% in 2021. Across the NFHS, the phase-wise (early neonatal, late neonatal, post-neonatal, child) distribution of under-five deaths shows an increasing concentration of deaths in the early neonatal phase. Importantly, in comparison to births, twins account for a much higher share of total under-five deaths. In 2021, for example, 7.7% of the total under-five deaths were among twins. The share of twins in under-five deaths showed a fluctuating, but generally increasing trend over the past three decades. Starting at 7.2% in 1993, it decreased to 5.7% in 1999, before rising steadily through subsequent survey rounds to reach 6.4% in 2006, 7.3% in 2016, and 7.7% in 2021 **(**Table S3 in the **Online Supplementary Document****)**.

We further analysed demographic and socioeconomic correlates associated with the risk of childhood mortality (Table S5–8 in the **Online Supplementary Document**). In the early neonatal phase, the likelihood of mortality among male children (2019–21: OR = 1.24; 95% CI = 1.15, 1.34) was higher than in female ones, with the pattern being consistent across all five NFHS rounds. The likelihood of mortality for second or third birth order (2021: OR = 0.69; 95% CI = 0.63, 0.76) and fourth or higher birth order (2021: OR = 0.88; 95% CI = 0.75, 1.02) is significantly smaller than first birth order. We also noted a higher likelihood of mortality for children with teenage mothers (2021: OR = 1.27; 95% CI = 1.08, 1.49) compared to those in the ≥30-year-old age group.

Further, the socioeconomic gradient in early neonatal mortality was significant. In 2021, children from poorest households were 2.16 times (95% CI = 1.77, 2.64) more likely to die. The wealth gradient in early neonatal mortality increased from 1993 to 2021. We noted a similar pattern of early neonatal phase survival disadvantage for children with mothers without any formal schooling. Scheduled caste households were more disadvantaged in early neonatal survival than other caste groups. In 1993, newborns from such households were 1.23 times (95% CI = 1.06, 1.44) more likely to die in neonatal phase than children from other caste groups. The relative risks remained the same in 2021 (OR = 1.22; 95% CI = 1.07, 1.40). Besides, under-five children from rural areas were also consistently disadvantaged in early neonatal survival (Table S5 in the **Online Supplementary Document**).

The demographic and socioeconomic patterns in risk of late-neonatal, post-neonatal, and child mortality phases correspond with the risk profile of the early neonatal phase, though a few exceptions are worth noting (Table S6–8 in the **Online Supplementary Document**). Specifically, gender differential in terms of male disadvantage in mortality were insignificant in late- and post- neonatal phase, as were the disadvantages for the scheduled caste and place of residence. The CEM estimates showed consistent patterns in the associations between demographic and socioeconomic factors and under-five deaths across various phases (Table S11–14 in the **Online Supplementary Document**). In the CEM sample models, the ORs for male mortality in the early neonatal phase (2021: OR = 1.33; 95% CI = 1.20, 1.46) were higher than in the overall sample. Similarly, the ORs for rural areas in the CEM sample (2021: OR = 1.41; 95% CI = 1.17, 1.71) exceeded those from the overall sample model (2021: OR = 1.10; 95% CI = 0.97, 1.24) (Table S11 in the **Online Supplementary Document**). Further sensitivity of mortality risk by conducting separate logistic regression models for each wealth quintile finds that lack of maternal education in the poorest households is a critical determinant of post-neonatal mortality whereas such risk is insignificant in the highest wealth quintile (Table S12–14 in the **Online Supplementary Document**).

## DISCUSSION

Our analysis provides novel insights on the trends and patterns of twin births and deaths in India. The five key findings are as follows. First, the twinning rates have increased in India from 0.9% in 1993 to 1.5% in 2021. The pattern was consistent with global trends in twin births [1]. Second, despite a small share in total births, twins accounted for 7.7% of the total under-five deaths in India. The mortality rates among twins have declined over time, but the levels remained large, remaining at 179.8 per 1000 live births in 2021. The resulting magnitude of such deaths can be very large for a populous country such as India, where the share of twins in under-five deaths is also increasing since the 1990s. Third, the risk of early neonatal mortality among twins was 7.5 times higher than among singletons. The relative risks continued to be of similar magnitude as in the early 1990s. While the LNMR was lower than the ENMR, the likelihood of mortality among twins in the late-neonatal phase was 10 times higher than that for the singletons. Fourth, the survival of twins during early-, late- and post-neonatal phases showed significant socioeconomic gradient by household wealth status. Such inequities in twin mortality risk call for a need to classify twins as a vulnerable group deserving greater attention for their survival and health. Finally, we note that twin births were more concentrated in higher birth order, but twin deaths are more likely among twins of first or second birth order. The CEM analysis confirmed that twin birth is an independent risk factor in child mortality and poses huge risks to child survival during the neonatal phase.

Twinning rates have been increasing globally, and India is no exception [5]. Here, we estimated the rate to be 0.09% for the year 1993, which is slightly higher than previous estimates (0.07% twins in total births) [1]. The difference could be attributed to the use of a longer birth history recall (10 years) in Smits and Monden [1] compared to the five-year reference used in our analysis. The use of a year's reference period is customary to avoid recall bias, it also facilitates the analysis of twin mortality for the sample. Studies have also observed an upward trend in the twinning rates in India throughout the 1990s [1,5], which could be partly attributable to the increasing maternal age at childbirth [19,26]. Notwithstanding such biological tendencies, the increasing use of ART is a major factor underlying the upward trends observed globally [4,27]. Ovarian hyperstimulation, embryo biopsy, and transfer of more than one embryo during in vitro fertilisation greatly increase the chances of twinning or multiple births [28,29]. The proliferation of ART centres from metropolitan to smaller cities in India could also, in part, explain the rising twinning rates in the country [30].

Twins face poorer survival rates than singletons, with those from LMICs facing even higher risks of mortality, and our estimates show the severity of the situation in India, where twins face a seven-fold increase in the risk of early neonatal mortality compared to singletons. The hazard for Indian twins compared to singletons is higher than in Eastern, Southern, or sub-Saharan Africa, where twins confront a five times higher risk of neonatal mortality [13,31,32]. The risks are higher than the situation of twins in England and Wales in the 1950s, who were at five times higher risk of neonatal mortality than singletons [26]. The under-five mortality rate among Indian twins in India (317 per 1000 live births for births during 2011–16) was higher than in sub-Saharan Africa (213 per 1000 live births for births during 2009–14) during comparable periods [33]. Nevertheless, the risk patterns for twins in India and Africa were similar during the post-neonatal phase, and in both contexts, the differential risks of child mortality were statistically insignificant. Explicit analyses of sub-Saharan Africa showed that mortality risk convergence for twins and singletons occurs at about six years of age, while prior to that, twins continue to be at a disadvantage [33]. However, ours is the first study to estimate the mortality risk experienced during the late neonatal phase (8–28 days). It is important to note that the risk of late neonatal mortality among twins is greater than in the early neonatal phase. This is in stark contrast with the survival experience of singletons in India, where the bulk of the deaths are concentrated in the early neonatal period [22]. The global evidence on the risk to singleton lives also shows higher mortality during the early neonatal phase than the late neonatal phase [34]. Although a few studies suggest a higher risk of infection in the home environment as a probable cause, further evidence on the risk of twin mortality in the early and late neonatal phase is warranted [34,35].

The risk patterns of twin mortality related to demographic and socioeconomic factors observed in our study are consistent with other studies [13,31,32]. For instance, the survival rate among male twins was lower than that of females and a low economic status of the household also emerged as a risk factor in twin mortality [36]. In the Indian context, twins from vulnerable groups, such as the scheduled castes, were previously found to be at a higher risk than other groups [37].

A high risk of twin mortality, when combined with large birth cohorts, can pose several challenges for achieving faster reductions in the global burden of neonatal and under-five deaths worldwide. It is disconcerting that with only a 1.5% share in total births, twins accounted for a 7.7% share in total under-five deaths per our analysis. This is a critical finding, as similar patterns have also been noted in other countries. In Gambia, twins account for 11.8% and 7.8% share in neonatal and post-neonatal deaths, respectively [31]. In sub-Saharan Africa, twins account for 11% of under-five deaths, and this proportion has risen since the 1990s [13]. An analysis of 60 LMICs revealed that early neonatal mortality among twins was significantly higher when compared to that of singleton neonates [16]. We also found a similar upward trend in the share of twins in total under-five deaths in India. The twin mortality rates also vary by the development profile of the countries. In China, neonatal mortality rates among twins were 15.7 deaths per 1000 twin births, compared to 213 in sub-Saharan Africa [3,13]. The twin mortality rates for India are also likely to be on the higher side. This calls for specific policy interventions to identify and monitor twin births and extend necessary health care support through institutional or community-level initiatives.

In the past, community-centric efforts have been successful in India and have led to the scaling up of the home-based newborn care programme [38]. Specific training to frontline health workers on managing and monitoring the health and growth of twins can enhance birth care capacities at the grassroots level. Line-listing of twin pregnancies, tracking, and review of such pregnancies and their outcomes through dedicated platforms is also necessary [39]. Antenatal care services, use of ultrasonography tools, and delivery planning should be holistic, including a clear mapping of emergency obstetric care facilities to avoid maternal or neonatal deaths [9,40]. Strengthening of pregnancy and birth registries and its utilisation to support pregnant women and her nodal frontline workers is also required [41]. While efforts to establish comprehensive maternal and newborn birth registries have been noted – for example, in Belgavi and Nagpur in India – it is necessary to further scale up such initiatives [6,42]. Countries such as Australia, China, Denmark, Finland, Sri Lanka, and Sweden, among others, have established specific twin registries for monitoring these outcomes long ago and have been engaged in research on various facets of health and developmental milestones [43–49]. These registries also allow us to determine several important developmental insights from an economic perspective and can facilitate development policymaking in LMICs [50,51]. The greatest contribution of these registries, however, was that they allow us to explore and address the physiological complexities underlying twinning and twin survival worldwide. Thus, tackling the challenge of twin pregnancies and births in terms of child survival is vital for India.

The inclusion of twin mortality concerns in the SDG global indicator framework can also prompt concerted policy action across countries. As apparent from the experience of the developed world, twin mortality is preventable and can accelerate national and global progress toward the SDGs. To bolster these efforts, we emphasise the need to improve the health management information system infrastructure to systematically capture twin birth data, including mandatory twin birth reporting and structured follow-up protocols, as well as expand their indicators to regularly track multiple births with specific metrics. Establishing a comprehensive registry for ART births, coordinated by the Indian Council of Medical Research or another nodal institution, is essential for collecting detailed data on the number of multiple births, gender and geographical birth, type of birth (vaginal/caesarean), maternal health indicators, and birth outcomes, which would then allow for better monitoring of trends and targeted interventions. Given the increased risks associated with twin pregnancies, strengthening antenatal care services, with a focus on early detection of twin pregnancies and specialised care pathways, is also critical. Increased availability of skilled birth attendants and improved access to emergency obstetric care, especially caesarean sections when indicated, are vital. Birth planning education should be a priority. Leveraging community health workers to provide targeted support to high-risk households with twin births is also recommended, as they can play a crucial role in providing education on infant care, nutrition, early detection of complications, and the importance of antenatal care. We emphasise a collaborative approach involving government institutions, academic institutions, health care providers, and research organisations to test the suitability and effectiveness of different data collection and management systems, and intervention strategies. We also suggest the use of targeted educational campaigns to help dispel myths and provide accurate information regarding twin pregnancies. These actionable steps, grounded in both data-driven strategies and evidence-based interventions, would contribute to a reduction in twin mortality and progress toward SDG targets.

We should outline some limitations of our approach. The survey data on twins arguably provides an underestimate of the overall levels in twin births and deaths. The reasons are twofold: in developing country contexts, the probability of maternal mortality in case of twin births is higher, and hence such information may be underestimated in the survey. More precisely, the risk of stillbirth is higher for either of the twins and the surviving child is likely to be categorised as a singleton in the survey data [52]. The recall of date of birth and death by the respondents may be sensitive and are based on maternal reports, thus, depending on the magnitude, this may have affected our insights regarding the phases of mortality. Such recall errors are likely to reduce through improvements of health records and with the higher education levels of the respondents. Our interpretation, therefore, places more emphasis on recent survey data to draw the conclusions on twin births and deaths. While reporting bias is more pronounced for deaths occurring beyond five years, the recall period of 0 to 4 years used in our study is largely unaffected [22,53]. The sample of twin deaths was relatively small especially in the early 1990s (NFHS rounds 1–3), making it important to validate such statistics through national registries on births and deaths. Besides, the cross-sectional design of the NFHS does not allow for causal analysis of mortality risks with socioeconomic factors. We attempted to address this through our CEM analysis. The CEM sample in our analysis provides consistent estimates as regression models based on the full sample. Nevertheless, more controls on health status and health care practices may be necessary to further determine the independent risks of twinning on childhood mortality in India.

Another limitation of this study is the observed higher late neonatal mortality risk among twins compared to early neonatal risk, which contradicts global patterns for singletons [54]. While we hypothesise that home care conditions during the late neonatal period may have contributed to this finding, further research is necessary to confirm this and explore other potential factors. Furthermore, there is the lack of detailed data on the usage of assisted reproductive technology (ART) within the DHS. Consequently, we were unable to provide specific insights into such trends in India or analyse their potential impact on twinning rates across different socioeconomic strata. 

## CONCLUSIONS

Despite notable advancements in overall child survival, twins remain a highly vulnerable population, facing a significantly elevated risk of mortality, particularly during the neonatal period. The documented increase in twinning rates, coupled with the disproportionate contribution of twins to under-five deaths, underscores the urgency for targeted interventions. The observed socioeconomic gradients in twin mortality further emphasize the necessity of classifying twins as a vulnerable group deserving focused attention and dedicated resources. The mortality patterns observed in the late neonatal phase, contrasting with global singleton trends, warrant further investigation. To mitigate these disparities and improve twin survival, this study recommends several key actions: first, enhancing the HMIS and establishing a dedicated ART registry are crucial for accurate data collection and monitoring. Second, strengthening ANC, ensuring equitable access to skilled birth attendants and emergency obstetric care, and effectively leveraging community health workers are essential strategies for improving twin survival. The establishment of comprehensive twin registries, mirroring successful models in developed nations, would facilitate more effective monitoring, targeted research, and evidence-based policy development. Ultimately, integrating twin mortality concerns into the Sustainable Development Goal framework and implementing the evidence-based interventions outlined in this research are vital to accelerate progress towards reducing neonatal and under-five deaths in India and globally.

## Additional material


Online Supplementary Document

## References

[1] Smits J Monden C Twinning across the Developing World. PLoS One. 2011;6:e25239. 10.1371/journal.pone.0025239 21980404 PMC3182188

[2] Osterman MJK Hamilton BE Martin JA Driscoll AK Valenzuela CP Births: Final Data for 2021. Natl Vital Stat Rep. 2023;72:1–53. 36723449

[3] Deng C Dai L Yi L Li X Deng K Mu Y Temporal trends in the birth rates and perinatal mortality of twins: A population-based study in China. PLoS One. 2019;14:e0209962. 10.1371/journal.pone.0209962 30650106 PMC6334899

[4] Pison G Monden C Smits J Twinning Rates in Developed Countries: Trends and Explanations. Popul Dev Rev. 2015;41:629–49. 10.1111/j.1728-4457.2015.00088.x

[5] Monden C Pison G Smits J Twin Peaks: more twinning in humans than ever before. Hum Reprod. 2021;36:1666–73. 10.1093/humrep/deab029 33709110 PMC8129593

[6] Hur YM Bogl LH Ordoñana JR Taylor J Hart SA Tuvblad C Twin Family Registries Worldwide: An Important Resource for Scientific Research. Twin Res Hum Genet. 2019;22:427–37. 10.1017/thg.2019.121 31937381 PMC7301690

[7] Glinianaia SV Rankin J Wright C Congenital anomalies in twins: a register-based study. Hum Reprod. 2008;23:1306–11. 10.1093/humrep/den104 18387962

[8] Pharoah PO Adi Y Consequences of in-utero death in a twin pregnancy. Lancet. 2000;355:1597–602. 10.1016/S0140-6736(00)02215-7 10821363

[9] Rao A Sairam S Shehata H Obstetric complications of twin pregnancies. Best Pract Res Clin Obstet Gynaecol. 2004;18:557–76. 10.1016/j.bpobgyn.2004.04.007 15279817

[10] Vogel JP Torloni MR Seuc A Betrán AP Widmer M Souza JP Maternal and perinatal outcomes of twin pregnancy in 23 low- and middle-income countries. PLoS One. 2013;8:e70549. 10.1371/journal.pone.0070549 23936446 PMC3731264

[11] Mahato PK van Teijlingen E Simkhada P Angell C Determinants of quality of care and access to basic emergency obstetric and neonatal care facilities and midwife-led facilities in low and middle-income countries: a systematic review. Journal of Asian Midwives. 2018;4:25–61.

[12] Hoyler M Finlayson SR McClain CD Meara JG Hagander L Shortage of doctors, shortage of data: a review of the global surgery, obstetrics, and anesthesia workforce literature. World J Surg. 2014;38:269–80. 10.1007/s00268-013-2324-y 24218153

[13] Monden CWS Smits J Mortality among twins and singletons in sub-Saharan Africa between 1995 and 2014: a pooled analysis of data from 90 Demographic and Health Surveys in 30 countries. Lancet Glob Health. 2017;5:e673–9. 10.1016/S2214-109X(17)30197-3 28578941

[14] United Nations. Transforming our World: The 2030 Agenda for Sustainable Development, A/RES/70/1. New York, NY, USA: United Nations General Assembly; 2015. Available: https://docs.un.org/en/A/RES/70/1. Accessed: 3 April 2025.

[15] Paul VK Sachdev HS Mavalankar D Ramachandran P Sankar MJ Bhandari N Reproductive health, and child health and nutrition in India: meeting the challenge. Lancet. 2011;377:332–49. 10.1016/S0140-6736(10)61492-4 21227494 PMC3341742

[16] GBD 2016 Mortality Collaborators Global, regional, and national under-5 mortality, adult mortality, age-specific mortality, and life expectancy, 1970-2016: a systematic analysis for the Global Burden of Disease Study 2016. Lancet. 2017;390:1084–150. 10.1016/S0140-6736(17)31833-0 28919115 PMC5605514

[17] Subramanian SV Ambade M Kumar A Chi H Joe W Rajpal S Progress on Sustainable Development Goal indicators in 707 districts of India: a quantitative mid-line assessment using the National Family Health Surveys, 2016 and 2021. Lancet Reg Health Southeast Asia. 2023;13:100155. 10.1016/j.lansea.2023.100155 37383562 PMC10306006

[18] Jain A Kim R Swaminathan S Subramanian SV Socioeconomic inequality in child health outcomes in India: analyzing trends between 1993 and 2021. Int J Equity Health. 2024;23:149. 10.1186/s12939-024-02218-z 39085858 PMC11290299

[19] International Institute for Population Sciences, The International Classification of Functioning, Disability and Health. National Family Health Survey (NFHS-5), 2019-21. Mumbai, India: International Institute for Population Sciences, Mumbai; 2021. Available: https://dhsprogram.com/pubs/pdf/FR375/FR375.pdf. Accessed: 3 April 2025.

[20] Corsi DJ Neuman M Finlay JE Subramanian SV Demographic and health surveys: a profile. Int J Epidemiol. 2012;41:1602–13. 10.1093/ije/dys184 23148108

[21] Filmer D Pritchett LH Estimating wealth effects without expenditure data–or tears: an application to educational enrollments in states of India. Demography. 2001;38:115–32. 11227840 10.1353/dem.2001.0003

[22] Subramanian SV Kumar A Pullum TW Ambade M Rajpal S Kim R Early-Neonatal, Late-Neonatal, Postneonatal, and Child Mortality Rates Across India, 1993-2021. JAMA Netw Open. 2024;7:e2410046. 10.1001/jamanetworkopen.2024.10046 38728034 PMC11087840

[23] Blackwell M Iacus S King G Porro G cem: Coarsened exact matching in Stata. Stata J. 2009;9:524–46. 10.1177/1536867X0900900402

[24] Iacus SM King G Porro G Causal inference without balance checking: Coarsened exact matching. Polit Anal. 2012;20:1–24. 10.1093/pan/mpr013

[25] Croft TN, Marshall AMJ, Allen CK, Arnold F, Assaf S, Balian S. Guide to DHS Statistics. Rockville, Maryland, USA: The International Classification of Functioning, Disability and Health; 2018. Available: https://dhsprogram.com/pubs/pdf/DHSG1/Guide_to_DHS_Statistics_DHS-7.pdf. Accessed: 3 April 2025.

[26] Bulmer MG. The Biology of Twinning in Man. Oxford, UK: Oxford University Press; 1970. Available: https://genepi.qimr.edu.au/contents/p/staff/1970_BulmerBiologyOfTwinning.pdf. Accessed: 3 April 2025.

[27] Kupka MS Ferraretti AP de Mouzon J Erb K D’Hooghe T Castilla JA Assisted reproductive technology in Europe, 2010: results generated from European registers by ESHRE. Hum Reprod. 2014;29:2099–113. 10.1093/humrep/deu175 26740578

[28] Kamath MS Antonisamy B Sunkara SK Zygotic splitting following embryo biopsy: a cohort study of 207 697 single-embryo transfers following IVF treatment. BJOG. 2020;127:562–9. 10.1111/1471-0528.16045 31828906

[29] Fauser BC Devroey P Macklon NS Multiple birth resulting from ovarian stimulation for subfertility treatment. Lancet. 2005;365:1807–16. 10.1016/S0140-6736(05)66478-1 15910954

[30] Malhotra N Shah D Pai R Pai HD Bankar M Assisted reproductive technology in India: A 3 year retrospective data analysis. J Hum Reprod Sci. 2013;6:235–40. 10.4103/0974-1208.126286 24672161 PMC3963305

[31] Miyahara R Jasseh M Mackenzie GA Bottomley C Hossain MJ Greenwood BM The large contribution of twins to neonatal and post-neonatal mortality in The Gambia, a 5-year prospective study. BMC Pediatr. 2016;16:39. 10.1186/s12887-016-0573-2 26979832 PMC4791939

[32] Justesen A Kunst A Postneonatal and child mortality among twins in Southern and Eastern Africa. Int J Epidemiol. 2000;29:678–83. 10.1093/ije/29.4.678 10922345

[33] Pongou R Shapiro D Tenikue M Mortality convergence of twins and singletons in sub-Saharan Africa. Demogr Res. 2019;41:1047–58. 10.4054/DemRes.2019.41.36

[34] Dare S Oduro AR Owusu-Agyei S Mackay DF Gruer L Manyeh AK Neonatal mortality rates, characteristics, and risk factors for neonatal deaths in Ghana: analyses of data from two health and demographic surveillance systems. Glob Health Action. 2021;14:1938871. 10.1080/16549716.2021.1938871 34308793 PMC8317945

[35] Leach A McArdle TF Banya WA Krubally O Greenwood AM Rands C Neonatal mortality in a rural area of The Gambia. Ann Trop Paediatr. 1999;19:33–43. 10.1080/02724939992617 10605518

[36] Pongou R Why is infant mortality higher in boys than in girls? A new hypothesis based on preconception environment and evidence from a large sample of twins. Demography. 2013;50:421–44. 10.1007/s13524-012-0161-5 23151996

[37] Maity B Comparing health outcomes across Scheduled Tribes and Castes in India. World Dev. 2017;96:163–81. 10.1016/j.worlddev.2017.03.005

[38] Bang AT Bang RA Baitule SB Reddy MH Deshmukh MD Effect of home-based neonatal care and management of sepsis on neonatal mortality: field trial in rural India. Lancet. 1999;354:1955–61. 10.1016/S0140-6736(99)03046-9 10622298

[39] Vinayak S Sande J Nisenbaum H Nolsøe CP Training Midwives to Perform Basic Obstetric Point-of-Care Ultrasound in Rural Areas Using a Tablet Platform and Mobile Phone Transmission Technology-A WFUMB COE Project. Ultrasound Med Biol. 2017;43:2125–32. 10.1016/j.ultrasmedbio.2017.05.024 28716434

[40] Rammohan A Iqbal K Awofeso N Reducing neonatal mortality in India: critical role of access to emergency obstetric care. PLoS One. 2013;8:e57244. 10.1371/journal.pone.0057244 23544038 PMC3609864

[41] Gupta S Ramadass S Ballabgarh Teaching and Research Group Kumar R Salve HR Yadav K Use of web-based health information portals in primary health care: Experience from a rural Primary Health Centre in Haryana. J Family Med Prim Care. 2021;10:3144–50. 10.4103/jfmpc.jfmpc_2352_20 34660460 PMC8483133

[42] McClure EM Garces AL Hibberd PL Moore JL Goudar SS Saleem S The Global Network Maternal Newborn Health Registry: a multi-country, community-based registry of pregnancy outcomes. Reprod Health. 2020;17:184. 10.1186/s12978-020-01020-8 33256769 PMC7708188

[43] Gao W Cao W Lv J Yu C Wu T Wang Set al. The Chinese National Twin Registry: a ‘gold mine’ for scientific research. J Intern Med. 2019;286:299–308. 10.1111/joim.12926 31270876

[44] Hopper JL Foley DL White PA Pollaers V Australian Twin Registry: 30 years of progress. Twin Res Hum Genet. 2013;16:34–42. 10.1017/thg.2012.121 23200298

[45] Skytthe A Kyvik K Holm NV Vaupel JW Christensen K The Danish Twin Registry: 127 birth cohorts of twins. Twin Res. 2002;5:352–7. 10.1375/136905202320906084 12537858

[46] Kaprio J The Finnish Twin Cohort Study: an update. Twin Res Hum Genet. 2013;16:157–62. 10.1017/thg.2012.142 23298696 PMC4493754

[47] Sumathipala A Siribaddana S Hotopf M McGuffin P Glozier N Ball H The Sri Lankan Twin Registry: 2012 Update. Twin Res Hum Genet. 2013;16:307–12. 10.1017/thg.2012.119 23302519

[48] Magnusson PK Almqvist C Rahman I Ganna A Viktorin A Walum H The Swedish Twin Registry: establishment of a biobank and other recent developments. Twin Res Hum Genet. 2013;16:317–29. 10.1017/thg.2012.104 23137839

[49] Lichtenstein P Holm NV Verkasalo PK Iliadou A Kaprio J Koskenvuo M Environmental and heritable factors in the causation of cancer–analyses of cohorts of twins from Sweden, Denmark, and Finland. N Engl J Med. 2000;343:78–85. 10.1056/NEJM200007133430201 10891514

[50] Behrman JR. Twins studies in economics. In: Komlos J, Kelly IR, editors. The Oxford handbook of economics and human biology. Oxford, UK: Oxford University Press; 2016. p. 384–404.

[51] Miller P Mulvey C Martin N What do twins studies reveal about the economic returns to education? A comparison of Australian and US findings. Am Econ Rev. 1995;85:586–99.

[52] Monden CWS Smits J Mortality among twins and singletons in sub-Saharan Africa between 1995 and 2014: a pooled analysis of data from 90 Demographic and Health Surveys in 30 countries. Lancet Glob Health. 2017;5:e673–9. 10.1016/S2214-109X(17)30197-3 28578941

[53] Neal S. The measurement of neonatal mortality: how reliable is demographic and household survey data? (ESRC Centre for Population Change Working Paper Series) Southampton, UK: University of Southampton; 2012. Available: https://eprints.soton.ac.uk/345835/. Accessed: 3 April 2025.

[54] Oza S Cousens SN Lawn JE Estimation of daily risk of neonatal death, including the day of birth, in 186 countries in 2013: a vital-registration and modelling-based study. Lancet Glob Health. 2014;2:e635–44. 10.1016/S2214-109X(14)70309-2 25442688

