# Supplementary material for: Association between risk of mortality among children and twin birth in India: an econometric analysis of live births between 1993–2021
Source: J Glob Health. 2025 May 5;15:04136. doi: 10.7189/jogh.15.04136 (PMC12051411; doi:10.7189/jogh.15.04136)
Supplement: Online Supplementary Document [file jogh-15-04136-s001.pdf]

Supplement to: William J, Prakash A, Ahluwalia K, Kim R, Subramanian SV. Association between risk of mortality among children and twin birth in India: An econometric analysis of live births between 1993–2021. J Glob Health. 2025;15:04136.

Supplementary Material

Table of Contents

Method S1: Household Wealth Index 4

Method S2: Coarsened Exact Matching (CEM) 5

Table S1: Study sample size selection from the National Family Health Survey, 1993-2021. 6

Table S2: Sample size of live birth and deaths categorized by early neonatal, late neonatal, post neonatal, child, singletons, and twins, 1993-2021 7

Table S3: Percentage distribution of live births and deaths among singletons and twins, and comparable distribution using CEM approach, 1993-2021. 8

Table S4: Odds Ratios (OR) and 95% Confidence Intervals (CI) for twin birth associated with the household wealth index on early neonatal, late neonatal, post-neonatal, and child mortality (2016-2021) based on conventional logistic regression and adjusted logistic regression with CEM approach. 9

Table S5: Adjusted Logistic regression: Odds ratios and 95% confidence intervals for the association between covariates and early neonatal mortality (1993-2021), adjusted for twin birth. 10

Table S6: Adjusted Logistic regression: Odds ratios and 95% confidence intervals for the association between covariates and late neonatal mortality (1993-2021), adjusted for twin birth. 11

Table S7: Adjusted Logistic regression: Odds ratios and 95% confidence intervals for the association between covariates and post neonatal mortality (1993-2021), adjusted for twin birth. 12

Table S8: Adjusted Logistic regression: Odds ratios and 95% confidence intervals for the association between covariates and child mortality (1993-2021), adjusted for twin birth. 13

Table S9: Percentage distribution of singleton and twin birth across covariates (1993-2021) with Coarsened Exact Matching (CEM) method. 14

Table S10: Adjusted Logistic regression with Coarsened Exact Matching (CEM) method: Odds ratios and 95% confidence intervals for the association between covariates and early neonatal mortality (1993-2021), adjusted for twin birth. 16

Table S11: Adjusted Logistic regression with Coarsened Exact Matching (CEM) method: Odds ratios and 95% confidence intervals for the association between covariates and late neonatal mortality (1993-2021), adjusted for twin birth. ... 17

Table S12: Adjusted Logistic regression with Coarsened Exact Matching (CEM) method: Odds ratios and 95% confidence intervals for the association between covariates and post neonatal mortality (1993-2021), adjusted for twin birth. ... 18

Table S13: Adjusted Logistic regression with Coarsened Exact Matching (CEM) method: Odds ratios and 95% confidence intervals for the association between covariates and child mortality (1993-2021), adjusted for twin birth. ... 19

Table S14: Adjusted Logistic regression with Coarsened Exact Matching (CEM) method: Odds ratios and 95% confidence intervals for the associations between Covariates and early neonatal mortality (2016-2021) across Household Wealth Index adjusted for twin birth ... 20

Table S15: Adjusted Logistic regression with Coarsened Exact Matching (CEM) method: Odds ratios and 95% confidence intervals for the associations between Covariates and late neonatal mortality (2016-2021) across Household Wealth Index adjusted for twin birth ... 21

Table S16: Adjusted Logistic regression with Coarsened Exact Matching (CEM) method: Odds ratios and 95% confidence intervals for the associations between Covariates and post neonatal mortality (2016-2021) across Household Wealth Index adjusted for twin birth ... 22

Table S17: Adjusted Logistic regression with Coarsened Exact Matching (CEM) method: Odds ratios and 95% confidence intervals for the associations between Covariates and child mortality (2016-2021) across Household Wealth Index adjusted for twin birth ... 23

Table S18: Adjusted Logistic regression: Odds ratios and 95% confidence intervals for the association between covariates, maternal and newborn care, and early neonatal mortality (1993-2021), adjusted for twin birth. ... 24

Table S19: Adjusted Logistic regression: Odds ratios and 95% confidence intervals for the association between covariates, maternal and newborn care, and late neonatal mortality (1993-2021), adjusted for twin birth. ... 26

Table S20: Adjusted Logistic regression: Odds ratios and 95% confidence intervals for the association between covariates, maternal and newborn care, and post neonatal mortality (1993-2021), adjusted for twin birth. ... 28

Table S21: Adjusted Logistic regression: Odds ratios and 95% confidence intervals for the association between covariates, maternal and newborn care, and child mortality (1993-2021), adjusted for twin birth. ... 30

Table S22: Adjusted Logistic regression: Odds ratios and 95% confidence intervals for the association between maternal and newborn care and early neonatal mortality (1993-2021), adjusted for twin birth. ... 32

Table S23: Adjusted Logistic regression: Odds ratios and 95% confidence intervals for the association between maternal and newborn care and late neonatal mortality (1993-2021), adjusted for twin birth. ... 33

Table S24: Adjusted Logistic regression: Odds ratios and 95% confidence intervals for the association between maternal and newborn care and post neonatal mortality (1993-2021), adjusted for twin birth. 34

Table S25: Adjusted Logistic regression: Odds ratios and 95% confidence intervals for the association between maternal and newborn care, maternal nutrition, and child mortality (1993-2021), adjusted for twin birth. 35

Table S26: Sensitivity analysis including higher birth order and missing maternal education. 36

Table S27: Adjusted logistic regression: odds ratios and 95% confidence intervals for the association between covariates and early neonatal mortality (1993–2021), adjusted for twin birth, and the interaction between maternal education and wealth index. 37

Table S28: Adjusted Logistic Regression: Odds ratios and 95% confidence intervals for the association between covariates and late neonatal mortality (1993–2021), adjusted for twin birth, and the interaction between maternal education and wealth index. 39

Table S29: Adjusted Logistic Regression: Odds ratios and 95% confidence intervals for the association between covariates and post neonatal mortality (1993–2021), adjusted for twin birth, and the interaction between maternal education and wealth index. 41

Table S30: Adjusted Logistic Regression: Odds ratios and 95% confidence intervals for the association between covariates and child mortality (1993–2021), adjusted for twin birth, and the interaction between maternal education and wealth index. 43

Table S31: Adjusted Logistic Regression: Odds ratios and 95% confidence intervals for the association between covariates and early neonatal mortality (1993–2021), adjusted for twin birth, and the interaction between maternal education and caste. 45

Table S32: Adjusted Logistic Regression: Odds ratios and 95% confidence intervals for the association between covariates and late neonatal mortality (1993–2021), adjusted for twin birth, and the interaction between maternal education and caste. 47

Table S33: Adjusted Logistic Regression: Odds ratios and 95% confidence intervals for the association between covariates and post neonatal mortality (1993–2021), adjusted for twin birth, and the interaction between maternal education and caste. 49

Table S34: Adjusted Logistic Regression: Odds ratios and 95% confidence intervals for the association between covariates and child mortality (1993–2021), adjusted for twin birth, and the interaction between maternal education and caste. 51

Method S1: Household Wealth Index

In each round of the National Family Health Survey, the household wealth index is constructed using the standard DHS wealth index [1] methodology, which employs principal component analysis (PCA) to assign weights to indicators such as household assets, utility services, and country-specific items. The process begins with the standardization of these indicators through z-scores, followed by the calculation of factor coefficient scores, or loadings. These loadings are then applied to the indicator values for each household, producing a standardized wealth index score with a mean of zero and a standard deviation of one. The wealth index is derived from the first factor generated in this analysis. To create wealth quintiles, cut points are determined through a weighted frequency distribution of households, where the weight is the product of the number of de jure household members and the household's sampling weight. Finally, households are divided into five equal sections, ensuring that each member is classified into the appropriate wealth quintile category.

Reference

DHS. Wealth Index Construction. The DHS Program https://dhsprogram.com/topics/wealth-index/Wealth-Index-Construction.cfm (2019).

Method S2: Coarsened Exact Matching (CEM)

Coarsened Exact Matching (CEM) is effective in reducing imbalance between treated and control groups by coarsening and matching on observed covariates. This makes it robust to model misspecification. However, like other matching methods, CEM does not inherently address unobserved confounders, and its effectiveness relies on the completeness and quality of the observed data (Iacus et al., 2012).

In this study, CEM was applied as a preprocessing step to balance the data. Twins were designated as the treated group, while singletons served as the control group. Matching was performed using a set of key covariates, including child age, birth order, maternal education, maternal age, household wealth index, caste, and place of residence. Each covariate was first coarsened into broader categories to facilitate the matching process. After matching, only the original, uncoarsened values were retained for subsequent analysis.

The matched sample was analyzed using logistic regression. Post-matching, weights were assigned such that each treated observation received a weight of 1, while control observations were weighted so that the sum of their weights equaled the number of treated observations within each stratum (defined by the unique combination of matching variables). The CEM process effectively minimized confounding by ensuring that treated and control groups were balanced on the coarsened covariates(Blackwell et al., 2009). This reduction in imbalance helped to minimize bias by creating comparable groups that were similar across key observed characteristics. To assess the quality of matching, balance statistics such as standardized differences and the L1 imbalance measure were reported. The L1 distances across NFHS rounds demonstrated improved balance, as shown below:

NFHS Rounds	L1 Distance
NFHS 5	1.745e-13
NFHS 4	0.926e-13
NFHS 3	0.192e-13
NFHS 2	0.024e-13
NFHS 1	0.017e-13

The robustness of the causal claims was further validated by the high-quality matching achieved through CEM. The reported balance statistics confirmed that the treated and control groups were comparable, thereby reducing the risk of confounding bias. Additionally, incorporating the original, uncoarsened matching variables into the regression model further adjusted for any residual imbalance, ensuring that the estimates were less dependent on model assumptions. This strengthened the validity of the Sample Average Treatment Effect on the Treated (SATT) and provided reliable causal inferences.

Reference

Iacus SM, King G, Porro G. Causal inference without balance checking: Coarsened exact matching. Polit Anal. 2012;20(1):1–24.

Blackwell, M., Iacus, S., King, G., & Porro, G. (2009). cem: Coarsened exact matching in Stata. The Stata Journal, 9(4), 524-546.

Table S1: Study sample size selection from the National Family Health Survey, 1993-2021.

Survey Round (year)	Sample Size based on inclusion criteria (n)	Excluding the number of children who are triplet or higher (n)	Non-responses on maternal education (n)	Final study sample size (n)
NFHS 5 (2019-21)	231033	60	0	230973
NFHS 4 (2015-16)	259627	66	0	259561
NFHS 3 (2005-06)	51555	25	1	51529
NFHS 2 (1998-99)	56734	6	27	56701
NFHS 1 (1992-93)	60625	15	199	60411
All Waves	659574	172	227	659175

Table S2: Sample size of live birth and deaths categorized by early neonatal, late neonatal, post neonatal, child, singletons, and twins, 1993-2021.

NFHS waves	Total sample	Live Birth	Deaths	Early neonatal deaths	Late neonatal deaths	Post neonatal deaths	Child death	Singleton deaths	Twin deaths
**2019-21**	230973	222320	8653	4682	935	2265	771	8024	629
**2015-16**	259561	247702	11859	6394	1276	3007	1182	10977	882
**2005-06**	51529	48662	2867	1322	384	793	368	2685	182
**1998-99**	56701	52449	4252	1668	594	1252	738	4008	244
**1992-93**	60411	55478	4933	1819	791	1529	794	4598	335
**Coarsened Exact Matching**									
**2019-21**	169427	162995	6432	3460	700	1671	601	5085	627
**2015-16**	193178	184369	8809	4712	947	2225	925	7931	878
**2005-06**	20113	18972	1141	503	157	334	147	962	179
**1998-99**	26136	23959	2177	811	311	644	411	1939	238
**1992-93**	38553	35238	3315	1207	526	1037	545	2980	335

Table S3: Percentage distribution of live births and deaths among singletons and twins, and comparable distribution using CEM approach, 1993-2021.

	2019-21		2015-16		2005-06		1998-99		1992-93	
	%	n	%	n	%	n	%	n	%	n
**Percent share in under five live births**										
Singleton	98·5	219191	98·6	244350	98·8	48064	98·9	51861	99·1	54941
Twin	1·5	3129	1·4	3352	1·2	598	1·1	588	0·9	537
**Percent share in under five deaths**										
Singleton	92·3	8024	92·7	10977	93·6	2685	94·3	4008	92·9	4598
Twin	7·7	629	7·3	822	6·4	182	5·7	244	7·2	335
**Percent share of under-five deaths**										
Early neonatal deaths	54·8	4682	55·4	6394	47·1	1322	40·5	1668	38·0	1819
Late neonatal deaths	11·2	935	10·9	1276	13·4	384	13·7	594	15·8	791
Post neonatal deaths	25·4	2265	23·8	3007	26·5	793	28·4	1252	30·1	1529
Child deaths	8·6	771	10·0	1182	13·0	368	17·4	738	16·1	794
Under five deaths	100·0	8653	100·0	11859	100·0	2867	100·0	4252	100·0	4933
**Coarsened Exact Matching**										
**Percent share in under five live births**										
Singleton	98·1	159890	98·2	181029	96·9	18387	97·6	23385	98·5	34709
Twin	1·9	3105	1·8	3340	3·1	585	2·4	574	1·5	529
**Percent share in under five deaths**										
Singleton	89·8	5805	89·8	7931	84·5	962	88·5	1939	89·9	2980
Twin	10·2	627	10·2	878	15·5	179	11·5	238	10·2	335
**Percent share of under-five death**										
Early neonatal deaths	51·0	3460	51·1	4712	40·4	503	36·2	811	38·9	1207
Late neonatal deaths	11·5	700	11·1	947	16·5	157	15·2	311	14·5	526
Post neonatal deaths	27·9	1671	27·1	2225	30·8	334	28·0	644	30·7	1037
Child deaths	9·7	601	10·7	925	12·3	147	20·6	411	16·0	545
Under five deaths	100·0	6432	100·0	8809	100·0	1141	100·0	2177	100·0	3315

Table S4: Odds Ratios (OR) and 95% Confidence Intervals (CI) for twin birth associated with the household wealth index on early neonatal, late neonatal, post-neonatal, and child mortality (2016-2021) based on conventional logistic regression and adjusted logistic regression with CEM approach.

	Early neonatal		Late neonatal		Post neonatal		Child	
	**OR**	**95 % CI**	**OR**	**95 % CI**	**OR**	**95 % CI**	**OR**	**95 % CI**
**Twin vs. Singleton(ref.)**								
Poorest [Unadjusted]	8·7	(7·49,10·11)	9·79	(7·48,12·81)	4·34	(3·40,5·54)	1·74	(0·80,3·77)
Poorest [Adjusted]	9·82	(8·43,11·44)	10·36	(7·86,13·64)	4·22	(3·31,5·39)	1·57	(0·71,3·46)
Poor [Unadjusted]	6·22	(5·20,7·43)	12·69	(9·31,17·29)	3·52	(2·58,4·81)	1·05	(0·37,2·92)
Poor [Adjusted]	6·8	(5·67,8·16)	12·99	(9·48,17·78)	3·21	(2·35,4·38)	0·98	(0·35,2·73)
Middle [Unadjusted]	5·57	(4·49,6·89)	8·71	(5·94,12·77)	3·86	(2·69,5·55)	1·17	(0·38,3·60)
Middle [Adjusted]	6·16	(4·97,7·65)	9·36	(6·33,13·84)	4·02	(2·80,5·78)	1·15	(0·38,3·49)
Rich [Unadjusted]	6·55	(5·00,8·58)	6·24	(3·63,10·72)	3·03	(1·86,4·94)	0·37	(0·05,2·69)
Rich [Adjusted]	7·32	(5·57,9·63)	6·22	(3·64,10·64)	3·04	(1·85,4·98)	0·43	(0·06,3·11)
Richest [Unadjusted]	3·25	(2·27,4·65)	3·68	(1·91,7·10)	4·03	(2·28,7·15)	0·17	(0·02,1·22)
Richest [Adjusted]	3·44	(2·41,4·92)	3·6	(1·81,7·15)	4·19	(2·35,7·50)	0·16	(0·02,1·20)
**Coarsened Exact Matching**								
Poorest [Unadjusted]	9·49	(8·42,10·70)	9·62	(7·57,12·23)	4·5	(3·62,5·60)	1·16	(0·68,1·97)
Poorest [Adjusted]	9·67	(8·57,10·92)	9·86	(7·76,12·54)	4·54	(3·66,5·64)	1·17	(0·68,1·99)
Poor [Unadjusted]	7·07	(6·09,8·20)	11·01	(8·34,14·53)	3·53	(2·69,4·63)	1·25	(0·62,2·54)
Poor [Adjusted]	7·15	(6·16,8·31)	11·12	(8·42,14·68)	3·54	(2·70,4·65)	1·26	(0·62,2·55)
Middle [Unadjusted]	6·23	(5·20,7·47)	10·06	(7·24,13·97)	3·66	(2·70,4·97)	1·26	(0·52,3·09)
Middle [Adjusted]	6·31	(5·25,7·57)	10·23	(7·35,14·23)	3·74	(2·76,5·07)	1·29	(0·53,3·16)
Rich [Unadjusted]	6·27	(5·11,7·71)	7·84	(5·13,11·99)	2·8	(1·88,4·17)	0·4	(0·06,2·87)
Rich [Adjusted]	6·36	(5·17,7·82)	7·92	(5·18,12·11)	2·86	(1·92,4·26)	0·4	(0·06,2·90)
Richest [Unadjusted]	3·83	(2·84,5·16)	5·31	(3·08,9·15)	3·69	(2·33,5·84)	0·53	(0·07,3·87)
Richest [Adjusted]	3·85	(2·86,5·19)	5·34	(3·09,9·23)	3·79	(2·41,5·95)	0·55	(0·07,4·05)

Table S5: Adjusted Logistic regression: Odds ratios and 95% confidence intervals for the association between covariates and early neonatal mortality (1993-2021), adjusted for twin birth.

	2019-21		2015-16		2005-06		1998-99		1992-93	
	OR	95 % CI	OR	95 % CI	OR	95 % CI	OR	95 % CI	OR	95 % CI
Child sex										
Boys vs. Girls (ref.)	1.24	(1.15,1.34)	1.34	(1.25,1.43)	1.3	(1.14,1.49)	1.12	(1.00,1.25)	1.29	(1.15,1.44)
Birth order										
2 to 3 vs. First (ref.)	0.69	(0.63,0.76)	0.64	(0.59,0.69)	0.57	(0.48,0.68)	0.79	(0.68,0.92)	0.61	(0.52,0.71)
4 or more vs. First (ref.)	0.88	(0.75,1.02)	0.79	(0.70,0.89)	0.8	(0.62,1.02)	0.9	(0.73,1.11)	0.62	(0.50,0.77)
Maternal age at birth										
less than 20 vs. 30 or more (ref.)	1.27	(1.08,1.49)	1.19	(1.03,1.37)	1.57	(1.16,2.13)	1.4	(1.10,1.79)	1.26	(0.99,1.60)
20 to 24 vs. 30 or more (ref.)	0.98	(0.87,1.12)	0.98	(0.88,1.10)	1.3	(1.00,1.68)	1	(0.81,1.23)	0.92	(0.75,1.14)
25 to 29 vs. 30 or more (ref.)	0.89	(0.79,1.01)	0.85	(0.76,0.94)	0.93	(0.73,1.20)	0.84	(0.69,1.02)	0.9	(0.74,1.09)
Maternal education										
No schooling vs. Above 12th grade (ref.)	1.73	(1.44,2.09)	1.68	(1.41,1.99)	2.07	(1.30,3.31)	1.25	(0.80,1.94)	1.24	(0.76,2.02)
1 to 5th vs. Above 12th grade (ref.)	1.58	(1.30,1.91)	1.76	(1.48,2.10)	2.07	(1.28,3.36)	1.04	(0.66,1.63)	1.08	(0.66,1.79)
6 to 8th vs. Above 12th grade (ref.)	1.58	(1.31,1.90)	1.56	(1.31,1.85)	1.39	(0.86,2.25)	0.99	(0.63,1.57)	0.99	(0.60,1.64)
10 to 12th vs. Above 12th grade (ref.)	1.24	(1.04,1.47)	1.11	(0.94,1.31)	1.37	(0.89,2.12)	0.92	(0.58,1.44)	0.87	(0.52,1.43)
Household (Wealth Index)										
Poorest vs. Richest (ref.)	2.16	(1.77,2.64)	1.93	(1.63,2.30)	1.32	(0.94,1.85)	1.43	(1.09,1.87)	1.91	(1.45,2.54)
Poor vs. Richest (ref.)	2.04	(1.69,2.47)	1.72	(1.45,2.03)	1.14	(0.81,1.59)	1.19	(0.91,1.56)	1.79	(1.36,2.35)
Middle vs. Richest (ref.)	1.68	(1.39,2.03)	1.54	(1.31,1.82)	1.07	(0.78,1.48)	1.39	(1.08,1.79)	1.72	(1.32,2.25)
Rich vs. Richest (ref.)	1.58	(1.30,1.91)	1.31	(1.11,1.54)	1.1	(0.82,1.48)	1.22	(0.96,1.54)	1.38	(1.06,1.78)
Caste										
SC vs. Other (ref.)	1.22	(1.07,1.40)	1.11	(0.99,1.25)	1.1	(0.90,1.34)	1.17	(0.99,1.37)	1.23	(1.06,1.44)
ST vs. Other (ref.)	1.02	(0.88,1.19)	0.92	(0.81,1.06)	0.8	(0.62,1.04)	1	(0.81,1.23)	0.93	(0.77,1.13)
OBC vs. Other (ref.)	1.1	(0.97,1.25)	1.12	(1.01,1.25)	0.98	(0.82,1.17)	1.16	(1.01,1.33)	.	.
Don't know vs. Other (ref.)	1.21	(0.82,1.79)	0.98	(0.68,1.41)	0.55	(0.12,2.40)		.	.	.
No Caste vs. Other (ref.)	0.71	(0.55,0.92)	0.78	(0.62,0.98)	0.91	(0.61,1.37)	0.85	(0.48,1.53)	.	.
Place of residence										
Rural vs. Urban (ref.)	1.1	(0.97,1.24)	1.22	(1.09,1.36)	1.14	(0.96,1.36)	1.13	(0.95,1.33)	1.03	(0.86,1.23)

OR: Odds Ratio; CI: Confidence Interval

Table S6: Adjusted Logistic regression: Odds ratios and 95% confidence intervals for the association between covariates and late neonatal mortality (1993-2021), adjusted for twin birth.

	2019-21		2015-16		2005-06		1998-99		1992-93	
	OR	95 % CI	OR	95 % CI	OR	95 % CI	OR	95 % CI	OR	95 % CI
Child sex										
Boys vs. Girls (ref.)	1.09	(0.91,1.29)	1.22	(1.05,1.42)	0.75	(0.58,0.95)	1.18	(0.98,1.43)	1.06	(0.89,1.25)
Birth order										
2 to 3 vs. First (ref.)	0.85	(0.67,1.08)	0.88	(0.71,1.08)	0.77	(0.55,1.08)	0.88	(0.68,1.13)	0.76	(0.60,0.97)
4 or more vs. First (ref.)	1.2	(0.86,1.67)	1.09	(0.82,1.43)	1.02	(0.65,1.58)	1.29	(0.92,1.81)	0.77	(0.56,1.06)
Maternal age at birth										
less than 20 vs. 30 or more (ref.)	1.51	(1.05,2.18)	1.21	(0.88,1.65)	1.11	(0.67,1.85)	2.01	(1.37,2.95)	1.07	(0.75,1.52)
20 to 24 vs. 30 or more (ref.)	1.19	(0.90,1.58)	0.89	(0.68,1.15)	1.09	(0.71,1.67)	1.21	(0.87,1.68)	0.99	(0.74,1.32)
25 to 29 vs. 30 or more (ref.)	0.87	(0.68,1.12)	0.9	(0.70,1.16)	0.73	(0.49,1.10)	0.83	(0.60,1.14)	0.75	(0.57,0.99)
Maternal education										
No schooling vs. Above 12th grade (ref.)	1.76	(1.11,2.80)	1.47	(0.95,2.29)	3.54	(0.90,14.00)	2.08	(0.75,5.80)	3.22	(1.27,8.18)
1 to 5th vs. Above 12th grade (ref.)	1.87	(1.13,3.08)	1.51	(0.94,2.44)	3.63	(0.90,14.60)	1.77	(0.63,4.96)	1.88	(0.72,4.90)
6 to 8th vs. Above 12th grade (ref.)	1.3	(0.82,2.07)	1.49	(0.96,2.33)	3.3	(0.83,13.13)	1.19	(0.41,3.47)	2	(0.76,5.24)
10 to 12th vs. Above 12th grade (ref.)	1.11	(0.72,1.71)	1.13	(0.74,1.72)	2.48	(0.65,9.40)	1.14	(0.40,3.28)	2.24	(0.88,5.67)
Household (Wealth Index)										
Poorest vs. Richest (ref.)	1.48	(0.96,2.29)	1.84	(1.23,2.73)	2.3	(1.17,4.51)	2.09	(1.29,3.39)	1.96	(1.24,3.10)
Poor vs. Richest (ref.)	1.53	(1.00,2.34)	1.48	(1.01,2.17)	2.37	(1.22,4.60)	1.78	(1.10,2.87)	2.21	(1.41,3.46)
Middle vs. Richest (ref.)	1.29	(0.82,2.01)	1.3	(0.88,1.92)	2.15	(1.14,4.07)	1.95	(1.23,3.11)	1.97	(1.28,3.05)
Rich vs. Richest (ref.)	1.13	(0.73,1.74)	1.06	(0.70,1.59)	1.43	(0.75,2.72)	1.1	(0.68,1.77)	1.38	(0.90,2.13)
Caste										
SC vs. Other (ref.)	0.98	(0.73,1.32)	1.09	(0.83,1.43)	1.13	(0.77,1.68)	1.18	(0.91,1.55)	0.91	(0.71,1.16)
ST vs. Other (ref.)	0.96	(0.69,1.32)	0.85	(0.63,1.13)	1.13	(0.73,1.76)	1.46	(1.08,1.98)	1.12	(0.86,1.46)
OBC vs. Other (ref.)	0.85	(0.65,1.11)	1.02	(0.80,1.31)	0.94	(0.66,1.33)	1.1	(0.86,1.41)	.	.
Don't know vs. Other (ref.)	1.81	(0.39,8.40)	0.78	(0.31,1.96)	1.27	(0.18,9.15)	.	.	.	.
No Caste vs. Other (ref.)	0.49	(0.28,0.86)	0.4	(0.24,0.66)	1.02	(0.56,1.85)	1.83	(0.94,3.57)	.	.
Place of residence										
Rural vs. Urban (ref.)	1.1	(0.84,1.45)	1.05	(0.83,1.33)	1.12	(0.79,1.57)	0.94	(0.71,1.24)	1.17	(0.89,1.53)

OR: Odds Ratio; CI: Confidence Interval

Table S7: Adjusted Logistic regression: Odds ratios and 95% confidence intervals for the association between covariates and post neonatal mortality (1993-2021), adjusted for twin birth.

	2019-21		2015-16		2005-06		1998-99		1992-93	
	OR	95 % CI	OR	95 % CI	OR	95 % CI	OR	95 % CI	OR	95 % CI
Child sex										
Boys vs. Girls (ref.)	1.09	(0.98,1.22)	0.9	(0.82,1.00)	0.74	(0.62,0.88)	0.89	(0.78,1.01)	0.92	(0.81,1.04)
Birth order										
2 to 3 vs. First (ref.)	1.09	(0.94,1.27)	0.98	(0.85,1.13)	0.92	(0.72,1.17)	0.93	(0.77,1.12)	0.95	(0.80,1.14)
4 or more vs. First (ref.)	1.55	(1.26,1.90)	1.49	(1.23,1.80)	1.12	(0.82,1.54)	1.25	(0.98,1.59)	1.29	(1.03,1.61)
Maternal age at birth										
Less than 20 vs. 30 or more (ref.)	1.84	(1.41,2.39)	1.34	(1.07,1.69)	1.77	(1.24,2.54)	1.66	(1.27,2.17)	1.25	(0.97,1.61)
20 to 24 vs. 30 or more (ref.)	1.43	(1.18,1.74)	0.92	(0.78,1.08)	1.17	(0.87,1.57)	1.2	(0.96,1.51)	1.09	(0.90,1.33)
25 to 29 vs. 30 or more (ref.)	1.18	(0.99,1.39)	0.89	(0.77,1.03)	1.15	(0.86,1.53)	1.08	(0.87,1.34)	0.76	(0.62,0.93)
Maternal education										
No schooling vs. Above 12th grade (ref.)	1.88	(1.41,2.51)	2.17	(1.57,3.01)	3.52	(1.56,7.94)	3.98	(1.74,9.07)	6.18	(2.39,15.96)
1 to 5th vs. Above 12th grade (ref.)	1.75	(1.27,2.39)	1.91	(1.37,2.67)	2.4	(1.06,5.48)	3.49	(1.53,7.98)	4.66	(1.79,12.11)
6 to 8th vs. Above 12th grade (ref.)	1.35	(1.01,1.82)	1.55	(1.11,2.16)	1.99	(0.88,4.52)	2.33	(1.00,5.41)	4.11	(1.57,10.78)
10 to 12th vs. Above 12th grade (ref.)	1.12	(0.85,1.47)	1.43	(1.05,1.95)	1.47	(0.67,3.24)	1.64	(0.71,3.80)	3.19	(1.22,8.35)
Household (Wealth Index)										
Poorest vs. Richest (ref.)	1.85	(1.40,2.45)	1.82	(1.40,2.38)	1.34	(0.82,2.18)	1.78	(1.26,2.50)	1.93	(1.44,2.59)
Poor vs. Richest (ref.)	1.63	(1.24,2.15)	1.74	(1.34,2.26)	1.68	(1.06,2.67)	1.47	(1.05,2.06)	1.43	(1.06,1.92)
Middle vs. Richest (ref.)	1.65	(1.25,2.19)	1.68	(1.29,2.20)	1.34	(0.86,2.09)	1.37	(0.99,1.90)	1.36	(1.02,1.82)
Rich vs. Richest (ref.)	1.54	(1.14,2.08)	1.3	(1.01,1.67)	1.1	(0.70,1.73)	1.03	(0.74,1.43)	1.21	(0.92,1.59)
Caste										
SC vs. Other (ref.)	1.1	(0.89,1.35)	1.06	(0.89,1.25)	0.95	(0.73,1.25)	1.06	(0.88,1.28)	1.17	(1.00,1.38)
ST vs. Other (ref.)	1.02	(0.82,1.27)	1.03	(0.85,1.25)	0.97	(0.71,1.33)	1.21	(0.97,1.50)	0.91	(0.75,1.11)
OBC vs. Other (ref.)	1	(0.82,1.21)	1.11	(0.95,1.28)	0.93	(0.73,1.19)	1.1	(0.93,1.30)	.	.
Don't know vs. Other (ref.)	1.53	(0.62,3.79)	0.99	(0.59,1.67)	2.18	(0.95,5.02)	.	.	.	.
No Caste vs. Other (ref.)	0.83	(0.55,1.25)	1.01	(0.71,1.44)	0.58	(0.32,1.05)	2.09	(1.34,3.27)	.	.
Place of residence										
Rural vs. Urban (ref.)	0.91	(0.77,1.07)	1.02	(0.87,1.21)	1.04	(0.81,1.33)	1.02	(0.83,1.25)	0.94	(0.78,1.12)

OR: Odds Ratio; CI: Confidence Interval

Table S8: Adjusted Logistic regression: Odds ratios and 95% confidence intervals for the association between covariates and child mortality (1993-2021), adjusted for twin birth.

	2019-21		2015-16		2005-06		1998-99		1992-93	
	OR	95 % CI	OR	95 % CI	OR	95 % CI	OR	95 % CI	OR	95 % CI
Child sex										
Boys vs. Girls (ref.)	0.98	(0.81,1.19)	0.85	(0.72,1.01)	0.61	(0.47,0.79)	0.55	(0.46,0.65)	0.61	(0.51,0.72)
Birth order										
2 to 3 vs. First (ref.)	1.34	(1.04,1.74)	0.96	(0.76,1.21)	1.34	(0.93,1.94)	1.47	(1.13,1.91)	1.25	(0.98,1.59)
4 or more vs. First (ref.)	1.88	(1.33,2.65)	1.47	(1.13,1.91)	1.75	(1.14,2.68)	2.48	(1.79,3.44)	1.75	(1.29,2.38)
Maternal age at birth										
less than 20 vs. 30 or more (ref.)	1.25	(0.84,1.85)	0.88	(0.62,1.25)	1.49	(0.92,2.41)	1.64	(1.17,2.31)	1.33	(0.93,1.89)
20 to 24 vs. 30 or more (ref.)	1.02	(0.77,1.34)	0.93	(0.73,1.17)	1.12	(0.76,1.63)	1.21	(0.92,1.60)	1.3	(0.99,1.71)
25 to 29 vs. 30 or more (ref.)	0.79	(0.61,1.03)	0.92	(0.75,1.14)	1	(0.68,1.47)	0.81	(0.63,1.05)	1.17	(0.90,1.51)
Maternal education										
No schooling vs. Above 12th grade (ref.)	2.94	(1.60,5.42)	3.03	(1.75,5.24)	3.5	(0.85,14.48)	2.44	(0.66,9.00)	4.87	(0.83,28.69)
1 to 5th vs. Above 12th grade (ref.)	1.9	(1.01,3.59)	2.72	(1.53,4.81)	2.06	(0.49,8.74)	1.62	(0.43,6.13)	2.57	(0.43,15.41)
6 to 8th vs. Above 12th grade (ref.)	1.94	(1.02,3.71)	1.81	(1.04,3.16)	1.01	(0.22,4.65)	1.48	(0.39,5.56)	2.46	(0.41,14.81)
10 to 12th vs. Above 12th grade (ref.)	1.31	(0.71,2.42)	1.39	(0.76,2.56)	0.96	(0.22,4.21)	1.23	(0.33,4.67)	1.41	(0.23,8.82)
Household (Wealth Index)										
Poorest vs. Richest (ref.)	2.27	(1.28,4.03)	2.89	(1.60,5.24)	1.76	(0.87,3.57)	4.69	(2.62,8.40)	3.06	(1.85,5.06)
Poor vs. Richest (ref.)	1.77	(1.02,3.08)	2.19	(1.25,3.85)	1.8	(0.89,3.63)	3.31	(1.86,5.90)	2.85	(1.73,4.67)
Middle vs. Richest (ref.)	1.46	(0.82,2.60)	1.93	(1.01,3.70)	1.4	(0.70,2.80)	2.63	(1.46,4.73)	2.3	(1.41,3.77)
Rich vs. Richest (ref.)	1.15	(0.63,2.09)	1.7	(1.03,2.82)	0.94	(0.45,1.96)	1.55	(0.88,2.73)	1.9	(1.17,3.10)
Caste										
SC vs. Other (ref.)	1.29	(0.89,1.89)	1.2	(0.81,1.78)	1.62	(1.07,2.46)	0.99	(0.78,1.26)	1.13	(0.90,1.42)
ST vs. Other (ref.)	1	(0.68,1.47)	1.31	(0.90,1.91)	2.2	(1.42,3.39)	1.39	(1.08,1.78)	1.25	(0.99,1.58)
OBC vs. Other (ref.)	1.12	(0.79,1.59)	1.17	(0.80,1.69)	1.08	(0.72,1.61)	0.92	(0.73,1.16)	.	.
Don't know vs. Other (ref.)	0.9	(0.16,5.16)	1.43	(0.64,3.18)	1	(1.00,1.00)	.	.	.	.
No Caste vs. Other (ref.)	0.49	(0.23,1.03)	0.69	(0.38,1.26)	1.05	(0.45,2.47)	1.87	(1.08,3.25)	.	.
Place of residence										
Rural vs. Urban (ref.)	0.8	(0.58,1.11)	0.87	(0.63,1.21)	0.95	(0.67,1.35)	0.8	(0.60,1.06)	0.96	(0.73,1.26)

OR: Odds Ratio; CI: Confidence Interval

Table S9: Percentage distribution of singleton and twin birth across covariates (1993-2021) with Coarsened Exact Matching (CEM) method.

	2019-21 (N=169427)		2015-16 (N= 193178)		2005-06 (N=20113)		1998-99 (N=26136)		1992-93 (N=38553)	
	Singleton %(n)	Twin %(n)	Singleton %(n)	Twin %(n)	Singleton %(n)	Twin %(n)	Singleton %(n)	Twin %(n)	Singleton %(n)	Twin %(n)
Child sex										
Boys	51.9 (86383)	50.6 (1886)	52.6 (99045)	51.8 (2180)	49.5 (9736)	51.1 (383)	51.8 (13241)	50.4 (428)	51.2 (19333)	53.1 (444)
Girls	48.1 (79312)	49.4 (1846)	47.4 (89915)	48.2 (2038)	50.5 (9613)	48.9 (381)	48.2 (12083)	49.6 (384)	48.8 (18356)	46.9 (420)
Child birth order										
First	34.5 (54912)	21.6 (725)	32.7 (58862)	17.4 (734)	15.7 (3842)	12.5 (113)	15.1 (3998)	12.7 (97)	19.9 (7740)	9.4 (100)
2 to 3	53.6 (88768)	58.8 (2147)	52.1 (97445)	56.6 (2309)	48.8 (9795)	47.4 (385)	45 (11503)	41.5 (352)	44 (16878)	37.3 (354)
4+	11.8 (22015)	19.6 (860)	15.1 (32653)	26.1 (1175)	35.5 (5712)	40.1 (266)	39.9 (9823)	45.7 (363)	36.2 (13071)	53.4 (410)
Maternal age at birth (in years)										
less than 20	6 (9235)	6.6 (206)	6 (10599)	6.9 (243)	7.6 (1213)	12.3 (79)	14.5 (3199)	14.6 (95)	17.7 (5945)	16.3 (121)
20 to 24	45.4 (72653)	33.6 (1181)	47.7 (86694)	36.6 (1429)	42.6 (8235)	31.2 (261)	40.6 (10205)	36 (292)	37.3 (14216)	27.4 (259)
25 to 29	33.8 (56202)	37.6 (1376)	31.4 (59669)	34.8 (1526)	29.9 (6006)	36 (259)	26.7 (7104)	26.8 (230)	26.5 (10557)	27.4 (266)
30 or more	14.8 (27605)	22.1 (969)	14.9 (31998)	21.8 (1020)	19.9 (3895)	20.5 (165)	18.2 (4816)	22.5 (195)	18.5 (6971)	28.9 (218)
Maternal education										
No Schooling	22.2 (38321)	19.3 (804)	32.8 (65073)	31.7 (1370)	63.5 (9954)	51 (319)	74.1 (17828)	60.6 (458)	73.2 (25636)	65.3 (487)
1st to 5th grade	9.6 (17311)	11.5 (480)	11.5 (23645)	11.7 (507)	6.5 (1296)	11.9 (102)	7.3 (1963)	14.5 (118)	9.5 (3878)	14.5 (148)
6th to 8th grade	15.5 (27251)	15.6 (644)	15 (29068)	15.8 (674)	6.4 (1382)	12 (97)	5.8 (1611)	9.8 (84)	6 (2528)	9.6 (89)
10th to 12th grade	36 (59695)	34.4 (1164)	29.9 (53928)	27.4 (1177)	17.3 (4560)	18.2 (174)	9.1 (2757)	12.4 (114)	8.7 (4250)	7.9 (98)
Above 12th grade	16.6 (23117)	19.3 (640)	10.9 (17246)	13.3 (490)	6.3 (2157)	6.9 (72)	3.6 (1165)	2.6 (38)	2.6 (1397)	2.7 (42)
Household (Wealth Index)										

	2019-21 (N=169427)		2015-16 (N= 193178)		2005-06 (N=20113)		1998-99 (N=26136)		1992-93 (N=38553)	
	Singleton %(n)	Twin %(n)	Singleton %(n)	Twin %(n)	Singleton %(n)	Twin %(n)	Singleton %(n)	Twin %(n)	Singleton %(n)	Twin %(n)
Poorest	26.6 (49289)	22.1 (968)	28.1 (56463)	26.1 (1139)	31.5 (4548)	21 (115)	32.6 (7064)	26.4 (175)	24.1 (7895)	22.2 (157)
Poorer	22.7 (40929)	19.9 (824)	21.9 (44985)	21.1 (873)	23.6 (3696)	22.1 (144)	24.1 (5547)	21.9 (146)	25 (8367)	26.9 (168)
Middle	17.8 (28928)	17.8 (677)	18.2 (34855)	18.6 (843)	15.6 (2882)	20.7 (149)	19.7 (5335)	20.4 (188)	20 (7216)	20.6 (171)
Richer	17 (25046)	20 (655)	16.5 (27325)	17.4 (693)	12 (2857)	19.1 (166)	12.5 (3746)	18.2 (166)	16.6 (7156)	15.6 (171)
Richest	16 (21503)	20.2 (608)	15.3 (25332)	16.8 (670)	17.4 (5366)	17 (190)	11.1 (3632)	13.1 (137)	14.3 (7055)	14.8 (197)
Caste										
Scheduled caste	21.4 (31753)	19.4 (715)	18.6 (31151)	19.9 (764)	17.6 (2753)	18.1 (121)	18.2 (4193)	25.5 (172)	9.2 (3231)	14.4 (105)
Scheduled tribe	10 (33645)	10.2 (699)	10.5 (37238)	10.8 (773)	10 (2531)	7.6 (101)	8.4 (2796)	6.4 (90)	5 (2250)	6.7 (72)
OBC	50.8 (73519)	45.1 (1467)	50.9 (85425)	45.3 (1712)	47 (7465)	43.3 (258)	32.4 (7341)	29.8 (220)		
Other	15.7 (23222)	19.9 (661)	18.5 (31716)	20.8 (811)	24.5 (6318)	27.8 (241)	40.8 (10953)	36.9 (322)	85.7 (32208)	78.9 (687)
Don't know	0.1 (127)	0.6 (23)	0 (78)	0.2 (13)						
No Caste	2.1 (3429)	4.8 (167)	1.5 (3352)	3 (145)	0.9 (282)	3.2 (43)	0.2 (41)	1.5 (8)		
Place of residence										
Urban	20.7 (25101)	30.7 (851)	23.9 (36947)	27.9 (1080)	21.4 (6707)	25.2 (288)	13.1 (4143)	19.8 (194)	18.6 (8828)	22.4 (281)
Rural	79.3 (140594)	69.3 (2881)	76.1 (152013)	72.1 (3138)	78.6 (12642)	74.8 (476)	86.9 (21181)	80.2 (618)	81.4 (28861)	77.6 (583)

Table S10: Adjusted Logistic regression with Coarsened Exact Matching (CEM) method: Odds ratios and 95% confidence intervals for the association between covariates and early neonatal mortality (1993-2021), adjusted for twin birth.

	2019-21		2015-16		2005-06		1998-99		1992-93	
	OR	95 % CI	OR	95 % CI	OR	95 % CI	OR	95 % CI	OR	95 % CI
Child sex										
Boys vs. Girls (ref.)	1.33	(1.20,1.46)	1.27	(1.16,1.38)	1.27	(0.98,1.64)	1.19	(0.94,1.51)	1.4	(1.05,1.88)
Birth order										
2 to 3 vs. First (ref.)	0.76	(0.68,0.85)	0.7	(0.62,0.78)	0.7	(0.51,0.96)	0.83	(0.60,1.15)	0.71	(0.48,1.07)
4 or more vs. First (ref.)	1.07	(0.88,1.30)	0.81	(0.68,0.97)	0.79	(0.49,1.28)	0.78	(0.48,1.26)	0.78	(0.48,1.26)
Maternal age at birth										
less than 20 vs. 30 or more (ref.)	1.35	(1.06,1.71)	1.19	(0.95,1.48)	1.53	(0.83,2.84)	1.1	(0.65,1.88)	1.51	(0.89,2.55)
20 to 24 vs. 30 or more (ref.)	1.12	(0.95,1.31)	0.98	(0.84,1.14)	1.7	(1.01,2.87)	0.92	(0.60,1.40)	0.96	(0.66,1.39)
25 to 29 vs. 30 or more (ref.)	0.97	(0.84,1.12)	0.82	(0.72,0.93)	1.06	(0.67,1.67)	0.7	(0.47,1.05)	0.74	(0.55,0.97)
Maternal education										
No schooling vs. Above 12th grade (ref.)	2.17	(1.71,2.75)	1.33	(0.97,1.84)	2.42	(1.29,4.54)	2.35	(1.02,5.42)	1.07	(0.22,5.08)
1 to 5th vs. Above 12th grade (ref.)	2.02	(1.56,2.63)	1.49	(1.07,2.09)	2.31	(1.15,4.63)	1.88	(0.78,4.51)	0.92	(0.19,4.31)
6 to 8th vs. Above 12th grade (ref.)	1.99	(1.58,2.50)	1.23	(0.91,1.66)	1.71	(0.88,3.33)	1.06	(0.44,2.53)	0.85	(0.19,3.75)
10 to 12th vs. Above 12th grade (ref.)	1.51	(1.23,1.84)	1.09	(0.83,1.44)	1.09	(0.64,1.86)	1.61	(0.61,4.24)	0.44	(0.10,2.03)
Household (Wealth Index)										
Poorest vs. Richest (ref.)	1.17	(0.88,1.57)	2.06	(1.54,2.76)	2.08	(1.08,4.03)	1.22	(0.71,2.12)	0.54	(0.17,1.73)
Poor vs. Richest (ref.)	1.23	(0.93,1.62)	1.9	(1.45,2.50)	1.67	(0.96,2.92)	1.05	(0.58,1.90)	0.45	(0.14,1.49)
Middle vs. Richest (ref.)	1.04	(0.79,1.36)	1.61	(1.26,2.06)	1.3	(0.78,2.17)	1.15	(0.66,2.00)	0.53	(0.17,1.66)
Rich vs. Richest (ref.)	1.19	(0.92,1.54)	1.29	(1.02,1.63)	1.71	(1.09,2.68)	0.93	(0.54,1.61)	0.41	(0.18,0.95)
Caste										
SC vs. Other (ref.)	1.32	(1.10,1.58)	1.16	(1.00,1.36)	0.86	(0.56,1.33)	1.13	(0.86,1.48)	1.43	(0.94,2.17)
ST vs. Other (ref.)	0.84	(0.70,1.01)	0.78	(0.66,0.91)	0.55	(0.34,0.91)	1.05	(0.58,1.90)	1.17	(0.74,1.86)
OBC vs. Other (ref.)	1.2	(1.02,1.42)	1.16	(1.01,1.33)	1.11	(0.80,1.54)	1.31	(1.05,1.65)	.	.
Don't know vs. Other (ref.)	0.52	(0.24,1.13)	0.57	(0.22,1.49)	.	.	.	.	.	.
No Caste vs. Other (ref.)	0.71	(0.49,1.03)	0.86	(0.64,1.15)	0.71	(0.33,1.53)	2.21	(0.74,6.65)	.	.
Place of residence										
Rural vs. Urban (ref.)	1.41	(1.17,1.71)	1.01	(0.89,1.15)	1.23	(0.90,1.68)	1.17	(0.79,1.73)	2.02	(0.85,4.81)

OR: Odds Ratio; CI: Confidence Interval

Table S11: Adjusted Logistic regression with Coarsened Exact Matching (CEM) method: Odds ratios and 95% confidence intervals for the association between covariates and late neonatal mortality (1993-2021), adjusted for twin birth.

	2019-21		2015-16		2005-06		1998-99		1992-93	
	OR	95 % CI	OR	95 % CI	OR	95 % CI	OR	95 % CI	OR	95 % CI
Child sex										
Boys vs. Girls (ref.)	1.23	(0.93,1.62)	1.05	(0.87,1.26)	0.88	(0.49,1.61)	1.26	(0.86,1.87)	1.15	(0.91,1.45)
Birth order										
2 to 3 vs. First (ref.)	0.81	(0.56,1.18)	1	(0.79,1.25)	0.87	(0.37,2.02)	0.56	(0.21,1.52)	0.77	(0.48,1.24)
4 or more vs. First (ref.)	1.25	(0.66,2.36)	1.78	(1.20,2.64)	2.23	(0.60,8.31)	0.55	(0.17,1.77)	0.87	(0.48,1.59)
Maternal age at birth										
less than 20 vs. 30 or more (ref.)	1.42	(0.78,2.61)	2.97	(1.94,4.53)	2.6	(0.73,9.28)	1.08	(0.33,3.47)	1.24	(0.75,2.05)
20 to 24 vs. 30 or more (ref.)	1.2	(0.75,1.91)	1.46	(1.06,2.01)	1.84	(0.82,4.14)	0.86	(0.48,1.55)	1.11	(0.78,1.57)
25 to 29 vs. 30 or more (ref.)	0.88	(0.62,1.25)	1.28	(0.98,1.66)	0.94	(0.48,1.82)	0.72	(0.44,1.20)	0.86	(0.61,1.22)
Maternal education										
No schooling vs. Above 12th grade (ref.)	1.9	(1.12,3.22)	1.17	(0.72,1.89)	8.21	(0.88,76.78)	4.66	(0.50,43.17)	1.63	(0.55,4.89)
1 to 5th vs. Above 12th grade (ref.)	2.36	(1.38,4.01)	1.14	(0.70,1.87)	9.42	(0.82,107.79)	4.43	(0.47,41.87)	1.01	(0.33,3.12)
6 to 8th vs. Above 12th grade (ref.)	1.55	(0.98,2.45)	1.46	(0.92,2.30)	14.89	(1.69,131.03)	0.95	(0.11,8.28)	0.89	(0.27,2.98)
10 to 12th vs. Above 12th grade (ref.)	1.14	(0.73,1.76)	1.31	(0.85,2.01)	14.05	(1.72,115.07)	4.16	(0.69,25.02)	2.31	(0.77,6.96)
Household (Wealth Index)										
Poorest vs. Richest (ref.)	1.93	(1.03,3.61)	2.87	(1.83,4.51)	5.13	(1.89,13.94)	1.72	(0.27,10.85)	2.19	(1.08,4.42)
Poor vs. Richest (ref.)	2.43	(1.10,5.34)	2.04	(1.29,3.24)	5.04	(2.15,11.83)	1.66	(0.23,11.72)	2.62	(1.31,5.26)
Middle vs. Richest (ref.)	1.63	(0.91,2.92)	1.69	(1.14,2.51)	5.67	(2.12,15.13)	1.57	(0.28,8.70)	2.46	(1.17,5.17)
Rich vs. Richest (ref.)	1.15	(0.70,1.91)	1.3	(0.87,1.94)	1.62	(0.64,4.10)	0.64	(0.15,2.83)	1.3	(0.66,2.55)
Caste										
SC vs. Other (ref.)	0.66	(0.38,1.14)	1.15	(0.84,1.57)	1.57	(0.60,4.10)	0.98	(0.65,1.48)	0.79	(0.55,1.12)
ST vs. Other (ref.)	0.44	(0.26,0.77)	0.62	(0.46,0.83)	0.86	(0.35,2.12)	1.11	(0.64,1.93)	0.75	(0.40,1.38)
OBC vs. Other (ref.)	0.51	(0.31,0.82)	1.04	(0.80,1.34)	1.4	(0.67,2.93)	1.34	(0.81,2.21)	.	.
No Caste vs. Other (ref.)	0.32	(0.14,0.73)	0.25	(0.10,0.59)	1.57	(0.51,4.80)	1.32	(0.17,10.37)	.	.
Place of residence										
Rural vs. Urban (ref.)	0.84	(0.46,1.56)	0.81	(0.58,1.13)	0.59	(0.28,1.27)	0.67	(0.36,1.27)	1.27	(0.84,1.93)

OR: Odds Ratio; CI: Confidence Interval

Table S12: Adjusted Logistic regression with Coarsened Exact Matching (CEM) method: Odds ratios and 95% confidence intervals for the association between covariates and post neonatal mortality (1993-2021), adjusted for twin birth.

	2019-21		2015-16		2005-06		1998-99		1992-93	
	OR	95 % CI	OR	95 % CI	OR	95 % CI	OR	95 % CI	OR	95 % CI
Child sex										
Boys vs. Girls (ref.)	1.01	(0.85,1.20)	0.89	(0.79,1.01)	0.93	(0.67,1.28)	0.99	(0.78,1.26)	0.77	(0.64,0.94)
Birth order										
2 to 3 vs. First (ref.)	0.97	(0.64,1.46)	1.01	(0.83,1.23)	1.06	(0.64,1.76)	1.2	(0.82,1.75)	0.81	(0.60,1.11)
4 or more vs. First (ref.)	1.47	(0.86,2.53)	1.86	(1.37,2.52)	1.93	(0.99,3.74)	1.67	(0.99,2.82)	1.03	(0.70,1.52)
Maternal age at birth										
less than 20 vs. 30 or more (ref.)	1.52	(0.87,2.64)	1.49	(1.05,2.11)	2.61	(1.24,5.49)	1.71	(1.06,2.75)	1.29	(0.89,1.88)
20 to 24 vs. 30 or more (ref.)	1.38	(1.04,1.83)	1.04	(0.83,1.30)	1.97	(1.06,3.69)	1.62	(1.11,2.39)	1.06	(0.82,1.38)
25 to 29 vs. 30 or more (ref.)	1.19	(0.95,1.49)	0.88	(0.75,1.04)	1.36	(0.83,2.22)	1.26	(0.91,1.75)	0.9	(0.68,1.19)
Maternal education										
No schooling vs. Above 12th grade (ref.)	1.48	(0.98,2.24)	2.17	(1.22,3.84)	6.21	(1.90,20.31)	6.43	(1.97,20.98)	5.68	(1.51,21.36)
1 to 5th vs. Above 12th grade (ref.)	1.53	(1.01,2.33)	1.79	(1.01,3.15)	7.81	(2.12,28.70)	6.19	(1.84,20.81)	3.72	(0.99,13.90)
6 to 8th vs. Above 12th grade (ref.)	1.43	(0.86,2.37)	1.63	(0.92,2.88)	2.82	(0.83,9.57)	5.33	(1.69,16.77)	4.68	(1.24,17.72)
10 to 12th vs. Above 12th grade (ref.)	1.14	(0.77,1.68)	1.51	(0.90,2.53)	1.69	(0.57,4.95)	3	(0.71,12.70)	1.21	(0.32,4.58)
Household (Wealth Index)										
Poorest vs. Richest (ref.)	3.29	(1.73,6.25)	1.12	(0.79,1.59)	1.58	(0.65,3.82)	1.81	(0.80,4.09)	1.68	(1.09,2.60)
Poor vs. Richest (ref.)	2.68	(1.59,4.49)	1.11	(0.80,1.55)	2.26	(0.85,6.02)	1.66	(0.74,3.71)	1.21	(0.77,1.89)
Middle vs. Richest (ref.)	2.11	(1.31,3.39)	1.31	(0.94,1.83)	1.64	(0.66,4.12)	1.53	(0.69,3.42)	1.23	(0.77,1.97)
Rich vs. Richest (ref.)	1.61	(1.12,2.32)	1.28	(0.89,1.84)	1.19	(0.50,2.84)	1.17	(0.54,2.53)	1.09	(0.75,1.59)
Caste										
SC vs. Other (ref.)	1.25	(0.97,1.61)	1.08	(0.83,1.42)	1.22	(0.67,2.19)	1.11	(0.79,1.56)	1.08	(0.85,1.39)
ST vs. Other (ref.)	1.14	(0.88,1.47)	1.14	(0.91,1.42)	1.62	(0.88,2.98)	1.43	(0.89,2.28)	1.15	(0.72,1.84)
OBC vs. Other (ref.)	1.21	(0.96,1.53)	0.97	(0.79,1.19)	1.32	(0.86,2.02)	1.03	(0.77,1.36)	.	.
Don't know vs. Other (ref.)			1.29	(0.17,9.71)						
No Caste vs. Other (ref.)	1.57	(0.63,3.92)	0.67	(0.45,1.02)	0.55	(0.17,1.83)	0.8	(0.15,4.21)	.	.
Place of residence										
Rural vs. Urban (ref.)	0.75	(0.43,1.30)	1.08	(0.86,1.34)	0.6	(0.34,1.06)	0.89	(0.54,1.47)	0.93	(0.66,1.32)

OR: Odds Ratio; CI: Confidence Interval

Table S13: Adjusted Logistic regression with Coarsened Exact Matching (CEM) method: Odds ratios and 95% confidence intervals for the association between covariates and child mortality (1993-2021), adjusted for twin birth.

	2019-21		2015-16		2005-06		1998-99		1992-93	
	OR	95 % CI	OR	95 % CI	OR	95 % CI	OR	95 % CI	OR	95 % CI
Child sex										
Boys vs. Girls (ref.)	0.88	(0.67,1.15)	0.8	(0.68,0.94)	0.42	(0.19,0.95)	0.81	(0.57,1.16)	0.51	(0.37,0.72)
Birth order										
2 to 3 vs. First (ref.)	1.36	(0.98,1.87)	1.13	(0.88,1.46)	1.82	(0.84,3.94)	1.11	(0.36,3.48)	1.26	(0.84,1.91)
4 or more vs. First (ref.)	1.52	(0.94,2.44)	1.74	(1.24,2.43)	4.24	(1.50,12.00)	2.38	(0.58,9.88)	2.45	(1.10,5.47)
Maternal age at birth										
less than 20 vs. 30 or more (ref.)	0.84	(0.38,1.86)	1.03	(0.64,1.67)	1.98	(0.58,6.68)	2.37	(0.80,7.00)	1.95	(0.96,3.95)
20 to 24 vs. 30 or more (ref.)	1.02	(0.71,1.47)	1.05	(0.79,1.39)	1.92	(0.67,5.56)	1.41	(0.82,2.41)	2.85	(1.55,5.25)
25 to 29 vs. 30 or more (ref.)	1.21	(0.81,1.80)	1.01	(0.82,1.24)	1.28	(0.71,2.30)	1.1	(0.65,1.86)	1.05	(0.75,1.49)
Maternal education										
No schooling vs. Above 12th grade (ref.)	4.3	(2.07,8.91)	3.48	(1.69,7.15)	3.05	(0.69,13.55)	2.04	(0.23,18.24)	3.71	(0.57,23.96)
1 to 5th vs. Above 12th grade (ref.)	3.1	(1.54,6.22)	2.24	(1.06,4.70)	0.34	(0.04,2.60)	1.25	(0.09,18.34)	2.71	(0.49,15.03)
6 to 8th vs. Above 12th grade (ref.)	2.81	(1.36,5.83)	1.87	(0.86,4.05)	0.42	(0.05,3.45)	2.15	(0.24,19.42)	4.24	(0.87,20.65)
10 to 12th vs. Above 12th grade (ref.)	1.79	(0.98,3.29)	1.14	(0.56,2.34)	0.59	(0.10,3.43)	1.84	(0.27,12.70)	0.93	(0.19,4.62)
Household (Wealth Index)										
Poorest vs. Richest (ref.)	3.88	(1.63,9.24)	2.1	(1.04,4.23)	4.41	(1.63,11.89)	2.44	(0.29,20.43)	3.92	(1.13,13.53)
Poor vs. Richest (ref.)	2.62	(1.26,5.41)	1.59	(0.79,3.21)	6.64	(2.25,19.57)	1.62	(0.20,13.52)	2.69	(0.89,8.16)
Middle vs. Richest (ref.)	2.03	(1.04,3.97)	1.6	(0.77,3.32)	3.83	(1.42,10.31)	2.18	(0.26,18.41)	1.59	(0.58,4.34)
Rich vs. Richest (ref.)	2.19	(0.97,4.95)	1.48	(0.75,2.93)	6.99	(1.97,24.85)	0.84	(0.15,4.76)	0.81	(0.32,2.02)
Caste										
SC vs. Other (ref.)	0.97	(0.52,1.80)	1.19	(0.80,1.75)	7.34	(2.47,21.77)	0.87	(0.56,1.33)	1.21	(0.81,1.81)
ST vs. Other (ref.)	0.87	(0.50,1.52)	1.32	(0.92,1.89)	16.53	(4.34,63.02)	1.88	(1.03,3.42)	1.63	(0.78,3.38)
OBC vs. Other (ref.)	0.82	(0.44,1.52)	1.2	(0.84,1.73)	4.35	(1.76,10.75)	1.23	(0.72,2.10)	.	.
No Caste vs. Other (ref.)	1.66	(0.54,5.12)	0.86	(0.45,1.61)	0.57	(0.07,4.80)	.	.	.	.
Place of residence										
Rural vs. Urban (ref.)	0.57	(0.26,1.25)	1.34	(0.90,2.00)	0.29	(0.12,0.70)	0.93	(0.38,2.26)	0.98	(0.57,1.71)

OR: Odds Ratio; CI: Confidence Interval

Table S14: Adjusted Logistic regression with Coarsened Exact Matching (CEM) method: Odds ratios and 95% confidence intervals for the associations between Covariates and early neonatal mortality (2016-2021) across Household Wealth Index adjusted for twin birth

	Poorest		Poor		Middle		Rich		Richest	
	OR	95 % CI	OR	95 % CI	OR	95 % CI	OR	95 % CI	OR	95 % CI
Year										
2021 vs. 2016 (ref.)	0.85	(0.78,0.93)	0.90	(0.81,1.00)	0.86	(0.76,0.98)	1.00	(0.85,1.19)	0.86	(0.70,1.06)
Child sex										
Boys vs. Girls (ref.)	1.33	(1.22,1.45)	1.35	(1.22,1.49)	1.28	(1.13,1.46)	1.16	(0.98,1.37)	1.24	(1.01,1.53)
Birth order										
2 to 3 vs. First (ref.)	0.67	(0.60,0.76)	0.61	(0.53,0.69)	0.63	(0.54,0.73)	0.76	(0.64,0.92)	0.81	(0.65,1.01)
4 or more vs. First (ref.)	0.69	(0.59,0.81)	0.74	(0.62,0.89)	0.94	(0.74,1.21)	1.42	(1.01,1.99)	1.72	(0.98,3.03)
Maternal age at birth										
less than 20 vs. 30 or more (ref.)	1.37	(1.13,1.66)	1.12	(0.88,1.43)	1.09	(0.79,1.50)	0.91	(0.57,1.46)	0.55	(0.23,1.34)
20 to 24 vs. 30 or more (ref.)	0.97	(0.83,1.12)	0.98	(0.82,1.17)	0.83	(0.66,1.05)	1.21	(0.92,1.60)	0.95	(0.70,1.28)
25 to 29 vs. 30 or more (ref.)	0.81	(0.71,0.93)	0.88	(0.74,1.04)	0.73	(0.58,0.92)	0.98	(0.75,1.27)	1.01	(0.75,1.37)
Maternal education										
No schooling vs. Above 12th grade (ref.)	1.20	(0.62,2.33)	1.36	(1.02,1.82)	1.41	(1.07,1.85)	1.93	(1.33,2.80)	2.40	(1.38,4.16)
1 to 5th vs. Above 12th grade (ref.)	1.16	(0.60,2.26)	1.38	(1.02,1.86)	1.53	(1.14,2.05)	1.53	(1.05,2.21)	2.36	(1.38,4.03)
6 to 8th vs. Above 12th grade (ref.)	1.00	(0.52,1.95)	1.35	(1.01,1.81)	1.21	(0.92,1.58)	1.52	(1.12,2.06)	1.98	(1.36,2.88)
10 to 12th vs. Above 12th grade (ref.)	0.90	(0.46,1.76)	0.94	(0.70,1.25)	0.89	(0.70,1.15)	1.15	(0.86,1.53)	1.58	(1.25,2.00)
Caste										
SC vs. Other (ref.)	1.22	(0.99,1.51)	1.15	(0.92,1.44)	1.09	(0.87,1.36)	1.33	(1.01,1.76)	1.32	(0.97,1.80)
ST vs. Other (ref.)	0.95	(0.77,1.18)	1.11	(0.88,1.41)	0.92	(0.69,1.24)	0.69	(0.42,1.15)	1.99	(0.89,4.47)
OBC vs. Other (ref.)	1.16	(0.94,1.42)	1.14	(0.92,1.41)	1.05	(0.87,1.26)	1.22	(0.97,1.55)	1.13	(0.90,1.41)
Don't know vs. Other (ref.)	1.31	(0.60,2.86)	2.92	(1.21,7.02)	0.61	(0.15,2.53)			1.00	(1.00,1.00)
No Caste vs. Other (ref.)	0.74	(0.50,1.10)	0.81	(0.56,1.19)	0.93	(0.56,1.54)	0.64	(0.29,1.40)	0.83	(0.27,2.56)
Place of residence										
Rural vs. Urban (ref.)	1.19	(0.83,1.69)	1.03	(0.75,1.41)	1.14	(0.95,1.36)	1.10	(0.91,1.34)	1.28	(1.04,1.57)

OR: Odds Ratio; CI: Confidence Interval

Table S15: Adjusted Logistic regression with Coarsened Exact Matching (CEM) method: Odds ratios and 95% confidence intervals for the associations between Covariates and late neonatal mortality (2016-2021) across Household Wealth Index adjusted for twin birth

	Poorest		Poor		Middle		Rich		Richest	
	OR	95 % CI	OR	95 % CI	OR	95 % CI	OR	95 % CI	OR	95 % CI
Year										
2021 vs. 2016 (ref.)	0.87	(0.72,1.05)	0.96	(0.76,1.20)	0.94	(0.69,1.28)	0.96	(0.67,1.40)	0.71	(0.43,1.19)
Child sex										
Boys vs. Girls (ref.)	1.14	(0.95,1.36)	1.23	(0.98,1.53)	1.20	(0.90,1.62)	0.96	(0.66,1.40)	1.17	(0.71,1.92)
Birth order										
2 to 3 vs. First (ref.)	0.63	(0.48,0.83)	0.66	(0.50,0.88)	0.89	(0.60,1.30)	1.10	(0.69,1.75)	1.85	(1.08,3.19)
4 or more vs. First (ref.)	0.77	(0.55,1.08)	0.97	(0.65,1.46)	1.06	(0.61,1.86)	3.12	(1.36,7.18)	1.00	(0.29,3.45)
Maternal age at birth										
less than 20 vs. 30 or more (ref.)	1.12	(0.74,1.70)	1.26	(0.79,2.03)	0.97	(0.50,1.90)	2.10	(0.72,6.15)	0.48	(0.06,3.72)
20 to 24 vs. 30 or more (ref.)	0.89	(0.66,1.19)	0.80	(0.56,1.15)	1.08	(0.66,1.76)	3.06	(1.57,5.98)	1.01	(0.51,1.98)
25 to 29 vs. 30 or more (ref.)	0.99	(0.75,1.29)	0.75	(0.54,1.05)	0.89	(0.54,1.45)	2.34	(1.24,4.43)	0.59	(0.28,1.25)
Maternal education										
No schooling vs. Above 12th grade (ref.)	1.43	(1.00,2.04)	2.37	(1.08,5.17)	0.95	(0.55,1.64)	2.90	(1.26,6.66)	1.14	(0.31,4.12)
1 to 5th vs. Above 12th grade (ref.)	1.36	(0.91,2.04)	1.43	(0.65,3.18)	1.53	(0.78,3.02)	2.76	(1.42,5.39)	1.10	(0.37,3.26)
6 to 8th vs. Above 12th grade (ref.)	1.44	(0.96,2.15)	1.78	(0.81,3.91)	0.92	(0.54,1.58)	1.44	(0.78,2.67)	1.11	(0.51,2.40)
10 to 12th vs. Above 12th grade (ref.)	1.00	(1.00,1.00)	1.82	(0.83,3.99)	0.68	(0.41,1.13)	1.68	(0.95,2.98)	0.82	(0.49,1.38)
Caste										
SC vs. Other (ref.)	0.98	(0.65,1.49)	1.14	(0.76,1.70)	0.74	(0.43,1.27)	1.14	(0.62,2.12)	0.97	(0.47,1.99)
ST vs. Other (ref.)	0.88	(0.58,1.35)	0.79	(0.50,1.25)	1.00	(0.53,1.91)	1.69	(0.61,4.70)	0.93	(0.25,3.38)
OBC vs. Other (ref.)	0.81	(0.54,1.23)	1.12	(0.77,1.62)	0.86	(0.53,1.40)	1.10	(0.70,1.73)	0.55	(0.33,0.92)
Don't know vs. Other (ref.)	0.27	(0.04,2.04)	1.00	(1.00,1.00)	1.00	(1.00,1.00)			1.00	(1.00,1.00)
No Caste vs. Other (ref.)	0.32	(0.16,0.63)	0.64	(0.29,1.41)	0.07	(0.01,0.41)	0.13	(0.02,0.73)	1.36	(0.24,7.79)
Place of residence										
Rural vs. Urban (ref.)	0.77	(0.43,1.38)	1.54	(0.85,2.78)	1.03	(0.65,1.62)	1.24	(0.85,1.82)	1.36	(0.87,2.14)

OR: Odds Ratio; CI: Confidence Interval

Table S16: Adjusted Logistic regression with Coarsened Exact Matching (CEM) method: Odds ratios and 95% confidence intervals for the associations between Covariates and post neonatal mortality (2016-2021) across Household Wealth Index adjusted for twin birth

	Poorest		Poor		Middle		Rich		Richest	
	OR	95 % CI	OR	95 % CI	OR	95 % CI	OR	95 % CI	OR	95 % CI
Year										
2021 vs. 2016 (ref.)	0.97	(0.86,1.10)	0.9	(0.77,1.05)	0.88	(0.72,1.09)	1.34	(1.03,1.74)	0.94	(0.68,1.29)
Child sex										
Boys vs. Girls (ref.)	0.96	(0.85,1.08)	0.9	(0.79,1.06)	1.02	(0.83,1.26)	1.08	(0.83,1.39)	0.95	(0.70,1.31)
Birth order										
2 to 3 vs. First (ref.)	0.82	(0.68,0.97)	1.2	(0.94,1.41)	0.96	(0.72,1.28)	1.15	(0.85,1.56)	1.15	(0.83,1.59)
4 or more vs. First (ref.)	1.06	(0.84,1.34)	1.6	(1.20,2.10)	1.87	(1.26,2.76)	2.10	(1.28,3.46)	1.46	(0.71,3.00)
Maternal age at birth										
less than 20 vs. 30 or more (ref.)	1.02	(0.77,1.34)	1.7	(1.20,2.48)	1.96	(1.16,3.34)	2.03	(1.07,3.85)	0.52	(0.13,2.08)
20 to 24 vs. 30 or more (ref.)	0.89	(0.73,1.09)	1.0	(0.79,1.36)	1.45	(1.03,2.04)	1.36	(0.92,2.02)	1.68	(1.01,2.77)
25 to 29 vs. 30 or more (ref.)	0.93	(0.79,1.10)	1.0	(0.78,1.29)	1.15	(0.84,1.56)	1.23	(0.86,1.74)	1.28	(0.78,2.10)
Maternal education										
No schooling vs. Above 12th grade (ref.)	3.73	(0.91,15.29)	1.3	(0.73,2.28)	1.27	(0.81,2.00)	2.93	(1.69,5.08)	2.18	(1.07,4.45)
1 to 5th vs. Above 12th grade (ref.)	3.44	(0.84,14.19)	1.1	(0.62,1.95)	0.93	(0.58,1.50)	2.54	(1.21,5.31)	2.27	(1.05,4.92)
6 to 8th vs. Above 12th grade (ref.)	2.48	(0.60,10.24)	1.0	(0.54,1.70)	0.83	(0.50,1.37)	1.79	(1.15,2.77)	1.60	(0.92,2.77)
10 to 12th vs. Above 12th grade (ref.)	2.79	(0.67,11.58)	0.7	(0.42,1.30)	0.78	(0.52,1.18)	1.43	(0.97,2.11)	1.51	(1.03,2.21)
Caste										
SC vs. Other (ref.)	1.07	(0.81,1.42)	1.1	(0.81,1.37)	1.37	(0.99,1.91)	0.89	(0.58,1.37)	1.26	(0.74,2.15)
ST vs. Other (ref.)	1.04	(0.78,1.37)	1.1	(0.82,1.46)	1.23	(0.81,1.88)	0.78	(0.42,1.44)	1.29	(0.53,3.17)
OBC vs. Other (ref.)	1.17	(0.89,1.53)	1.0	(0.74,1.21)	1.25	(0.94,1.67)	0.86	(0.60,1.23)	1.01	(0.73,1.41)
Don't know vs. Other (ref.)	0.73	(0.22,2.37)	1.0	(1.00,1.00)	0.69	(0.09,5.58)			1.00	(1.00,1.00)
No Caste vs. Other (ref.)	1.00	(0.61,1.63)	0.8	(0.40,1.44)	1.25	(0.53,2.94)	0.27	(0.10,0.75)	2.35	(0.43,12.71)
Place of residence										
Rural vs. Urban (ref.)	1.08	(0.64,1.82)	1.3	(0.90,1.83)	0.70	(0.53,0.94)	0.90	(0.69,1.17)	0.95	(0.70,1.30)

OR: Odds Ratio; CI: Confidence Interval

Table S17: Adjusted Logistic regression with Coarsened Exact Matching (CEM) method: Odds ratios and 95% confidence intervals for the associations between Covariates and child mortality (2016-2021) across Household Wealth Index adjusted for twin birth

	Poorest		Poor		Middle		Rich		Richest	
	OR	95 % CI	OR	95 % CI	OR	95 % CI	OR	95 % CI	OR	95 % CI
Year										
2021 vs. 2016 (ref.)	0.81	(0.68,0.97)	0.89	(0.69,1.15)	0.84	(0.58,1.22)	0.71	(0.44,1.14)	0.82	(0.43,1.58)
Child sex										
Boys vs. Girls (ref.)	0.78	(0.66,0.92)	0.72	(0.55,0.92)	0.92	(0.65,1.32)	0.99	(0.61,1.61)	0.90	(0.46,1.74)
Birth order										
2 to 3 vs. First (ref.)	0.98	(0.74,1.30)	1.59	(1.15,2.20)	1.09	(0.69,1.73)	1.06	(0.55,2.02)	1.04	(0.58,1.88)
4 or more vs. First (ref.)	1.33	(0.96,1.84)	1.71	(1.07,2.72)	1.99	(1.00,3.95)	1.51	(0.53,4.30)	3.93	(1.00,15.43)
Maternal age at birth										
less than 20 vs. 30 or more (ref.)	0.86	(0.57,1.29)	1.25	(0.67,2.32)	0.95	(0.43,2.09)	1.10	(0.35,3.46)	4.89	(0.83,28.78)
20 to 24 vs. 30 or more (ref.)	0.87	(0.67,1.13)	0.94	(0.61,1.46)	0.76	(0.43,1.34)	1.07	(0.58,1.99)	1.87	(0.60,5.84)
25 to 29 vs. 30 or more (ref.)	0.84	(0.67,1.05)	0.84	(0.56,1.26)	0.95	(0.56,1.61)	0.88	(0.46,1.65)	1.17	(0.41,3.28)
Maternal education										
No schooling vs. Above 12th grade (ref.)	2.60	(1.74,3.86)	5.85	(1.81,18.94)	1.76	(0.80,3.84)	4.82	(1.69,13.69)	3.40	(0.69,16.62)
1 to 5th vs. Above 12th grade (ref.)	1.60	(1.02,2.51)	6.84	(2.09,22.42)	0.96	(0.41,2.24)	4.96	(1.66,14.77)	2.37	(0.70,8.12)
6 to 8th vs. Above 12th grade (ref.)	1.79	(1.14,2.80)	3.60	(1.08,12.01)	1.18	(0.51,2.71)	1.35	(0.63,2.89)	1.05	(0.36,3.05)
10 to 12th vs. Above 12th grade (ref.)	1.00	(1.00,1.00)	3.24	(0.99,10.63)	0.78	(0.37,1.65)	1.57	(0.78,3.14)	1.13	(0.49,2.62)
Caste										
SC vs. Other (ref.)	1.10	(0.72,1.67)	1.06	(0.66,1.68)	1.90	(0.94,3.82)	2.15	(0.97,4.75)	2.28	(0.76,6.82)
ST vs. Other (ref.)	1.06	(0.70,1.60)	1.04	(0.64,1.68)	1.99	(0.96,4.14)	1.69	(0.57,5.03)	1.00	(1.00,1.00)
OBC vs. Other (ref.)	1.03	(0.68,1.55)	0.94	(0.62,1.42)	2.90	(1.58,5.33)	1.26	(0.72,2.21)	1.15	(0.53,2.48)
Don't know vs. Other (ref.)	0.26	(0.03,1.92)	1.00	(1.00,1.00)	1.00	(1.00,1.00)			1.00	(1.00,1.00)
No Caste vs. Other (ref.)	0.64	(0.30,1.40)	0.57	(0.21,1.56)	0.29	(0.04,2.23)	0.14	(0.02,1.08)	0.31	(0.07,1.43)
Place of residence										
Rural vs. Urban (ref.)	1.16	(0.58,2.32)	0.67	(0.39,1.17)	1.08	(0.64,1.83)	0.94	(0.57,1.56)	1.18	(0.62,2.24)

OR: Odds Ratio; CI: Confidence Interval

Table S18: Adjusted Logistic regression: Odds ratios and 95% confidence intervals for the association between covariates, maternal and newborn care, and early neonatal mortality (1993-2021), adjusted for twin birth.

	2019-21		2015-16		2005-06		1998-99		1992-93	
	OR	95 % CI	OR	95 % CI	OR	95 % CI	OR	95 % CI	OR	95 % CI
Child sex										
Boys vs. Girls (ref.)	1.33	(1.23,1.44)	1.44	(1.34,1.54)	1.35	(1.18,1.55)	1.13	(1.01,1.27)	1.33	(1.18,1.49)
Birth order										
2 to 3 vs. First (ref.)	0.82	(0.75,0.90)	0.78	(0.72,0.85)	0.69	(0.57,0.82)	0.79	(0.68,0.92)	0.61	(0.52,0.71)
4 or more vs. First (ref.)	1.03	(0.88,1.21)	0.96	(0.85,1.08)	0.96	(0.73,1.24)	0.87	(0.70,1.08)	0.61	(0.49,0.75)
Maternal age at birth										
less than 20 vs. 30 or more (ref.)	1.06	(0.90,1.26)	1.07	(0.92,1.24)	1.32	(0.98,1.78)	1.39	(1.09,1.77)	1.2	(0.94,1.53)
20 to 24 vs. 30 or more (ref.)	0.86	(0.75,0.98)	0.92	(0.82,1.03)	1.15	(0.89,1.49)	1	(0.81,1.23)	0.91	(0.74,1.13)
25 to 29 vs. 30 or more (ref.)	0.85	(0.75,0.96)	0.85	(0.77,0.95)	0.88	(0.68,1.13)	0.85	(0.70,1.04)	0.9	(0.74,1.09)
Maternal education										
No schooling vs. Above 12th grade (ref.)	1.38	(1.14,1.67)	1.26	(1.06,1.50)	1.9	(1.19,3.03)	1.13	(0.72,1.78)	1.05	(0.64,1.73)
1 to 5th vs. Above 12th grade (ref.)	1.38	(1.13,1.68)	1.44	(1.20,1.72)	2.01	(1.24,3.24)	0.99	(0.63,1.57)	0.95	(0.57,1.58)
6 to 8th vs. Above 12th grade (ref.)	1.47	(1.22,1.77)	1.38	(1.16,1.65)	1.32	(0.82,2.13)	0.97	(0.61,1.54)	0.88	(0.53,1.46)
10 to 12th vs. Above 12th grade (ref.)	1.21	(1.01,1.44)	1.04	(0.89,1.23)	1.39	(0.90,2.15)	0.91	(0.58,1.44)	0.8	(0.48,1.33)
Household (Wealth Index)										
Poorest vs. Richest (ref.)	1.93	(1.57,2.37)	1.74	(1.45,2.08)	1.32	(0.94,1.86)	1.4	(1.06,1.83)	1.78	(1.33,2.37)
Poor vs. Richest (ref.)	1.94	(1.60,2.36)	1.61	(1.35,1.91)	1.15	(0.82,1.61)	1.18	(0.90,1.54)	1.66	(1.26,2.20)
Middle vs. Richest (ref.)	1.66	(1.37,2.01)	1.49	(1.26,1.76)	1.08	(0.79,1.50)	1.37	(1.06,1.77)	1.62	(1.24,2.12)
Rich vs. Richest (ref.)	1.56	(1.28,1.89)	1.28	(1.08,1.51)	1.11	(0.83,1.48)	1.21	(0.95,1.54)	1.32	(1.02,1.72)
Caste										
SC vs. Other (ref.)	1.24	(1.08,1.43)	1.13	(1.00,1.27)	1.13	(0.92,1.38)	1.17	(1.00,1.38)	1.2	(1.03,1.40)
ST vs. Other (ref.)	1.1	(0.94,1.29)	0.91	(0.79,1.04)	0.79	(0.61,1.03)	0.99	(0.80,1.22)	0.87	(0.71,1.05)
OBC vs. Other (ref.)	1.1	(0.97,1.25)	1.09	(0.98,1.22)	1	(0.83,1.20)	1.14	(0.99,1.31)		
Don't know vs. Other (ref.)	1.22	(0.82,1.82)	0.85	(0.58,1.23)	0.45	(0.09,2.38)				
No Caste vs. Other (ref.)	0.8	(0.62,1.03)	0.8	(0.63,1.01)	0.94	(0.62,1.41)	0.85	(0.48,1.51)		
Place of residence										
Rural vs. Urban (ref.)	1.11	(0.98,1.26)	1.2	(1.08,1.34)	1.19	(0.99,1.41)	1.13	(0.95,1.34)	1.07	(0.89,1.28)
Antenatal care visits, No.										
1-3 visits vs. None (ref.)	0.85	(0.71,1.01)	0.85	(0.76,0.95)	0.86	(0.67,1.11)	0.57	(0.47,0.69)	0.78	(0.67,0.92)
4 or more visits vs. None (ref.)	0.59	(0.49,0.71)	0.61	(0.54,0.69)	0.72	(0.53,0.96)	0.47	(0.36,0.62)	0.67	(0.55,0.82)
Don't know/missing vs. None (ref.)	1.8	(1.51,2.13)	2.05	(1.84,2.28)	2.2	(1.75,2.76)	0.42	(0.10,1.81)	0.08	(0.05,0.12)
Place of delivery										
Private facilities vs. Respondents' or other home (ref.)	1.58	(1.31,1.90)	1.33	(1.15,1.55)	1.71	(1.26,2.33)	1.72	(1.12,2.65)	1.26	(0.89,1.78)
Public facilities vs. Respondents' or other home (ref.)	1.25	(1.06,1.48)	1.06	(0.93,1.20)	1.24	(0.92,1.68)	1.68	(1.10,2.57)	1.37	(1.02,1.85)
Don't know/missing vs. Respondents' or other home (ref.)	2.07	(1.13,3.80)	1.79	(1.04,3.10)	4.92	(2.19,11.04)	4.45	(1.68,11.79)	3.54	(1.72,7.32)
Assistance during delivery										

Unskilled provider vs. Skilled provider (ref.)	1.19	(1.02,1.39)	1.07	(0.94,1.22)	1.09	(0.83,1.44)	1.13	(0.77,1.64)	1.08	(0.82,1.41)
Don't know/missing vs. Skilled provider (ref.)	2.88	(1.10,7.57)	6.01	(2.90,12.43)	1.36	(0.46,4.03)	0.1	(0.02,0.47)	1.01	(0.44,2.30)
Birth Size										
Average vs. Large (ref.)	0.85	(0.76,0.94)	0.78	(0.71,0.85)	0.9	(0.75,1.08)	0.77	(0.60,0.98)	0.65	(0.53,0.79)
Small vs. Large (ref.)	1.75	(1.54,1.99)	1.63	(1.46,1.83)	1.39	(1.14,1.70)	1.45	(1.13,1.87)	1.82	(1.49,2.22)
Don't know/missing vs. Large (ref.)	6.65	(5.54,7.99)	3.84	(3.27,4.51)	3.47	(2.41,5.01)	6.24	(1.55,25.14)	4.15	(2.80,6.15)

OR: Odds Ratio; CI: Confidence Interval

Table S19: Adjusted Logistic regression: Odds ratios and 95% confidence intervals for the association between covariates, maternal and newborn care, and late neonatal mortality (1993-2021), adjusted for twin birth.

	2019-21		2015-16		2005-06		1998-99		1992-93	
	OR	95 % CI	OR	95 % CI	OR	95 % CI	OR	95 % CI	OR	95 % CI
Child sex										
Boys vs. Girls (ref.)	1.17	(0.98,1.40)	1.35	(1.16,1.58)	0.78	(0.61,1.00)	1.2	(0.99,1.45)	1.09	(0.93,1.29)
Birth order										
2 to 3 vs. First (ref.)	1.03	(0.80,1.33)	1.09	(0.87,1.38)	0.88	(0.62,1.25)	0.87	(0.67,1.12)	0.73	(0.58,0.93)
4 or more vs. First (ref.)	1.49	(1.05,2.12)	1.32	(0.98,1.77)	1.13	(0.70,1.80)	1.23	(0.87,1.74)	0.7	(0.51,0.97)
Maternal age at birth										
less than 20 vs. 30 or more (ref.)	1.28	(0.89,1.83)	1.03	(0.75,1.41)	0.95	(0.58,1.58)	1.96	(1.34,2.88)	1.04	(0.73,1.49)
20 to 24 vs. 30 or more (ref.)	1.06	(0.80,1.39)	0.78	(0.60,1.02)	0.97	(0.63,1.48)	1.2	(0.86,1.68)	1	(0.74,1.34)
25 to 29 vs. 30 or more (ref.)	0.83	(0.65,1.07)	0.87	(0.67,1.12)	0.69	(0.46,1.03)	0.84	(0.61,1.15)	0.77	(0.59,1.02)
Maternal education										
No schooling vs. Above 12th grade (ref.)	1.39	(0.84,2.29)	0.99	(0.63,1.55)	2.77	(0.70,11.02)	1.7	(0.60,4.79)	2.18	(0.85,5.56)
1 to 5th vs. Above 12th grade (ref.)	1.58	(0.93,2.68)	1.13	(0.70,1.82)	3.05	(0.75,12.38)	1.51	(0.53,4.29)	1.42	(0.54,3.71)
6 to 8th vs. Above 12th grade (ref.)	1.16	(0.72,1.89)	1.24	(0.80,1.92)	2.83	(0.71,11.35)	1.06	(0.36,3.13)	1.62	(0.61,4.27)
10 to 12th vs. Above 12th grade (ref.)	1.05	(0.67,1.63)	1.02	(0.67,1.54)	2.33	(0.61,8.92)	1.07	(0.37,3.09)	2.01	(0.79,5.14)
Household (Wealth Index)										
Poorest vs. Richest (ref.)	1.26	(0.79,2.01)	1.4	(0.93,2.09)	1.91	(0.97,3.78)	1.93	(1.19,3.11)	1.57	(0.99,2.49)
Poor vs. Richest (ref.)	1.39	(0.88,2.19)	1.24	(0.84,1.82)	2.01	(1.03,3.93)	1.64	(1.02,2.64)	1.81	(1.15,2.83)
Middle vs. Richest (ref.)	1.2	(0.75,1.93)	1.15	(0.78,1.71)	1.91	(1.01,3.63)	1.81	(1.14,2.89)	1.64	(1.06,2.54)
Rich vs. Richest (ref.)	1.09	(0.70,1.71)	0.98	(0.65,1.47)	1.33	(0.70,2.53)	1.05	(0.65,1.69)	1.23	(0.80,1.89)
Caste										
SC vs. Other (ref.)	0.99	(0.74,1.33)	1.08	(0.83,1.42)	1.14	(0.77,1.68)	1.2	(0.92,1.57)	0.89	(0.70,1.13)
ST vs. Other (ref.)	1.01	(0.73,1.39)	0.84	(0.62,1.12)	1.12	(0.72,1.74)	1.44	(1.06,1.95)	1.05	(0.81,1.37)
OBC vs. Other (ref.)	0.85	(0.66,1.11)	0.99	(0.78,1.27)	0.93	(0.66,1.32)	1.11	(0.86,1.42)		
Don't know vs. Other (ref.)	1.82	(0.40,8.40)	0.73	(0.29,1.85)	0.94	(0.10,8.42)				
No Caste vs. Other (ref.)	0.53	(0.30,0.94)	0.43	(0.26,0.71)	0.97	(0.53,1.79)	1.81	(0.94,3.51)		
Place of residence										
Rural vs. Urban (ref.)	1.11	(0.84,1.46)	1.03	(0.82,1.30)	1.13	(0.80,1.59)	0.95	(0.71,1.26)	1.12	(0.86,1.47)
Antenatal care visits, No.										
1-3 visits vs. None (ref.)	0.81	(0.53,1.24)	0.86	(0.65,1.12)	0.94	(0.61,1.44)	0.77	(0.57,1.03)	0.67	(0.54,0.85)
4 or more visits vs. None (ref.)	0.75	(0.49,1.14)	0.63	(0.46,0.85)	0.65	(0.38,1.11)	0.48	(0.30,0.77)	0.51	(0.37,0.71)
Don't know/missing vs. None (ref.)	2.07	(1.37,3.13)	2.41	(1.87,3.10)	2.01	(1.37,2.94)	3.58	(1.15,11.12)	0.08	(0.04,0.13)
Place of delivery										
Private facilities vs. Respondents’ or other home (ref.)	1.16	(0.76,1.76)	0.77	(0.54,1.09)	1.53	(0.83,2.84)	1.09	(0.53,2.26)	0.73	(0.40,1.35)
Public facilities vs. Respondents’ or other home (ref.)	1.18	(0.81,1.73)	0.8	(0.60,1.08)	1.69	(0.94,3.06)	1.74	(0.90,3.35)	0.94	(0.60,1.50)
Don't know/missing vs. Respondents’ or other home (ref.)	1.43	(0.36,5.63)	0.14	(0.02,0.84)	8.03	(2.54,25.37)	3.35	(0.55,20.58)	5.24	(2.58,10.62)
Assistance during delivery										
Unskilled provider vs. Skilled provider (ref.)	1.33	(0.91,1.96)	1.01	(0.76,1.36)	1.61	(0.95,2.72)	1.17	(0.67,2.04)	1.25	(0.85,1.83)
Don't know/missing vs. Skilled provider (ref.)	5.85	(0.92,37.37)	59	(7.99,435.86)	4.47	(0.75,26.68)	0.08	(0.01,0.83)	2.31	(0.91,5.85)
Birth Size										

Average vs. Large (ref.)	0.8	(0.64,1.01)	0.92	(0.74,1.15)	0.79	(0.56,1.11)	1.12	(0.72,1.76)	0.84	(0.61,1.15)
Small vs. Large (ref.)	2.03	(1.55,2.67)	2.48	(1.88,3.28)	1.74	(1.22,2.48)	1.9	(1.19,3.03)	1.98	(1.43,2.74)
Don't know/missing vs. Large (ref.)	2.17	(1.24,3.78)	1.99	(1.29,3.07)	0.84	(0.32,2.20)	1.67	(0.12,23.09)	1.32	(0.71,2.46)

OR: Odds Ratio; CI: Confidence Interval

Table S20: Adjusted Logistic regression: Odds ratios and 95% confidence intervals for the association between covariates, maternal and newborn care, and post neonatal mortality (1993-2021), adjusted for twin birth.

	2019-21		2015-16		2005-06		1998-99		1992-93	
	OR	95 % CI	OR	95 % CI	OR	95 % CI	OR	95 % CI	OR	95 % CI
Child sex										
Boys vs. Girls (ref.)	1.19	(1.06,1.34)	0.98	(0.89,1.09)	0.78	(0.65,0.94)	0.89	(0.78,1.02)	0.93	(0.83,1.06)
Birth order										
2 to 3 vs. First (ref.)	1.33	(1.14,1.55)	1.15	(0.99,1.34)	0.98	(0.77,1.26)	0.91	(0.76,1.10)	0.92	(0.77,1.11)
4 or more vs. First (ref.)	1.89	(1.52,2.36)	1.69	(1.38,2.07)	1.15	(0.83,1.61)	1.18	(0.92,1.50)	1.18	(0.93,1.48)
Maternal age at birth										
less than 20 vs. 30 or more (ref.)	1.48	(1.13,1.93)	1.2	(0.95,1.51)	1.42	(0.99,2.04)	1.62	(1.23,2.12)	1.23	(0.95,1.59)
20 to 24 vs. 30 or more (ref.)	1.21	(1.00,1.48)	0.86	(0.73,1.02)	0.99	(0.73,1.34)	1.2	(0.95,1.50)	1.1	(0.90,1.35)
25 to 29 vs. 30 or more (ref.)	1.1	(0.93,1.31)	0.89	(0.77,1.03)	1.05	(0.79,1.40)	1.09	(0.88,1.35)	0.78	(0.64,0.96)
Maternal education										
No schooling vs. Above 12th grade (ref.)	1.48	(1.11,1.97)	1.58	(1.14,2.20)	3.03	(1.32,6.98)	3.52	(1.53,8.12)	4.52	(1.74,11.74)
1 to 5th vs. Above 12th grade (ref.)	1.49	(1.09,2.04)	1.5	(1.08,2.09)	2.3	(1.00,5.32)	3.29	(1.43,7.58)	3.88	(1.49,10.13)
6 to 8th vs. Above 12th grade (ref.)	1.23	(0.92,1.65)	1.32	(0.95,1.84)	1.99	(0.87,4.58)	2.24	(0.96,5.22)	3.59	(1.36,9.46)
10 to 12th vs. Above 12th grade (ref.)	1.08	(0.82,1.42)	1.31	(0.97,1.78)	1.53	(0.69,3.40)	1.61	(0.69,3.75)	3.02	(1.15,7.92)
Household (Wealth Index)										
Poorest vs. Richest (ref.)	1.65	(1.25,2.18)	1.48	(1.13,1.93)	1.24	(0.74,2.06)	1.72	(1.22,2.43)	1.65	(1.22,2.24)
Poor vs. Richest (ref.)	1.54	(1.18,2.03)	1.49	(1.15,1.94)	1.6	(0.98,2.59)	1.44	(1.03,2.01)	1.25	(0.92,1.70)
Middle vs. Richest (ref.)	1.61	(1.22,2.13)	1.5	(1.15,1.96)	1.31	(0.82,2.10)	1.35	(0.97,1.87)	1.21	(0.90,1.62)
Rich vs. Richest (ref.)	1.53	(1.13,2.06)	1.21	(0.94,1.56)	1.08	(0.68,1.74)	1.02	(0.73,1.41)	1.13	(0.85,1.49)
Caste										
SC vs. Other (ref.)	1.11	(0.90,1.36)	1.05	(0.89,1.24)	0.96	(0.73,1.26)	1.07	(0.89,1.30)	1.13	(0.96,1.33)
ST vs. Other (ref.)	1.09	(0.88,1.35)	1	(0.83,1.21)	0.96	(0.70,1.32)	1.2	(0.97,1.49)	0.86	(0.70,1.05)
OBC vs. Other (ref.)	0.99	(0.82,1.20)	1.08	(0.93,1.25)	0.92	(0.72,1.18)	1.09	(0.92,1.29)		
Don't know vs. Other (ref.)	1.53	(0.61,3.82)	0.88	(0.51,1.51)	1.98	(0.81,4.82)				
No Caste vs. Other (ref.)	0.93	(0.62,1.40)	1.06	(0.74,1.52)	0.58	(0.32,1.06)	2.03	(1.30,3.18)		
Place of residence										
Rural vs. Urban (ref.)	0.91	(0.77,1.07)	1.01	(0.86,1.19)	0.98	(0.77,1.26)	1	(0.82,1.23)	0.9	(0.75,1.09)
Antenatal care visits, No.										
1-3 visits vs. None (ref.)	0.85	(0.64,1.11)	0.87	(0.73,1.03)	1.03	(0.74,1.44)	0.62	(0.50,0.78)	0.6	(0.51,0.71)
4 or more visits vs. None (ref.)	0.68	(0.51,0.90)	0.68	(0.56,0.83)	1.24	(0.83,1.86)	0.6	(0.43,0.84)	0.44	(0.35,0.56)
Don't know/missing vs. None (ref.)	2.24	(1.71,2.92)	1.98	(1.69,2.32)	2.79	(2.06,3.79)	1.2	(0.41,3.53)	0.05	(0.03,0.08)
Place of delivery										
Private facilities vs. Respondents' or other home (ref.)	1.19	(0.91,1.55)	0.9	(0.72,1.12)	0.88	(0.59,1.31)	1.15	(0.73,1.82)	0.9	(0.59,1.39)
Public facilities vs. Respondents' or other home (ref.)	1.05	(0.84,1.30)	1.05	(0.87,1.27)	0.54	(0.37,0.80)	1.35	(0.89,2.05)	1.07	(0.77,1.50)
Don't know/missing vs. Respondents' or other home (ref.)	3.28	(1.58,6.84)	1.91	(1.07,3.40)	1.13	(0.58,2.20)	0.44	(0.12,1.56)	4.94	(2.94,8.29)
Assistance during delivery										
Unskilled provider vs. Skilled provider (ref.)	1.08	(0.87,1.33)	1.09	(0.90,1.31)	1.03	(0.75,1.42)	1.16	(0.82,1.64)	1.11	(0.85,1.46)
Don't know/missing vs. Skilled provider (ref.)	2.97	(0.80,11.07)	36.97	(13.80,99.02)	13.75	(5.91,31.97)	0.33	(0.04,2.83)	2.72	(1.49,4.98)
Birth Size										

Average vs. Large (ref.)	1.21	(1.04,1.42)	0.9	(0.79,1.04)	1.07	(0.84,1.37)	0.95	(0.70,1.30)	1.04	(0.82,1.31)
Small vs. Large (ref.)	2.02	(1.64,2.49)	1.61	(1.36,1.91)	1.54	(1.18,2.02)	1.3	(0.94,1.80)	2.02	(1.58,2.59)
Don't know/missing vs. Large (ref.)	3.19	(2.25,4.51)	2.08	(1.52,2.83)	0.62	(0.30,1.27)	7.88	(1.84,33.66)	1.98	(1.19,3.29)

OR: Odds Ratio; CI: Confidence Interval

Table S21: Adjusted Logistic regression: Odds ratios and 95% confidence intervals for the association between covariates, maternal and newborn care, and child mortality (1993-2021), adjusted for twin birth.

	2019-21		2015-16		2005-06		1998-99		1992-93	
	OR	95 % CI	OR	95 % CI	OR	95 % CI	OR	95 % CI	OR	95 % CI
Child sex										
Boys vs. Girls (ref.)	1.08	(0.89,1.32)	0.97	(0.81,1.15)	0.65	(0.50,0.84)	0.55	(0.46,0.66)	0.6	(0.51,0.71)
Birth order										
2 to 3 vs. First (ref.)	1.56	(1.20,2.03)	1.21	(0.96,1.51)	1.44	(0.99,2.11)	1.37	(1.05,1.79)	1.16	(0.91,1.49)
4 or more vs. First (ref.)	2.12	(1.49,3.01)	1.78	(1.36,2.32)	1.76	(1.11,2.79)	2.15	(1.55,2.98)	1.53	(1.12,2.09)
Maternal age at birth										
less than 20 vs. 30 or more (ref.)	0.95	(0.64,1.41)	0.71	(0.50,1.02)	1.09	(0.67,1.76)	1.47	(1.04,2.06)	1.24	(0.87,1.77)
20 to 24 vs. 30 or more (ref.)	0.83	(0.63,1.11)	0.78	(0.61,1.00)	0.89	(0.61,1.31)	1.14	(0.87,1.51)	1.28	(0.97,1.69)
25 to 29 vs. 30 or more (ref.)	0.73	(0.56,0.96)	0.86	(0.70,1.07)	0.88	(0.60,1.30)	0.81	(0.62,1.05)	1.21	(0.93,1.57)
Maternal education										
No schooling vs. Above 12th grade (ref.)	2.11	(1.14,3.94)	1.95	(1.11,3.43)	3.03	(0.75,12.22)	1.96	(0.52,7.30)	3.96	(0.70,22.54)
1 to 5th vs. Above 12th grade (ref.)	1.52	(0.80,2.90)	2.04	(1.14,3.66)	2.06	(0.50,8.46)	1.43	(0.37,5.48)	2.29	(0.39,13.30)
6 to 8th vs. Above 12th grade (ref.)	1.69	(0.87,3.27)	1.46	(0.83,2.58)	1.05	(0.23,4.72)	1.36	(0.36,5.16)	2.33	(0.40,13.64)
10 to 12th vs. Above 12th grade (ref.)	1.24	(0.67,2.31)	1.27	(0.68,2.38)	1.05	(0.25,4.40)	1.18	(0.31,4.48)	1.34	(0.22,8.21)
Household (Wealth Index)										
Poorest vs. Richest (ref.)	1.81	(1.04,3.13)	2.29	(1.19,4.40)	1.6	(0.75,3.42)	4.77	(2.65,8.59)	2.86	(1.71,4.76)
Poor vs. Richest (ref.)	1.55	(0.90,2.65)	1.93	(1.05,3.57)	1.7	(0.81,3.58)	3.4	(1.90,6.08)	2.74	(1.65,4.53)
Middle vs. Richest (ref.)	1.34	(0.77,2.34)	1.76	(0.88,3.51)	1.36	(0.65,2.85)	2.69	(1.49,4.87)	2.26	(1.37,3.72)
Rich vs. Richest (ref.)	1.11	(0.62,2.00)	1.59	(0.95,2.69)	0.91	(0.43,1.95)	1.58	(0.90,2.80)	1.89	(1.15,3.11)
Caste										
SC vs. Other (ref.)	1.29	(0.89,1.88)	1.27	(0.85,1.89)	1.63	(1.08,2.48)	1.02	(0.80,1.29)	1.11	(0.88,1.39)
ST vs. Other (ref.)	1.03	(0.70,1.51)	1.36	(0.93,1.97)	2.24	(1.45,3.45)	1.4	(1.09,1.81)	1.2	(0.94,1.52)
OBC vs. Other (ref.)	1.11	(0.79,1.57)	1.16	(0.79,1.69)	1.06	(0.71,1.59)	0.93	(0.74,1.16)		
Don't know vs. Other (ref.)	0.87	(0.15,5.02)	1.37	(0.59,3.17)	1	(1.00,1.00)				
No Caste vs. Other (ref.)	0.54	(0.25,1.15)	0.81	(0.45,1.46)	1.12	(0.48,2.65)	1.75	(1.01,3.05)		
Place of residence										
Rural vs. Urban (ref.)	0.79	(0.57,1.09)	0.85	(0.61,1.18)	0.89	(0.62,1.28)	0.77	(0.58,1.03)	0.93	(0.71,1.21)
Antenatal care visits, No.										
1-3 visits vs. None (ref.)	0.92	(0.58,1.46)	0.77	(0.59,1.00)	0.67	(0.41,1.07)	0.51	(0.33,0.76)	0.53	(0.42,0.69)
4 or more visits vs. None (ref.)	0.77	(0.48,1.24)	0.54	(0.39,0.75)	0.83	(0.45,1.53)	0.57	(0.30,1.07)	0.51	(0.35,0.74)
Don't know/missing vs. None (ref.)	2.68	(1.73,4.16)	3.06	(2.44,3.83)	3.09	(2.11,4.51)	0.7	(0.34,1.44)	0.08	(0.04,0.16)
Place of delivery										
Private facilities vs. Respondents’ or other home (ref.)	0.6	(0.39,0.93)	1.03	(0.72,1.49)	0.98	(0.48,1.98)	0.49	(0.16,1.52)	0.61	(0.26,1.46)
Public facilities vs. Respondents’ or other home (ref.)	0.7	(0.50,0.99)	0.85	(0.67,1.08)	0.55	(0.28,1.08)	1.31	(0.57,3.04)	1.35	(0.80,2.26)
Don't know/missing vs. Respondents’ or other home (ref.)	0.95	(0.23,4.00)	1.32	(0.50,3.48)	0.82	(0.11,5.94)	4.49	(0.93,21.68)	4.13	(1.46,11.64)
Assistance during delivery										
Unskilled provider vs. Skilled provider (ref.)	1.04	(0.74,1.47)	1.24	(0.97,1.57)	1.01	(0.61,1.69)	1.33	(0.70,2.51)	1.07	(0.71,1.60)
Don't know/missing vs. Skilled provider (ref.)	20.25	(2.83,144.82)	1	(1.00,1.00)	38.79	(4.27,352.64)	0.15	(0.02,1.52)	2.62	(0.74,9.19)
Birth Size										

Average vs. Large (ref.)	1.1	(0.82,1.47)	1	(0.79,1.27)	0.95	(0.68,1.32)	1.27	(0.71,2.28)	0.86	(0.62,1.18)
Small vs. Large (ref.)	1.02	(0.70,1.49)	1.3	(0.98,1.74)	1.09	(0.73,1.60)	0.91	(0.48,1.73)	0.85	(0.58,1.23)
Don't know/missing vs. Large (ref.)	1.8	(1.02,3.16)	1.1	(0.69,1.75)	0.58	(0.23,1.50)	8.92	(1.67,47.62)	2.58	(1.37,4.87)

OR: Odds Ratio; CI: Confidence Interval

Table S22: Adjusted Logistic regression: Odds ratios and 95% confidence intervals for the association between maternal and newborn care and early neonatal mortality (1993-2021), adjusted for twin birth.

	2019-21		2015-16		2005-06		1998-99		1992-93	
	OR	95 % CI	OR	95 % CI	OR	95 % CI	OR	95 % CI	OR	95 % CI
Antenatal care visits, No.										
1-3 visits vs. None (ref.)	0.81	(0.68,0.97)	0.8	(0.72,0.90)	0.81	(0.63,1.04)	0.55	(0.45,0.66)	0.76	(0.65,0.89)
4 or more visits vs. None (ref.)	0.51	(0.43,0.62)	0.51	(0.44,0.57)	0.56	(0.42,0.74)	0.41	(0.31,0.54)	0.61	(0.49,0.75)
Don't know/missing vs. None (ref.)	1.74	(1.47,2.06)	1.98	(1.78,2.19)	2.16	(1.73,2.70)	0.39	(0.09,1.73)	0.08	(0.05,0.12)
Place of delivery										
Private facilities vs. Respondents' or other home (ref.)	1.2	(1.00,1.43)	1.09	(0.95,1.26)	1.45	(1.08,1.96)	1.57	(1.02,2.43)	1.01	(0.72,1.43)
Public facilities vs. Respondents' or other home (ref.)	1.2	(1.02,1.41)	1.04	(0.91,1.19)	1.18	(0.88,1.59)	1.66	(1.08,2.53)	1.28	(0.95,1.73)
Don't know/missing vs. Respondents' or other home (ref.)	1.83	(1.00,3.35)	1.78	(1.04,3.05)	4.77	(2.12,10.74)	4.47	(1.73,11.57)	3.36	(1.66,6.81)
Assistance during delivery										
Unskilled provider vs. Skilled provider (ref.)	1.24	(1.06,1.45)	1.12	(0.98,1.27)	1.15	(0.87,1.50)	1.17	(0.80,1.70)	1.11	(0.85,1.46)
Don't know/missing vs. Skilled provider (ref.)	3.09	(1.23,7.78)	5.73	(2.84,11.59)	1.36	(0.46,3.96)	0.09	(0.02,0.43)	1.07	(0.48,2.37)
Birth Size										
Average vs. Large (ref.)	0.84	(0.76,0.93)	0.78	(0.71,0.86)	0.89	(0.74,1.06)	0.77	(0.60,0.98)	0.64	(0.53,0.78)
Small vs. Large (ref.)	1.78	(1.57,2.02)	1.68	(1.50,1.88)	1.41	(1.16,1.72)	1.48	(1.15,1.91)	1.86	(1.52,2.27)
Don't know/missing vs. Large (ref.)	6.99	(5.82,8.39)	3.93	(3.34,4.61)	3.42	(2.39,4.91)	6.61	(1.78,24.59)	4.2	(2.85,6.20)

OR: Odds Ratio; CI: Confidence Interval

Table S23: Adjusted Logistic regression: Odds ratios and 95% confidence intervals for the association between maternal and newborn care and late neonatal mortality (1993-2021), adjusted for twin birth.

	2019-21		2015-16		2005-06		1998-99		1992-93	
	OR	95 % CI	OR	95 % CI	OR	95 % CI	OR	95 % CI	OR	95 % CI
Antenatal care visits, No.										
1-3 visits vs. None (ref.)	0.77	(0.50,1.17)	0.81	(0.62,1.06)	0.89	(0.58,1.36)	0.71	(0.53,0.96)	0.64	(0.51,0.80)
4 or more visits vs. None (ref.)	0.64	(0.42,0.97)	0.54	(0.40,0.74)	0.5	(0.29,0.85)	0.34	(0.21,0.55)	0.45	(0.32,0.62)
Don't know/missing vs. None (ref.)	1.92	(1.28,2.87)	2.11	(1.66,2.68)	1.92	(1.31,2.80)	3.28	(1.06,10.11)	0.07	(0.04,0.12)
Place of delivery										
Private facilities vs. Respondents’ or other home (ref.)	0.84	(0.56,1.24)	0.66	(0.47,0.92)	1.13	(0.62,2.06)	0.87	(0.42,1.83)	0.58	(0.32,1.05)
Public facilities vs. Respondents’ or other home (ref.)	1.06	(0.73,1.53)	0.77	(0.57,1.03)	1.54	(0.87,2.75)	1.68	(0.87,3.22)	0.86	(0.54,1.36)
Don't know/missing vs. Respondents’ or other home (ref.)	1.24	(0.32,4.90)	0.14	(0.02,0.84)	7.76	(2.67,22.53)	3.47	(0.57,21.05)	4.71	(2.40,9.27)
Assistance during delivery										
Unskilled provider vs. Skilled provider (ref.)	1.4	(0.96,2.05)	1.06	(0.79,1.41)	1.75	(1.04,2.93)	1.35	(0.78,2.34)	1.32	(0.89,1.95)
Don't know/missing vs. Skilled provider (ref.)	7.62	(1.21,48.08)	63.59	(8.65,467.19)	4.31	(0.89,20.85)	0.09	(0.01,0.87)	2.58	(1.05,6.32)
Birth Size										
Average vs. Large (ref.)	0.81	(0.64,1.02)	0.93	(0.75,1.17)	0.78	(0.55,1.10)	1.13	(0.72,1.77)	0.84	(0.61,1.15)
Small vs. Large (ref.)	2.07	(1.58,2.73)	2.51	(1.91,3.30)	1.79	(1.26,2.55)	2	(1.26,3.17)	2.01	(1.45,2.78)
Don't know/missing vs. Large (ref.)	2.31	(1.32,4.05)	2	(1.29,3.09)	0.89	(0.34,2.30)	1.59	(0.12,21.37)	1.37	(0.73,2.54)

OR: Odds Ratio; CI: Confidence Interval

Table S24: Adjusted Logistic regression: Odds ratios and 95% confidence intervals for the association between maternal and newborn care and post neonatal mortality (1993-2021), adjusted for twin birth.

	2019-21		2015-16		2005-06		1998-99		1992-93	
	OR	95 % CI	OR	95 % CI	OR	95 % CI	OR	95 % CI	OR	95 % CI
Antenatal care visits, No.										
1-3 visits vs. None (ref.)	0.8	(0.60,1.05)	0.8	(0.67,0.94)	0.95	(0.68,1.32)	0.57	(0.46,0.70)	0.56	(0.48,0.66)
4 or more visits vs. None (ref.)	0.58	(0.43,0.76)	0.54	(0.45,0.66)	0.93	(0.62,1.38)	0.41	(0.29,0.58)	0.37	(0.29,0.47)
Don't know/missing vs. None (ref.)	1.96	(1.51,2.56)	1.71	(1.47,1.98)	2.67	(1.98,3.60)	1.09	(0.37,3.19)	0.05	(0.03,0.08)
Place of delivery										
Private facilities vs. Respondents' or other home (ref.)	0.81	(0.63,1.04)	0.67	(0.54,0.83)	0.65	(0.45,0.95)	0.89	(0.56,1.40)	0.69	(0.46,1.05)
Public facilities vs. Respondents' or other home (ref.)	0.9	(0.73,1.11)	0.96	(0.80,1.16)	0.47	(0.32,0.70)	1.26	(0.83,1.92)	0.99	(0.71,1.38)
Don't know/missing vs. Respondents' or other home (ref.)	2.79	(1.35,5.78)	1.81	(1.02,3.22)	1.31	(0.70,2.46)	0.43	(0.12,1.48)	4.82	(2.93,7.93)
Assistance during delivery										
Unskilled provider vs. Skilled provider (ref.)	1.14	(0.92,1.41)	1.17	(0.98,1.41)	1.15	(0.84,1.58)	1.36	(0.96,1.92)	1.21	(0.92,1.59)
Don't know/missing vs. Skilled provider (ref.)	3.23	(0.90,11.61)	43.62	(16.62,114.53	11.4	(5.18,25.11)	0.4	(0.05,3.12)	2.87	(1.59,5.17)
Birth Size										
Average vs. Large (ref.)	1.22	(1.04,1.42)	0.91	(0.79,1.05)	1.06	(0.83,1.36)	0.98	(0.72,1.33)	1.03	(0.82,1.31)
Small vs. Large (ref.)	2.05	(1.66,2.53)	1.66	(1.40,1.97)	1.59	(1.22,2.08)	1.39	(1.00,1.93)	2.07	(1.61,2.65)
Don't know/missing vs. Large (ref.)	3.48	(2.46,4.92)	2.18	(1.61,2.96)	0.63	(0.31,1.27)	7.36	(1.82,29.72)	2.05	(1.24,3.38)

OR: Odds Ratio; CI: Confidence Interval

Table S25: Adjusted Logistic regression: Odds ratios and 95% confidence intervals for the association between maternal and newborn care, maternal nutrition, and child mortality (1993-2021), adjusted for twin birth.

CMR	2019-21		2015-16		2005-06		1998-99		1992-93	
	OR	95 % CI	OR	95 % CI	OR	95 % CI	OR	95 % CI	OR	95 % CI
Antenatal care visits, No.										
1-3 visits vs. None (ref.)	0.82	(0.52,1.31)	0.67	(0.51,0.87)	0.56	(0.35,0.90)	0.42	(0.28,0.64)	0.46	(0.36,0.59)
4 or more visits vs. None (ref.)	0.58	(0.36,0.93)	0.37	(0.27,0.51)	0.52	(0.28,0.94)	0.33	(0.17,0.65)	0.36	(0.24,0.53)
Don't know/missing vs. None (ref.)	2.1	(1.36,3.25)	2.38	(1.91,2.97)	2.59	(1.77,3.79)	0.57	(0.27,1.21)	0.06	(0.03,0.13)
Place of delivery										
Private facilities vs. Respondents' or other home (ref.)	0.33	(0.22,0.50)	0.64	(0.44,0.92)	0.57	(0.29,1.11)	0.33	(0.11,1.04)	0.32	(0.13,0.78)
Public facilities vs. Respondents' or other home (ref.)	0.54	(0.38,0.75)	0.73	(0.58,0.92)	0.42	(0.22,0.79)	1.19	(0.52,2.74)	1.07	(0.63,1.82)
Don't know/missing vs. Respondents' or other home (ref.)	0.68	(0.16,2.83)	1.23	(0.47,3.23)	0.71	(0.12,4.10)	4.66	(0.99,22.08)	3.86	(1.36,11.00)
Assistance during delivery										
Unskilled provider vs. Skilled provider (ref.)	1.12	(0.79,1.58)	1.42	(1.12,1.79)	1.41	(0.87,2.29)	1.84	(0.98,3.44)	1.3	(0.86,1.95)
Don't know/missing vs. Skilled provider (ref.)	30.14	(4.04,225.17)	1	(1.00,1.00)	32	(5.73,178.64)	0.27	(0.02,3.82)	2.83	(0.80,10.01)
Birth Size										
Average vs. Large (ref.)	1.13	(0.84,1.51)	1.03	(0.82,1.31)	0.97	(0.70,1.35)	1.34	(0.75,2.39)	0.86	(0.62,1.17)
Small vs. Large (ref.)	1.05	(0.72,1.53)	1.37	(1.03,1.83)	1.15	(0.78,1.70)	1.03	(0.54,1.95)	0.88	(0.61,1.28)
Don't know/missing vs. Large (ref.)	2.07	(1.17,3.64)	1.22	(0.76,1.94)	0.65	(0.25,1.68)	6.35	(0.98,40.98)	2.9	(1.54,5.47)

OR: Odds Ratio; CI: Confidence Interval

Table S26: Sensitivity analysis including higher birth order and missing maternal education.

**NFHS**		**Primary Analysis**	**Sensitivity (Including higher order and missing maternal education)**	
		**Twin**	**Twin**	**Higher**
2019-21	ENM	2.0305	2.0306	1.9594
	LNM	2.3043	2.3029	2.8057
	PNM	1.1460	1.1480	3.5004
	CM	0.2469	0.2469	-
2015-16	ENM	1.9389	1.9391	3.3032
	LNM	2.1512	2.1513	2.5614
	PNM	1.4476	1.4477	3.0175
	CM	0.0261	0.0261	-
2005-06	ENM	1.8897	1.8903	3.7159
	LNM	1.9471	1.9471	0.7154
	PNM	1.7361	1.7361	-
	CM	0.7278	0.7278	-
1998-99	ENM	2.0154	2.0154	-
	LNM	1.7630	1.7635	4.3945
	PNM	1.5686	1.5697	3.0443
	CM	-0.2807	-0.2807	-
1992-93	ENM	2.3728	2.3708	3.3556
	LNM	2.6270	2.6119	4.4574
	PNM	1.6629	1.6566	-
	CM	0.0068	0.0024	-

Table S27: Adjusted logistic regression: odds ratios and 95% confidence intervals for the association between covariates and early neonatal mortality (1993–2021), adjusted for twin birth, and the interaction between maternal education and wealth index.

	2019-21		2015-16		2005-06		1998-99		1992-93	
	OR	95 % CI	OR	95 % CI	OR	95 % CI	OR	95 % CI	OR	95 % CI
Sex of child										
Boy vs. Girls (ref.)	1.24	(1.15,1.34)	1.33	(1.25,1.43)	1.31	(1.14,1.49)	1.12	(1.00,1.25)	1.29	(1.15,1.44)
Birth order										
2 to 3 vs. First (ref.)	0.69	(0.63,0.75)	0.64	(0.59,0.69)	0.57	(0.48,0.67)	0.79	(0.68,0.92)	0.61	(0.52,0.71)
4 or more vs. First (ref.)	0.87	(0.75,1.01)	0.79	(0.70,0.89)	0.79	(0.62,1.02)	0.9	(0.72,1.11)	0.62	(0.50,0.77)
Maternal age at birth										
less than 20 vs. 30 or more (ref.)	1.27	(1.07,1.49)	1.17	(1.01,1.35)	1.57	(1.16,2.12)	1.4	(1.10,1.79)	1.26	(0.99,1.60)
20 to 24 vs. 30 or more (ref.)	0.98	(0.86,1.11)	0.97	(0.87,1.09)	1.3	(1.00,1.68)	1	(0.81,1.23)	0.92	(0.75,1.14)
25 to 29 vs. 30 or more (ref.)	0.89	(0.79,1.00)	0.85	(0.76,0.94)	0.93	(0.73,1.20)	0.84	(0.69,1.02)	0.9	(0.74,1.08)
Maternal education vs, Wealth										
Higher x richest (ref.)										
No Schooling x poorest	5.04	(3.85,6.61)	3.55	(2.79,4.52)	2.62	(1.61,4.24)	1.58	(0.98,2.54)	2.84	(1.64,4.91)
No Schooling x poorer	4.76	(3.58,6.31)	3.04	(2.38,3.89)	2.07	(1.26,3.38)	1.29	(0.80,2.08)	2.68	(1.55,4.63)
No Schooling x middle	3.89	(2.79,5.42)	3.22	(2.47,4.18)	2.01	(1.22,3.31)	1.64	(1.01,2.64)	2.64	(1.53,4.57)
No Schooling x richer	3.48	(2.36,5.12)	3.39	(2.49,4.61)	2.26	(1.33,3.83)	1.3	(0.80,2.11)	1.95	(1.12,3.38)
No Schooling x richest	3.81	(2.29,6.33)	2.52	(1.60,3.99)	1.98	(0.89,4.38)	0.77	(0.39,1.53)	1.08	(0.56,2.09)
Primary x poorest	4.43	(3.28,5.98)	3.82	(2.93,4.98)	1.86	(1.00,3.47)	1.58	(0.88,2.84)	2.28	(1.10,4.75)
Primary x poorer	4.49	(3.31,6.08)	3.37	(2.60,4.38)	2.53	(1.47,4.35)	0.83	(0.46,1.50)	2.36	(1.25,4.44)
Primary x middle	3.86	(2.72,5.49)	2.95	(2.21,3.93)	2.21	(1.29,3.76)	1.3	(0.77,2.19)	2.1	(1.13,3.88)
Primary x richer	3.07	(2.02,4.67)	3.06	(2.22,4.22)	1.82	(0.99,3.34)	1.22	(0.72,2.07)	1.88	(1.03,3.42)
Primary x richest	2.23	(1.17,4.23)	1.84	(1.11,3.06)	3.14	(1.32,7.49)	0.8	(0.41,1.56)	1.39	(0.70,2.72)
Middle x poorest	4.44	(3.32,5.95)	3.19	(2.42,4.21)	1.44	(0.66,3.14)	0.94	(0.38,2.34)	2.94	(1.22,7.08)
Middle x poorer	4.41	(3.31,5.88)	3.17	(2.38,4.21)	1.38	(0.74,2.57)	1.35	(0.71,2.58)	1.5	(0.67,3.36)
Middle x middle	3.59	(2.62,4.93)	2.68	(2.04,3.52)	1.05	(0.57,1.95)	0.82	(0.44,1.52)	1.92	(0.99,3.74)
Middle x richer	3.52	(2.52,4.91)	2.25	(1.66,3.06)	1.62	(0.93,2.83)	1.32	(0.79,2.22)	1.7	(0.92,3.13)
Middle x richest	2.11	(1.36,3.26)	2.02	(1.34,3.03)	1.86	(1.01,3.42)	0.87	(0.47,1.59)	1.27	(0.66,2.41)
Secondary x poorest	3.92	(2.90,5.30)	3.31	(2.44,4.47)	2.74	(1.20,6.22)	1	(1.00,1.00)	0.95	(0.15,6.00)
Secondary x poorer	3.21	(2.43,4.26)	2.26	(1.74,2.94)	1.69	(0.82,3.48)	1.89	(0.96,3.74)	2.01	(0.80,5.08)
Secondary x middle	2.67	(2.02,3.53)	1.76	(1.34,2.31)	1.72	(0.95,3.10)	1.1	(0.59,2.03)	1.3	(0.58,2.93)
Secondary x richer	2.56	(1.93,3.39)	1.49	(1.12,1.99)	1.42	(0.83,2.41)	0.78	(0.45,1.35)	1.28	(0.67,2.45)
Secondary x richest	1.98	(1.44,2.72)	1.27	(0.96,1.69)	0.94	(0.55,1.62)	0.88	(0.53,1.48)	1.21	(0.67,2.17)
Higher x poorest	3.99	(2.38,6.67)	4.13	(2.25,7.60)	1	(1.00,1.00)	1	(1.00,1.00)	1	(1.00,1.00)
Higher x poorer	3.28	(2.14,5.04)	2.71	(1.79,4.09)	4.72	(0.64,34.60)	1	(1.00,1.00)	1	(1.00,1.00)
Higher x middle	2.52	(1.77,3.59)	2.36	(1.61,3.45)	1.8	(0.37,8.73)	1	(1.00,1.00)	1	(1.00,1.00)
Higher x richer	2.32	(1.56,3.45)	1.16	(0.82,1.62)	0.31	(0.07,1.31)	0.46	(0.11,1.85)	4.35	(1.64,11.53)

Caste										
SC vs. Other (ref.)	1.21	(1.06,1.39)	1.1	(0.98,1.24)	1.09	(0.89,1.33)	1.17	(1.00,1.38)	1.24	(1.06,1.44)
ST vs. Other (ref.)	1.02	(0.87,1.19)	0.92	(0.80,1.05)	0.8	(0.61,1.03)	1	(0.81,1.24)	0.93	(0.77,1.13)
OBC vs. Other (ref.)	1.09	(0.96,1.24)	1.11	(1.00,1.24)	0.97	(0.81,1.17)	1.16	(1.01,1.33)		
Don't know vs. Other (ref.)	1.2	(0.81,1.77)	0.97	(0.67,1.40)	0.56	(0.13,2.44)				
No Caste vs. Other (ref.)	0.71	(0.55,0.92)	0.78	(0.62,0.98)	0.91	(0.61,1.38)	0.85	(0.48,1.52)		
Place of residence										
Rural vs. Urban (ref.)	1.09	(0.96,1.24)	1.22	(1.10,1.36)	1.15	(0.96,1.37)	1.13	(0.95,1.33)	1.02	(0.86,1.21)

OR: Odds Ratio; CI: Confidence Interval

Table S28: Adjusted Logistic Regression: Odds ratios and 95% confidence intervals for the association between covariates and late neonatal mortality (1993–2021), adjusted for twin birth, and the interaction between maternal education and wealth index.

	2019-21		2015-16		2005-06		1998-99		1992-93	
	OR	95 % CI	OR	95 % CI	OR	95 % CI	OR	95 % CI	OR	95 % CI
Sex of child										
Boy vs. Girls (ref.)	1.08	(0.91,1.29)	1.22	(1.05,1.42)	0.74	(0.58,0.95)	1.18	(0.98,1.43)	1.05	(0.89,1.25)
Birth order										
2 to 3 vs. First (ref.)	0.85	(0.67,1.08)	0.88	(0.71,1.08)	0.77	(0.55,1.08)	0.88	(0.68,1.13)	0.76	(0.60,0.96)
4 or more vs. First (ref.)	1.2	(0.86,1.67)	1.09	(0.83,1.43)	1.01	(0.65,1.57)	1.3	(0.92,1.83)	0.77	(0.56,1.05)
Maternal age at birth										
less than 20 vs. 30 or more (ref.)	1.5	(1.04,2.17)	1.18	(0.87,1.61)	1.11	(0.67,1.85)	2.03	(1.38,2.97)	1.07	(0.75,1.52)
20 to 24 vs. 30 or more (ref.)	1.19	(0.90,1.59)	0.88	(0.68,1.13)	1.08	(0.71,1.66)	1.21	(0.87,1.69)	0.99	(0.74,1.32)
25 to 29 vs. 30 or more (ref.)	0.88	(0.68,1.13)	0.9	(0.70,1.16)	0.73	(0.48,1.10)	0.82	(0.60,1.14)	0.75	(0.57,0.99)
Maternal education vs, Wealth										
Higher x richest (ref.)										
No Schooling x poorest	2.18	(0.91,5.22)	2.17	(1.14,4.13)	78.52	(20.20,305.13)	3.55	(1.26,9.99)	5.44	(2.14,13.82)
No Schooling x poorer	2.42	(0.99,5.90)	2.09	(1.09,3.99)	82.88	(21.43,320.51)	3.25	(1.15,9.20)	6.39	(2.52,16.20)
No Schooling x middle	1.37	(0.52,3.60)	1.54	(0.77,3.09)	82.05	(20.74,324.64)	3.52	(1.24,9.97)	5.37	(2.12,13.59)
No Schooling x richer	1.92	(0.68,5.41)	2.13	(0.88,5.15)	51.51	(12.03,220.44)	2.23	(0.77,6.47)	3.9	(1.52,10.04)
No Schooling x richest	1.41	(0.38,5.13)	0.57	(0.17,1.88)	2.6	(0.36,18.51)	1.04	(0.21,5.20)	3.21	(1.12,9.19)
Primary x poorest	2.04	(0.80,5.15)	2.58	(1.29,5.19)	90.98	(21.05,393.26)	4.58	(1.43,14.62)	2.72	(0.82,9.01)
Primary x poorer	1.85	(0.73,4.68)	1.2	(0.60,2.39)	72	(16.79,308.64)	2.36	(0.74,7.61)	3.34	(1.12,9.93)
Primary x middle	2.92	(1.01,8.43)	1.82	(0.89,3.70)	71.5	(16.52,309.52)	2.54	(0.86,7.55)	3.82	(1.40,10.42)
Primary x richer	2.84	(1.07,7.52)	1.73	(0.80,3.76)	58.86	(13.08,264.77)	1.88	(0.59,5.92)	1.96	(0.69,5.61)
Primary x richest	0.9	(0.21,3.89)	3.47	(0.93,12.96)	74.95	(12.14,462.88)	0.6	(0.13,2.73)	2.16	(0.64,7.30)
Middle x poorest	1.91	(0.76,4.80)	2.66	(1.35,5.23)	94.76	(19.73,455.06)	2.07	(0.41,10.49)	5.21	(1.33,20.43)
Middle x poorer	1.73	(0.70,4.29)	1.63	(0.83,3.20)	73.19	(16.73,320.14)	1.14	(0.26,5.05)	3.26	(0.77,13.80)
Middle x middle	1.31	(0.52,3.32)	1.89	(0.89,4.03)	56.89	(13.56,238.59)	2.47	(0.77,7.88)	3.84	(1.28,11.53)
Middle x richer	0.84	(0.31,2.26)	1.37	(0.53,3.49)	49.16	(11.53,209.54)	1.02	(0.28,3.76)	2.01	(0.71,5.68)
Middle x richest	0.93	(0.32,2.65)	0.88	(0.34,2.31)	44.69	(8.26,241.62)	1.05	(0.25,4.48)	1.76	(0.56,5.50)
Secondary x poorest	1.35	(0.55,3.31)	2.12	(0.98,4.58)	30.65	(2.85,329.41)	2.22	(0.24,20.56)	17.76	(3.99,79.08)
Secondary x poorer	1.51	(0.64,3.58)	2.03	(1.02,4.05)	97.54	(21.13,450.23)	1.9	(0.39,9.24)	1	(1.00,1.00)
Secondary x middle	1.08	(0.45,2.57)	1.12	(0.57,2.21)	55.18	(12.40,245.56)	1.74	(0.47,6.41)	3.76	(1.13,12.44)
Secondary x richer	1.16	(0.49,2.75)	0.86	(0.41,1.79)	21.89	(4.97,96.49)	0.58	(0.16,2.09)	3.64	(1.30,10.21)
Secondary x richest	0.84	(0.33,2.09)	0.79	(0.38,1.66)	27.63	(6.82,112.03)	1.37	(0.45,4.19)	1.56	(0.56,4.33)
Higher x poorest	1.03	(0.17,6.21)	1.1	(0.23,5.27)	1	(1.00,1.00)	1	(1.00,1.00)	1	(1.00,1.00)
Higher x poorer	1.17	(0.39,3.51)	0.93	(0.28,3.03)	1	(1.00,1.00)	1	(1.00,1.00)	1	(1.00,1.00)
Higher x middle	1.39	(0.55,3.51)	1.18	(0.49,2.86)	21.35	(1.96,232.74)	1	(1.00,1.00)	1	(1.00,1.00)
Higher x richer	0.53	(0.19,1.45)	0.67	(0.29,1.54)	55.02	(8.20,369.19)	0.16	(0.02,1.46)	1	(1.00,1.00)

Caste										
SC vs. Other (ref.)	0.99	(0.73,1.33)	1.09	(0.83,1.42)	1.13	(0.76,1.67)	1.18	(0.90,1.54)	0.91	(0.71,1.16)
ST vs. Other (ref.)	0.96	(0.69,1.33)	0.85	(0.64,1.14)	1.13	(0.73,1.76)	1.47	(1.08,1.99)	1.12	(0.86,1.45)
OBC vs. Other (ref.)	0.85	(0.66,1.11)	1.02	(0.80,1.30)	0.94	(0.66,1.33)	1.1	(0.86,1.41)		
Don't know vs. Other (ref.)	1.81	(0.39,8.47)	0.77	(0.31,1.95)	1.25	(0.17,9.00)				
No Caste vs. Other (ref.)										
Place of residence	1.11	[0.84,1.46]	1.06	[0.84,1.34]	1.12	[0.80,1.58]	0.95	[0.71,1.25]	1.17	[0.89,1.53]

OR: Odds Ratio; CI: Confidence Interval

Table S29: Adjusted Logistic Regression: Odds ratios and 95% confidence intervals for the association between covariates and post neonatal mortality (1993–2021), adjusted for twin birth, and the interaction between maternal education and wealth index.

	2019-21		2015-16		2005-06		1998-99		1992-93	
	OR	95 % CI	OR	95 % CI	OR	95 % CI	OR	95 % CI	OR	95 % CI
Sex of child										
Boy vs. Girls (ref.)	1.09	(0.98,1.22)	0.9	(0.82,1.00)	0.74	(0.62,0.88)	0.89	(0.78,1.01)	0.92	(0.81,1.04)
Birth order										
2 to 3 vs. First (ref.)	1.09	(0.94,1.27)	0.98	(0.85,1.13)	0.92	(0.73,1.18)	0.93	(0.77,1.12)	0.96	(0.80,1.14)
4 or more vs. First (ref.)	1.55	(1.26,1.91)	1.48	(1.23,1.79)	1.12	(0.82,1.54)	1.25	(0.98,1.59)	1.29	(1.03,1.61)
Maternal age at birth										
less than 20 vs. 30 or more (ref.)	1.84	(1.41,2.39)	1.33	(1.06,1.68)	1.77	(1.24,2.54)	1.65	(1.26,2.16)	1.25	(0.98,1.61)
20 to 24 vs. 30 or more (ref.)	1.43	(1.18,1.74)	0.91	(0.77,1.07)	1.16	(0.86,1.57)	1.2	(0.96,1.51)	1.09	(0.90,1.33)
25 to 29 vs. 30 or more (ref.)	1.18	(1.00,1.40)	0.89	(0.77,1.03)	1.15	(0.86,1.53)	1.08	(0.87,1.34)	0.76	(0.62,0.93)
Maternal education vs, Wealth										
Higher x richest (ref.)										
No Schooling x poorest	2.99	(1.70,5.25)	5.31	(3.45,8.16)	5.07	(2.17,11.84)	6.03	(2.62,13.85)	10.76	(4.15,27.91)
No Schooling x poorer	2.7	(1.53,4.76)	4.99	(3.23,7.73)	6.98	(3.02,16.13)	5.02	(2.19,11.51)	8.03	(3.09,20.89)
No Schooling x middle	3.23	(1.76,5.95)	4.31	(2.74,6.80)	5.31	(2.28,12.37)	4.45	(1.93,10.27)	7.33	(2.83,19.03)
No Schooling x richer	2.46	(1.28,4.73)	5.41	(3.24,9.04)	4.19	(1.74,10.09)	3.61	(1.55,8.41)	6.4	(2.47,16.60)
No Schooling x richest	1.14	(0.47,2.78)	3.62	(1.62,8.08)	5.51	(1.92,15.83)	4.06	(1.61,10.24)	6.26	(2.33,16.82)
Primary x poorest	3.35	(1.85,6.06)	4.27	(2.66,6.86)	5.05	(1.96,13.00)	5.31	(2.11,13.35)	8.84	(3.13,24.93)
Primary x poorer	1.93	(1.06,3.52)	4.77	(3.01,7.57)	4.32	(1.75,10.66)	4.84	(2.01,11.70)	4.71	(1.63,13.58)
Primary x middle	2.28	(1.17,4.44)	4.69	(2.85,7.73)	4.1	(1.66,10.12)	4.62	(1.96,10.92)	5.44	(1.99,14.85)
Primary x richer	2.67	(1.07,6.69)	3.15	(1.75,5.65)	1.53	(0.51,4.54)	2.52	(1.03,6.15)	6.02	(2.24,16.18)
Primary x richest	1.4	(0.53,3.65)	2.08	(1.00,4.32)	2.44	(0.56,10.63)	1.64	(0.55,4.87)	3.53	(1.22,10.24)
Middle x poorest	1.91	(1.05,3.48)	3.98	(2.45,6.47)	2.76	(0.82,9.27)	4.41	(1.44,13.50)	2.04	(0.46,9.05)
Middle x poorer	2.26	(1.25,4.09)	3.38	(2.14,5.36)	2.55	(0.92,7.08)	1.46	(0.44,4.81)	5.37	(1.66,17.39)
Middle x middle	1.64	(0.88,3.03)	3.65	(2.11,6.34)	3.34	(1.33,8.37)	2.78	(1.11,6.97)	5.88	(2.07,16.71)
Middle x richer	2.4	(1.26,4.59)	2.49	(1.47,4.23)	3.09	(1.22,7.82)	2.1	(0.84,5.22)	3.8	(1.39,10.44)
Middle x richest	0.88	(0.41,1.87)	2.56	(1.43,4.61)	2.16	(0.76,6.14)	2.46	(0.96,6.32)	4.63	(1.68,12.74)
Secondary x poorest	1.85	(1.02,3.37)	4.26	(2.54,7.17)	3.39	(0.69,16.68)	1	(1.00,1.00)	4.09	(0.45,37.25)
Secondary x poorer	1.64	(0.92,2.94)	3.34	(2.05,5.46)	3.18	(1.01,9.99)	2.28	(0.60,8.68)	4.8	(1.06,21.81)
Secondary x middle	1.61	(0.91,2.86)	3.14	(1.98,4.97)	1.33	(0.41,4.31)	1.85	(0.66,5.15)	5.92	(1.85,18.98)
Secondary x richer	1.49	(0.84,2.63)	2.27	(1.41,3.64)	2.27	(0.89,5.77)	1.78	(0.69,4.60)	4.08	(1.44,11.56)
Secondary x richest	0.99	(0.54,1.81)	2.1	(1.30,3.40)	1.5	(0.60,3.73)	1.26	(0.51,3.15)	2.15	(0.77,5.95)
Higher x poorest	1.11	(0.43,2.85)	2.79	(0.55,14.02)	48.42	(5.25,446.94)	1	(1.00,1.00)	1	(1.00,1.00)
Higher x poorer	1.68	(0.81,3.48)	3.64	(1.32,10.06)	1	(1.00,1.00)	1	(1.00,1.00)	1	(1.00,1.00)
Higher x middle	1.8	(0.96,3.37)	3.54	(1.73,7.25)	1	(1.00,1.00)	1	(1.00,1.00)	1	(1.00,1.00)
Higher x richer	0.95	(0.49,1.86)	1.63	(0.90,2.96)	1.4	(0.18,11.17)	1	(1.00,1.00)	1	(1.00,1.00)

Caste										
SC vs. Other (ref.)	1.1	(0.89,1.36)	1.05	(0.89,1.24)	0.95	(0.72,1.24)	1.06	(0.88,1.28)	1.17	(0.99,1.38)
ST vs. Other (ref.)	1.02	(0.82,1.27)	1.03	(0.85,1.24)	0.98	(0.71,1.34)	1.21	(0.97,1.50)	0.91	(0.75,1.11)
OBC vs. Other (ref.)	1	(0.82,1.21)	1.1	(0.95,1.28)	0.93	(0.72,1.18)	1.1	(0.93,1.30)		
Don't know vs. Other (ref.)	1.53	(0.62,3.80)	0.98	(0.58,1.66)	2.18	(0.95,5.01)				
No Caste vs. Other (ref.)										
Place of residence	0.91	[0.77,1.07]	1.02	[0.87,1.21]	1.03	[0.81,1.31]	1.02	[0.83,1.25]	0.94	[0.78,1.12]

OR: Odds Ratio; CI: Confidence Interval

Table S30: Adjusted Logistic Regression: Odds ratios and 95% confidence intervals for the association between covariates and child mortality (1993–2021), adjusted for twin birth, and the interaction between maternal education and wealth index.

	2019-21		2015-16		2005-06		1998-99		1992-93	
	OR	95 % CI	OR	95 % CI	OR	95 % CI	OR	95 % CI	OR	95 % CI
Sex of child										
Boy vs. Girls (ref.)	0.98	(0.81,1.19)	0.85	(0.72,1.01)	0.61	(0.47,0.79)	0.55	(0.46,0.65)	0.6	(0.51,0.71)
Birth order										
2 to 3 vs. First (ref.)	1.35	(1.05,1.74)	0.96	(0.76,1.21)	1.34	(0.93,1.94)	1.47	(1.13,1.91)	1.25	(0.98,1.59)
4 or more vs. First (ref.)	1.88	(1.33,2.65)	1.47	(1.13,1.91)	1.75	(1.14,2.68)	2.48	(1.79,3.44)	1.75	(1.29,2.38)
Maternal age at birth										
less than 20 vs. 30 or more (ref.)	1.25	(0.84,1.84)	0.87	(0.61,1.25)	1.5	(0.92,2.42)	1.64	(1.16,2.31)	1.33	(0.94,1.89)
20 to 24 vs. 30 or more (ref.)	1.01	(0.77,1.34)	0.92	(0.73,1.17)	1.12	(0.76,1.63)	1.21	(0.92,1.61)	1.3	(0.99,1.71)
25 to 29 vs. 30 or more (ref.)	0.79	(0.61,1.03)	0.92	(0.75,1.14)	1.01	(0.69,1.47)	0.81	(0.63,1.05)	1.17	(0.90,1.51)
Maternal education vs, Wealth										
Higher x richest (ref.)										
No Schooling x poorest	9.33	(4.22,20.67)	8.42	(3.02,23.49)	4.53	(1.05,19.54)	14.4	(3.01,68.81)	12.42	(2.11,72.91)
No Schooling x poorer	5.89	(2.64,13.13)	5.85	(2.08,16.45)	4.9	(1.13,21.23)	10.12	(2.13,48.23)	12.1	(2.06,71.08)
No Schooling x middle	4.51	(1.82,11.15)	5.33	(1.89,15.06)	3.92	(0.90,17.12)	7.85	(1.63,37.74)	8.78	(1.49,51.73)
No Schooling x richer	7.54	(2.89,19.63)	5.3	(1.71,16.39)	1.87	(0.39,8.88)	4.67	(0.95,23.00)	7.95	(1.34,47.14)
No Schooling x richest	6.16	(1.78,21.29)	3.69	(0.84,16.29)	2.57	(0.40,16.55)	1.68	(0.25,11.08)	4.62	(0.73,29.18)
Primary x poorest	5.38	(2.21,13.10)	5.53	(1.91,15.96)	3.88	(0.81,18.62)	6.42	(1.22,33.69)	7.41	(1.11,49.41)
Primary x poorer	4.96	(2.13,11.55)	7.58	(2.63,21.83)	2.25	(0.46,10.97)	7.99	(1.56,41.04)	4.7	(0.69,31.85)
Primary x middle	3.81	(1.52,9.53)	3.35	(1.12,9.97)	1.71	(0.35,8.46)	4.64	(0.89,24.14)	6.28	(1.00,39.39)
Primary x richer	3.13	(1.08,9.12)	6.6	(2.02,21.53)	1.85	(0.32,10.81)	3.28	(0.61,17.74)	3.53	(0.55,22.63)
Primary x richest	2.38	(0.37,15.06)	3.42	(0.95,12.40)	1	(1.00,1.00)	4.74	(0.83,27.16)	2.41	(0.33,17.47)
Middle x poorest	5.74	(2.46,13.35)	6.58	(2.27,19.07)	1	(1.00,1.00)	15.22	(2.72,85.14)	8.86	(1.11,70.69)
Middle x poorer	4.37	(1.80,10.60)	3.32	(1.10,10.02)	0.59	(0.09,3.77)	2.09	(0.30,14.69)	1.82	(0.21,16.11)
Middle x middle	5.1	(1.84,14.15)	3.29	(1.10,9.82)	1.36	(0.23,7.99)	5.48	(1.02,29.33)	8.23	(1.29,52.69)
Middle x richer	1.51	(0.57,3.99)	2.25	(0.75,6.73)	1.48	(0.26,8.46)	3.05	(0.58,16.16)	3.05	(0.46,20.40)
Middle x richest	3.51	(0.78,15.79)	1.42	(0.41,4.94)	0.73	(0.07,7.78)	1.31	(0.19,8.94)	1.72	(0.24,12.48)
Secondary x poorest	3.88	(1.59,9.49)	3.85	(1.23,12.07)	2.02	(0.19,21.55)	1	(1.00,1.00)	1	(1.00,1.00)
Secondary x poorer	4.22	(1.82,9.76)	2.38	(0.79,7.15)	2.48	(0.39,15.72)	5.51	(0.69,44.22)	1	(1.00,1.00)
Secondary x middle	2.29	(0.99,5.30)	3.24	(0.78,13.52)	0.48	(0.07,3.57)	5.82	(0.96,35.47)	1.73	(0.20,15.28)
Secondary x richer	1.56	(0.66,3.67)	2.04	(0.67,6.19)	0.47	(0.08,2.66)	1.88	(0.32,11.20)	3.71	(0.53,26.06)
Secondary x richest	1.73	(0.68,4.39)	1.15	(0.35,3.78)	0.8	(0.15,4.16)	1.56	(0.28,8.55)	0.96	(0.14,6.75)
Higher x poorest	1.12	(0.14,8.80)	3.48	(0.38,31.95)	1	(1.00,1.00)	1	(1.00,1.00)	1	(1.00,1.00)
Higher x poorer	1.51	(0.36,6.25)	0.72	(0.09,5.54)	1	(1.00,1.00)	1	(1.00,1.00)	1	(1.00,1.00)
Higher x middle	2.38	(0.85,6.71)	2.96	(0.78,11.17)	1	(1.00,1.00)	1	(1.00,1.00)	1	(1.00,1.00)
Higher x richer	2.37	(0.61,9.17)	1.19	(0.36,3.92)	1	(1.00,1.00)	4.56	(0.38,55.38)	1	(1.00,1.00)

Caste										
SC vs. Other (ref.)	1.28	(0.88,1.87)	1.2	(0.81,1.78)	1.62	(1.07,2.45)	0.99	(0.78,1.26)	1.13	(0.90,1.42)
ST vs. Other (ref.)	1	(0.68,1.46)	1.31	(0.90,1.91)	2.21	(1.43,3.41)	1.38	(1.07,1.77)	1.25	(0.99,1.59)
OBC vs. Other (ref.)	1.12	(0.79,1.58)	1.16	(0.80,1.69)	1.07	(0.72,1.60)	0.92	(0.73,1.16)		
Don't know vs. Other (ref.)	0.88	(0.15,5.07)	1.43	(0.65,3.17)	1	(1.00,1.00)				
No Caste vs. Other (ref.)										
Place of residence	0.8	[0.57,1.11]	0.87	[0.63,1.21]	0.95	[0.67,1.35]	0.8	[0.60,1.06]	0.96	[0.73,1.26]

OR: Odds Ratio; CI: Confidence Interval

Table S31: Adjusted Logistic Regression: Odds ratios and 95% confidence intervals for the association between covariates and early neonatal mortality (1993–2021), adjusted for twin birth, and the interaction between maternal education and caste.

	2019-21		2015-16		2005-06		1998-99		1992-93	
	OR	95 % CI	OR	95 % CI	OR	95 % CI	OR	95 % CI	OR	95 % CI
Sex of child										
Boy vs. Girls (ref.)	1.24	(1.15,1.34)	1.34	(1.25,1.43)	1.3	(1.14,1.49)	1.12	(1.00,1.25)	1.29	(1.15,1.44)
Birth order										
2 to 3 vs. First (ref.)	0.69	(0.63,0.76)	0.64	(0.59,0.70)	0.57	(0.48,0.68)	0.79	(0.68,0.92)	0.61	(0.53,0.71)
4 or more vs. First (ref.)	0.87	(0.75,1.01)	0.79	(0.70,0.89)	0.8	(0.62,1.03)	0.91	(0.73,1.12)	0.62	(0.50,0.77)
Maternal age at birth										
less than 20 vs. 30 or more (ref.)	1.27	(1.07,1.49)	1.19	(1.03,1.38)	1.59	(1.17,2.15)	1.42	(1.11,1.81)	1.26	(0.99,1.61)
20 to 24 vs. 30 or more (ref.)	0.98	(0.86,1.12)	0.99	(0.88,1.10)	1.31	(1.01,1.69)	1	(0.81,1.23)	0.92	(0.75,1.14)
25 to 29 vs. 30 or more (ref.)	0.89	(0.79,1.01)	0.85	(0.77,0.95)	0.94	(0.73,1.21)	0.84	(0.69,1.02)	0.9	(0.74,1.09)
Maternal education vs. Caste										
Higher x Other (ref.)										
No Schooling x SC	2.2	(1.44,3.36)	1.82	(1.38,2.41)	2.32	(1.22,4.42)	1.27	(0.75,2.15)	1.48	(0.89,2.46)
No Schooling x ST	1.68	(1.09,2.60)	1.32	(0.99,1.76)	1.71	(0.87,3.37)	1.04	(0.60,1.81)	1.05	(0.62,1.79)
No Schooling x OBC	2.05	(1.35,3.11)	1.82	(1.39,2.39)	2	(1.06,3.77)	1.33	(0.79,2.24)		
No Schooling x Other	1.74	(1.08,2.79)	1.7	(1.25,2.31)	1.97	(1.02,3.80)	1.02	(0.61,1.72)	1.14	(0.70,1.87)
No Schooling x Don't	3.18	(1.72,5.89)	2.1	(1.20,3.68)	0.67	(0.09,5.31)				
No Schooling x No caste	1.22	(0.65,2.30)	1.18	(0.78,1.79)	2.62	(1.19,5.77)	1.07	(0.49,2.34)		
Primary x SC	1.98	(1.27,3.07)	1.98	(1.46,2.68)	2.22	(1.11,4.44)	1.12	(0.62,2.04)	1.24	(0.65,2.36)
Primary x ST	1.63	(1.01,2.64)	1.76	(1.27,2.45)	1.65	(0.71,3.82)	0.62	(0.27,1.44)	1.19	(0.53,2.68)
Primary x OBC	1.75	(1.13,2.69)	1.91	(1.44,2.53)	2.15	(1.10,4.23)	1.05	(0.60,1.84)		
Primary x Other	2	(1.21,3.31)	1.35	(0.96,1.90)	2.16	(1.08,4.31)	0.95	(0.54,1.65)	0.99	(0.60,1.65)
Primary x Don't	2.15	(0.72,6.43)	1.06	(0.53,2.13)	1	(1.00,1.00)				
Primary x No caste	0.98	(0.52,1.85)	1.57	(0.91,2.71)	1	(0.32,3.09)	1	(1.00,1.00)		
Middle x SC	1.99	(1.29,3.06)	1.45	(1.07,1.97)	1.37	(0.66,2.84)	0.98	(0.50,1.89)	1.05	(0.48,2.30)
Middle x ST	1.8	(1.15,2.83)	1.3	(0.91,1.85)	0.7	(0.25,1.98)	1.11	(0.47,2.58)	0.8	(0.32,2.03)
Middle x OBC	1.81	(1.19,2.75)	1.8	(1.36,2.39)	1.39	(0.71,2.73)	0.92	(0.51,1.65)		
Middle x Other	1.51	(0.94,2.42)	1.55	(1.10,2.19)	1.61	(0.82,3.17)	0.92	(0.52,1.62)	0.93	(0.56,1.56)
Middle x Don't	1.8	(0.87,3.71)	1.52	(0.63,3.64)	3.16	(0.36,27.39)				
Middle x No caste	1.41	(0.76,2.64)	1.09	(0.62,1.89)	0.36	(0.07,1.88)	1	(1.00,1.00)		
Secondary x SC	1.52	(1.00,2.33)	1.21	(0.90,1.62)	1.49	(0.72,3.08)	0.91	(0.45,1.84)	0.11	(0.02,0.63)
Secondary x ST	1.45	(0.93,2.27)	1.29	(0.90,1.84)	1.12	(0.45,2.81)	1.5	(0.68,3.33)	0.88	(0.29,2.66)
Secondary x OBC	1.42	(0.94,2.15)	1.11	(0.84,1.46)	1.35	(0.71,2.58)	0.71	(0.39,1.29)		
Secondary x Other	1.29	(0.84,1.99)	1.09	(0.81,1.46)	1.35	(0.72,2.52)	0.88	(0.51,1.52)	0.87	(0.52,1.44)
Secondary x Don't	0.01	(0.00,0.10)	0.73	(0.22,2.46)	1	(1.00,1.00)				
Secondary x No caste	0.95	(0.55,1.62)	0.92	(0.57,1.50)	2.15	(0.76,6.10)	1.25	(0.18,8.73)		

Higher x SC	1.51	(0.94,2.43)	1.25	(0.85,1.84)	1.6	(0.44,5.78)	0.6	(0.08,4.56)	1	(1.00,1.00)
Higher x ST	1.09	(0.60,1.97)	0.87	(0.47,1.60)	1.3	(0.16,10.27)	3.75	(0.46,30.29)	1	(1.00,1.00)
Higher x OBC	1.1	(0.72,1.69)	1.01	(0.74,1.37)	0.91	(0.38,2.17)	0.63	(0.24,1.65)		
Higher x Don't	1	(1.00,1.00)	1	(1.00,1.00)	1	(1.00,1.00)				
Higher x No caste	0.32	(0.12,0.86)	0.24	(0.07,0.81)	0.05	(0.01,0.42)	1	(1.00,1.00)		
Household (Wealth Index)										
Poorest vs. Richest (ref.)	2.14	(1.75,2.62)	1.92	(1.61,2.29)	1.32	(0.94,1.86)	1.44	(1.10,1.89)	1.95	(1.47,2.58)
Poor vs. Richest (ref.)	2.01	(1.65,2.44)	1.7	(1.43,2.02)	1.15	(0.82,1.61)	1.2	(0.91,1.57)	1.81	(1.38,2.39)
Middle vs. Richest (ref.)	1.66	(1.37,2.01)	1.53	(1.30,1.81)	1.07	(0.78,1.48)	1.39	(1.08,1.80)	1.75	(1.34,2.29)
Rich vs. Richest (ref.)	1.56	(1.28,1.91)	1.31	(1.11,1.54)	1.1	(0.82,1.47)	1.23	(0.96,1.56)	1.39	(1.08,1.80)
Place of residence										
Rural vs. Urban (ref.)	1.09	(0.96,1.24)	1.21	(1.09,1.35)	1.14	(0.95,1.36)	1.12	(0.95,1.33)	1.03	(0.86,1.22)

OR: Odds Ratio; CI: Confidence Interval

Table S32: Adjusted Logistic Regression: Odds ratios and 95% confidence intervals for the association between covariates and late neonatal mortality (1993–2021), adjusted for twin birth, and the interaction between maternal education and caste.

	2019-21		2015-16		2005-06		1998-99		1992-93	
	OR	95 % CI	OR	95 % CI	OR	95 % CI	OR	95 % CI	OR	95 % CI
Sex of child										
Boy vs. Girls (ref.)	1.08	(0.91,1.28)	1.22	(1.05,1.42)	0.75	(0.58,0.96)	1.18	(0.98,1.43)	1.06	(0.89,1.25)
Birth order										
2 to 3 vs. First (ref.)	0.83	(0.67,1.03)	0.88	(0.71,1.08)	0.78	(0.56,1.08)	0.87	(0.68,1.12)	0.77	(0.61,0.97)
4 or more vs. First (ref.)	1.16	(0.86,1.57)	1.09	(0.83,1.44)	1.02	(0.66,1.59)	1.27	(0.90,1.79)	0.77	(0.56,1.06)
Maternal age at birth										
less than 20 vs. 30 or more (ref.)	1.51	(1.06,2.14)	1.21	(0.89,1.65)	1.13	(0.68,1.88)	1.98	(1.35,2.91)	1.07	(0.75,1.53)
20 to 24 vs. 30 or more (ref.)	1.18	(0.91,1.54)	0.89	(0.69,1.15)	1.1	(0.71,1.69)	1.2	(0.86,1.67)	0.99	(0.74,1.32)
25 to 29 vs. 30 or more (ref.)	0.88	(0.68,1.13)	0.9	(0.70,1.15)	0.74	(0.49,1.11)	0.82	(0.60,1.13)	0.75	(0.57,0.99)
Maternal education vs. Caste										
Higher x Other (ref.)										
No Schooling x SC	1.73	(0.94,3.20)	1.03	(0.47,2.28)	11.85	(2.89,48.64)	3.45	(0.67,17.61)	4.3	(1.48,12.45)
No Schooling x ST	1.26	(0.67,2.35)	0.93	(0.41,2.09)	13.07	(3.13,54.59)	4.37	(0.85,22.47)	4.59	(1.57,13.44)
No Schooling x OBC	1.6	(0.89,2.88)	1.07	(0.49,2.35)	10.98	(2.70,44.66)	3.55	(0.70,18.06)		
No Schooling x Other	1.35	(0.67,2.72)	0.78	(0.34,1.78)	13.73	(3.33,56.60)	3.12	(0.61,15.92)	4.33	(1.53,12.25)
No Schooling x Don't	0.56	(0.12,2.62)	1.6	(0.45,5.68)	25.58	(2.30,284.60)				
No Schooling x No caste	0.57	(0.19,1.71)	0.37	(0.13,1.05)	12.22	(2.61,57.16)	4.23	(0.70,25.58)		
Primary x SC	1.54	(0.80,2.98)	1.02	(0.45,2.30)	12.1	(2.57,56.90)	3.44	(0.63,18.87)	0.96	(0.21,4.33)
Primary x ST	1.55	(0.74,3.22)	0.73	(0.31,1.71)	9.09	(1.57,52.75)	4.51	(0.76,26.93)	2.27	(0.57,9.09)
Primary x OBC	1.66	(0.90,3.08)	1.12	(0.48,2.62)	15.07	(3.54,64.22)	1.79	(0.33,9.79)		
Primary x Other	1.97	(0.76,5.11)	1.09	(0.43,2.75)	11.58	(2.60,51.62)	3.12	(0.60,16.23)	2.74	(0.94,7.95)
Primary x Don't	1.17	(0.29,4.75)	0.48	(0.06,4.02)	1	(1.00,1.00)				
Primary x No caste	0.76	(0.25,2.28)	0.85	(0.25,2.86)	3.16	(0.29,34.98)	5.95	(0.47,75.06)		
Middle x SC	1.23	(0.66,2.30)	1.47	(0.63,3.42)	19.32	(4.35,85.82)	2.84	(0.48,16.79)	0.58	(0.08,4.32)
Middle x ST	1.47	(0.75,2.88)	0.75	(0.31,1.83)	15.3	(2.77,84.53)	2.61	(0.35,19.32)	9.26	(2.27,37.68)
Middle x OBC	0.88	(0.48,1.61)	1.01	(0.47,2.17)	7.19	(1.65,31.30)	2.02	(0.35,11.65)		
Middle x Other	1.65	(0.82,3.32)	0.78	(0.30,2.07)	10.15	(2.30,44.71)	1.17	(0.20,6.82)	2.59	(0.88,7.61)
Middle x Don't	0.53	(0.07,4.07)	1	(1.00,1.00)	1	(1.00,1.00)				
Middle x No caste	0.24	(0.06,1.05)	0.37	(0.12,1.08)	10.02	(1.52,66.23)	15.57	(1.69,143.93)		
Secondary x SC	1	(0.53,1.87)	0.82	(0.37,1.83)	12.58	(2.65,59.67)	2.38	(0.39,14.44)	0.16	(0.02,1.42)
Secondary x ST	1.24	(0.62,2.49)	0.66	(0.26,1.69)	15.63	(3.09,79.14)	1.62	(0.22,11.73)	4.79	(1.16,19.89)
Secondary x OBC	0.88	(0.50,1.56)	0.76	(0.35,1.63)	6.14	(1.45,26.07)	2.02	(0.36,11.43)		
Secondary x Other	0.89	(0.46,1.69)	0.9	(0.41,1.97)	5.74	(1.36,24.22)	1.4	(0.26,7.56)	3.15	(1.11,8.93)
Secondary x Don't	0.84	(0.19,3.78)	1	(1.00,1.00)	1	(1.00,1.00)				
Secondary x No caste	0.79	(0.29,2.11)	0.22	(0.06,0.83)	31.97	(6.05,168.84)	13.17	(1.10,157.76)		

Higher x SC	0.84	(0.33,2.18)	0.38	(0.12,1.24)	1	(1.00,1.00)	1	(1.00,1.00)	10.04	(1.37,73.42)
Higher x ST	0.78	(0.23,2.64)	0.69	(0.19,2.54)	1	(1.00,1.00)	10.25	(0.80,130.75)	1.83	(0.20,17.12)
Higher x OBC	0.51	(0.26,1.01)	0.62	(0.27,1.42)	7.35	(1.13,47.74)	2.6	(0.31,21.62)		
Higher x Don't	88.16	(10.18,763.20)	1	(1.00,1.00)	1	(1.00,1.00)				
Higher x No caste	0.74	(0.16,3.55)	1	(1.00,1.00)	1	(1.00,1.00)	1	(1.00,1.00)		
Household (Wealth Index)										
Poorest vs. Richest (ref.)	1.55	(1.05,2.30)	1.91	(1.27,2.86)	2.17	(1.12,4.18)	2.05	(1.27,3.31)	1.96	(1.24,3.11)
Poor vs. Richest (ref.)	1.58	(1.07,2.33)	1.54	(1.04,2.28)	2.19	(1.15,4.19)	1.73	(1.08,2.78)	2.2	(1.40,3.44)
Middle vs. Richest (ref.)	1.33	(0.88,2.01)	1.36	(0.92,2.01)	1.98	(1.05,3.71)	1.9	(1.19,3.02)	1.97	(1.27,3.05)
Rich vs. Richest (ref.)	1.2	(0.82,1.77)	1.11	(0.73,1.67)	1.33	(0.70,2.50)	1.07	(0.67,1.72)	1.37	(0.89,2.12)
Place of residence										
Rural vs. Urban (ref.)	1.14	(0.87,1.50)	1.05	(0.83,1.32)	1.13	(0.80,1.59)	0.93	(0.70,1.23)	1.16	(0.89,1.52)

OR: Odds Ratio; CI: Confidence Interval

Table S33: Adjusted Logistic Regression: Odds ratios and 95% confidence intervals for the association between covariates and post neonatal mortality (1993–2021), adjusted for twin birth, and the interaction between maternal education and caste.

	2019-21		2015-16		2005-06		1998-99		1992-93	
	OR	95 % CI	OR	95 % CI	OR	95 % CI	OR	95 % CI	OR	95 % CI
Sex of child										
Boy vs. Girls (ref.)	1.09	(0.98,1.22)	0.9	(0.82,1.00)	0.74	(0.62,0.88)	0.88	(0.78,1.01)	0.92	(0.81,1.04)
Birth order										
2 to 3 vs. First (ref.)	1.08	(0.94,1.25)	0.99	(0.86,1.14)	0.92	(0.72,1.17)	0.93	(0.77,1.12)	0.96	(0.80,1.14)
4 or more vs. First (ref.)	1.53	(1.26,1.87)	1.49	(1.23,1.80)	1.12	(0.81,1.53)	1.24	(0.98,1.58)	1.29	(1.03,1.61)
Maternal age at birth										
less than 20 vs. 30 or more (ref.)	1.85	(1.42,2.40)	1.34	(1.07,1.69)	1.77	(1.24,2.53)	1.65	(1.26,2.16)	1.26	(0.98,1.61)
20 to 24 vs. 30 or more (ref.)	1.43	(1.18,1.73)	0.92	(0.78,1.08)	1.17	(0.86,1.57)	1.2	(0.95,1.50)	1.09	(0.90,1.33)
25 to 29 vs. 30 or more (ref.)	1.18	(0.99,1.39)	0.89	(0.77,1.03)	1.14	(0.86,1.53)	1.08	(0.87,1.34)	0.76	(0.62,0.93)
Maternal education vs. Caste										
Higher x Other (ref.)										
No Schooling x SC	3.58	(2.04,6.27)	2.19	(1.29,3.72)	4.61	(1.73,12.27)	4.83	(1.69,13.81)	7.02	(2.69,18.35)
No Schooling x ST	3.68	(2.09,6.48)	2.02	(1.18,3.45)	4.38	(1.61,11.88)	5.74	(2.00,16.52)	5.23	(1.98,13.78)
No Schooling x OBC	3.85	(2.22,6.69)	2.45	(1.46,4.10)	4.31	(1.63,11.40)	4.79	(1.68,13.61)		
No Schooling x Other	3.47	(1.89,6.35)	2.07	(1.17,3.66)	5.02	(1.88,13.36)	4.63	(1.63,13.16)	5.83	(2.25,15.07)
No Schooling x Don't	4.63	(1.97,10.90)	1.86	(0.81,4.26)	8.02	(1.75,36.84)				
No Schooling x No caste	3.36	(1.56,7.21)	1.82	(0.97,3.42)	2.42	(0.76,7.74)	7.71	(2.44,24.36)		
Primary x SC	3.78	(2.10,6.79)	1.76	(1.01,3.07)	3.95	(1.40,11.13)	4.02	(1.34,12.06)	5.64	(1.95,16.30)
Primary x ST	2.7	(1.48,4.94)	1.6	(0.87,2.91)	3.92	(1.24,12.37)	3.57	(1.07,11.86)	4.28	(1.40,13.09)
Primary x OBC	3.28	(1.86,5.78)	2.18	(1.28,3.72)	2.39	(0.85,6.77)	5.11	(1.76,14.82)		
Primary x Other	4.28	(1.91,9.57)	2.11	(1.18,3.77)	2.61	(0.90,7.63)	3.53	(1.22,10.25)	4.34	(1.66,11.33)
Primary x Don't	2.3	(0.68,7.79)	1.95	(0.53,7.19)	20.23	(4.01,102.17)				
Primary x No caste	2.85	(1.19,6.86)	1.92	(0.67,5.53)	3.23	(0.83,12.64)	13.95	(2.90,67.11)		
Middle x SC	3.04	(1.69,5.47)	1.5	(0.85,2.62)	0.99	(0.26,3.73)	2.3	(0.67,7.93)	2.49	(0.71,8.68)
Middle x ST	3.01	(1.64,5.54)	1.76	(0.94,3.30)	2.26	(0.67,7.62)	2.59	(0.79,8.48)	5.79	(1.58,21.24)
Middle x OBC	2.55	(1.45,4.49)	1.56	(0.90,2.71)	3.07	(1.12,8.41)	2.65	(0.87,8.13)		
Middle x Other	2.59	(1.33,5.05)	1.4	(0.80,2.46)	3.27	(1.20,8.90)	2.77	(0.94,8.19)	3.99	(1.51,10.52)
Middle x Don't	0.93	(0.12,7.08)	2.61	(0.93,7.28)	0.29	(0.03,2.66)				
Middle x No caste	1.55	(0.66,3.65)	2.66	(1.09,6.50)	0.04	(0.00,0.32)	23.37	(4.99,109.36)		
Secondary x SC	2.5	(1.41,4.42)	1.79	(1.03,3.14)	1.78	(0.56,5.63)	2.62	(0.74,9.34)	1.89	(0.36,10.02)
Secondary x ST	2.14	(1.19,3.86)	1.85	(0.99,3.47)	2.8	(0.67,11.77)	1.22	(0.30,4.97)	2.68	(0.65,11.00)
Secondary x OBC	2	(1.15,3.46)	1.39	(0.84,2.32)	2.21	(0.79,6.14)	1.99	(0.64,6.15)		
Secondary x Other	2.41	(1.36,4.27)	1.35	(0.80,2.29)	1.49	(0.53,4.20)	1.85	(0.62,5.51)	3.14	(1.20,8.26)
Secondary x Don't	2.34	(0.65,8.40)	0.16	(0.02,1.25)	11.13	(1.26,98.59)				
Secondary x No caste	1.51	(0.63,3.60)	0.92	(0.46,1.84)	1	(1.00,1.00)	1	(1.00,1.00)		

Higher x SC	2.11	(1.07,4.17)	0.91	(0.44,1.90)	2.58	(0.29,22.55)	1	(1.00,1.00)	1	(1.00,1.00)
Higher x ST	2.67	(1.20,5.95)	1.77	(0.57,5.51)	0.12	(0.01,1.04)	1.12	(0.12,10.41)	1	(1.00,1.00)
Higher x OBC	1.9	(1.05,3.42)	1.02	(0.56,1.87)	1.23	(0.24,6.20)	1.92	(0.37,9.96)		
Higher x Don't	95.25	(11.24,806.94)	1	(1.00,1.00)	1	(1.00,1.00)				
Higher x No caste	3.71	(0.70,19.61)	0.55	(0.12,2.48)	4.92	(0.62,39.08)	1	(1.00,1.00)		
Household (Wealth Index)										
Poorest vs. Richest (ref.)	1.86	(1.44,2.40)	1.78	(1.36,2.33)	1.31	(0.80,2.13)	1.77	(1.26,2.49)	1.94	(1.45,2.61)
Poor vs. Richest (ref.)	1.62	(1.26,2.08)	1.69	(1.30,2.20)	1.64	(1.03,2.62)	1.47	(1.05,2.06)	1.43	(1.06,1.93)
Middle vs. Richest (ref.)	1.63	(1.26,2.12)	1.64	(1.25,2.15)	1.31	(0.83,2.06)	1.38	(0.99,1.91)	1.37	(1.03,1.84)
Rich vs. Richest (ref.)	1.54	(1.18,2.02)	1.28	(0.99,1.65)	1.07	(0.68,1.69)	1.03	(0.74,1.43)	1.22	(0.92,1.60)
Place of residence										
Rural vs. Urban (ref.)	0.92	(0.78,1.08)	1.03	(0.87,1.21)	1.04	(0.81,1.33)	1.03	(0.84,1.26)	0.94	(0.78,1.12)

OR: Odds Ratio; CI: Confidence Interval

Table S34: Adjusted Logistic Regression: Odds ratios and 95% confidence intervals for the association between covariates and child mortality (1993–2021), adjusted for twin birth, and the interaction between maternal education and caste.

	2019-21		2015-16		2005-06		1998-99		1992-93	
	OR	95 % CI	OR	95 % CI	OR	95 % CI	OR	95 % CI	OR	95 % CI
Sex of child										
Boy vs. Girls (ref.)	0.98	(0.81,1.19)	0.85	(0.72,1.01)	0.61	(0.48,0.79)	0.55	(0.46,0.65)	0.61	(0.51,0.72)
Birth order										
2 to 3 vs. First (ref.)	1.35	(1.04,1.74)	0.96	(0.76,1.21)	1.35	(0.93,1.94)	1.46	(1.12,1.90)	1.25	(0.98,1.59)
4 or more vs. First (ref.)	1.88	(1.33,2.65)	1.48	(1.14,1.91)	1.75	(1.14,2.69)	2.47	(1.78,3.43)	1.75	(1.29,2.38)
Maternal age at birth										
less than 20 vs. 30 or more (ref.)	1.25	(0.84,1.84)	0.88	(0.62,1.25)	1.51	(0.93,2.43)	1.64	(1.16,2.31)	1.33	(0.93,1.89)
20 to 24 vs. 30 or more (ref.)	1.02	(0.77,1.34)	0.93	(0.73,1.17)	1.12	(0.77,1.64)	1.21	(0.92,1.60)	1.3	(0.99,1.71)
25 to 29 vs. 30 or more (ref.)	0.79	(0.61,1.03)	0.92	(0.74,1.14)	1.01	(0.69,1.47)	0.81	(0.62,1.05)	1.17	(0.90,1.51)
Maternal education vs. Caste										
Higher x Other (ref.)										
No Schooling x SC	6.67	(2.23,19.90)	3.78	(1.19,12.01)	1.7	(0.41,7.04)	1.64	(0.43,6.29)	5.03	(0.84,29.99)
No Schooling x ST	5.22	(1.74,15.71)	4.89	(1.53,15.58)	2.32	(0.56,9.60)	2.33	(0.60,9.01)	5.81	(0.97,34.67)
No Schooling x OBC	6.16	(2.07,18.35)	4.09	(1.30,12.91)	1.08	(0.27,4.41)	1.42	(0.37,5.44)		
No Schooling x Other	5.54	(1.70,18.03)	2.73	(0.84,8.91)	1.19	(0.29,4.90)	1.53	(0.40,5.87)	4.55	(0.77,26.88)
No Schooling x Don't	1.14	(0.19,6.71)	4.44	(0.89,22.21)	1	(1.00,1.00)				
No Schooling x No caste	0.69	(0.14,3.40)	2.68	(0.70,10.27)	1.5	(0.29,7.73)	3.02	(0.71,12.85)		
Primary x SC	4.17	(1.32,13.18)	3.23	(0.96,10.94)	1.23	(0.28,5.49)	0.76	(0.17,3.41)	4.18	(0.63,27.87)
Primary x ST	1.98	(0.56,7.03)	3.71	(1.12,12.26)	1.47	(0.29,7.61)	0.73	(0.16,3.40)	1.77	(0.19,16.16)
Primary x OBC	3.76	(1.23,11.49)	4.01	(1.24,13.02)	0.7	(0.15,3.23)	1.15	(0.28,4.83)		
Primary x Other	3.81	(1.15,12.63)	3.03	(0.86,10.71)	0.5	(0.10,2.61)	1.22	(0.31,4.88)	2.33	(0.39,14.10)
Primary x Don't	13.81	(1.54,123.99)	4.56	(0.71,29.36)	1	(1.00,1.00)				
Primary x No caste	2.34	(0.47,11.56)	0.81	(0.16,4.16)	1	(1.00,1.00)	1.98	(0.20,20.10)		
Middle x SC	4.02	(1.26,12.79)	2.7	(0.80,9.09)	0.32	(0.05,2.09)	0.67	(0.14,3.30)	3.01	(0.36,25.21)
Middle x ST	4.06	(1.27,13.02)	1.98	(0.57,6.87)	0.81	(0.13,5.24)	0.83	(0.12,5.93)	2.45	(0.29,20.58)
Middle x OBC	4.08	(1.30,12.84)	2.24	(0.69,7.26)	0.54	(0.11,2.79)	1.06	(0.25,4.45)		
Middle x Other	3.88	(0.99,15.27)	2.28	(0.68,7.63)	0.14	(0.01,1.33)	1.04	(0.26,4.22)	2.29	(0.38,13.87)
Middle x Don't	1	(1.00,1.00)	4.28	(0.91,20.05)	1	(1.00,1.00)				
Middle x No caste	0.85	(0.20,3.61)	1.58	(0.36,6.88)	1	(1.00,1.00)	1.94	(0.17,21.62)		
Secondary x SC	3.06	(1.00,9.32)	2.26	(0.65,7.85)	0.54	(0.10,2.81)	1.06	(0.19,6.00)	0.18	(0.01,2.59)
Secondary x ST	2.94	(0.93,9.27)	1.55	(0.45,5.32)	0.42	(0.05,3.62)	1.11	(0.15,8.05)	1.72	(0.24,12.41)
Secondary x OBC	2.37	(0.79,7.11)	1.51	(0.47,4.85)	0.21	(0.03,1.32)	0.75	(0.16,3.61)		
Secondary x Other	2.22	(0.71,6.97)	1.98	(0.41,9.59)	0.35	(0.07,1.82)	0.75	(0.18,3.07)	1.43	(0.23,9.03)
Secondary x Don't	1	(1.00,1.00)	1.11	(0.11,10.70)	1	(1.00,1.00)				
Secondary x No caste	2.43	(0.53,11.03)	1.45	(0.32,6.63)	1.06	(0.14,8.33)	1	(1.00,1.00)		

Higher x SC	4.26	(0.88,20.69)	2.66	(0.55,12.86)	1	(1.00,1.00)	1	(1.00,1.00)	1	(1.00,1.00)
Higher x ST	1.91	(0.41,8.81)	1.53	(0.27,8.65)	1	(1.00,1.00)	1	(1.00,1.00)	1	(1.00,1.00)
Higher x OBC	1.7	(0.55,5.31)	0.98	(0.26,3.60)	1	(1.00,1.00)	1	(1.00,1.00)		
Higher x Don't	1	(1.00,1.00)	1	(1.00,1.00)	1	(1.00,1.00)				
Higher x No caste	0.18	(0.02,1.63)	1	(1.00,1.00)	1	(1.00,1.00)	1	(1.00,1.00)		
Household (Wealth Index)										
Poorest vs. Richest (ref.)	2.15	(1.24,3.72)	2.9	(1.54,5.46)	1.74	(0.88,3.47)	4.77	(2.63,8.67)	3.09	(1.86,5.14)
Poor vs. Richest (ref.)	1.66	(0.97,2.81)	2.2	(1.20,4.03)	1.79	(0.90,3.55)	3.39	(1.87,6.13)	2.89	(1.75,4.76)
Middle vs. Richest (ref.)	1.37	(0.79,2.39)	1.93	(0.96,3.89)	1.38	(0.70,2.71)	2.68	(1.47,4.90)	2.33	(1.42,3.83)
Rich vs. Richest (ref.)	1.1	(0.62,1.95)	1.69	(1.01,2.83)	0.92	(0.45,1.88)	1.57	(0.88,2.81)	1.92	(1.18,3.14)
Place of residence										
Rural vs. Urban (ref.)	0.8	(0.58,1.11)	0.86	(0.62,1.21)	0.95	(0.67,1.36)	0.8	(0.60,1.06)	0.96	(0.73,1.26)

OR: Odds Ratio; CI: Confidence Interval
